# Identification of miRNAs and their targets in wild tomato at moderately and acutely elevated temperatures by high-throughput sequencing and degradome analysis

**DOI:** 10.1038/srep33777

**Published:** 2016-09-22

**Authors:** Rong Zhou, Qian Wang, Fangling Jiang, Xue Cao, Mintao Sun, Min Liu, Zhen Wu

**Affiliations:** 1 College of Horticulture, Nanjing Agricultural University, Weigang No. 1, Nanjing, 210095, China; 2 Vegetable Research Institute, Jiangsu Academy of Agricultural Science, Zhongling Street No. 50, Nanjing, 210014, China; 3 Laboratory for Genetic Improvement of High Efficiency Horticultural Crops in Jiangsu province, Jiangsu, Nanjing, China; 4 Key Laboratory of Horticultural Plant Biology and Germplasm Innovation in East China, Ministry of Agriculture, Nanjing, 210095, China

## Abstract

MicroRNAs (miRNAs) are 19–24 nucleotide (nt) noncoding RNAs that play important roles in abiotic stress responses in plants. High temperatures have been the subject of considerable attention due to their negative effects on plant growth and development. Heat-responsive miRNAs have been identified in some plants. However, there have been no reports on the global identification of miRNAs and their targets in tomato at high temperatures, especially at different elevated temperatures. Here, three small-RNA libraries and three degradome libraries were constructed from the leaves of the heat-tolerant tomato at normal, moderately and acutely elevated temperatures (26/18 °C, 33/33 °C and 40/40 °C, respectively). Following high-throughput sequencing, 662 conserved and 97 novel miRNAs were identified in total with 469 conserved and 91 novel miRNAs shared in the three small-RNA libraries. Of these miRNAs, 96 and 150 miRNAs were responsive to the moderately and acutely elevated temperature, respectively. Following degradome sequencing, 349 sequences were identified as targets of 138 conserved miRNAs, and 13 sequences were identified as targets of eight novel miRNAs. The expression levels of seven miRNAs and six target genes obtained by quantitative real-time PCR (qRT-PCR) were largely consistent with the sequencing results. This study enriches the number of heat-responsive miRNAs and lays a foundation for the elucidation of the miRNA-mediated regulatory mechanism in tomatoes at elevated temperatures.

A plant exposed to a temperature elevated above a threshold level is said to be heat stressed^1^. The ambient global temperature has increased by 0.5 °C over the past 100 years, and is expected to increase by 0.2 °C per decade for the next two decades and 1–3.4 °C by 2100^2^. Heat stress induces morphological, anatomical, metabolic, physiological and genetic responses that could negatively affect plant growth, development and yield. For this reason, the problem of heat stress has drawn increasing attention in recent years^1^,^3^.

The tomato (*Solanum lycopersicum* L.) is not only an important and widely cultivated vegetable but also a model plant for scientific research^4^. Although the tomato originates from South America, both its vegetative and reproductive organs can be damaged by heat stress, with consequent decreases in yield and fruit quality^5^,^6^,^7^. Tomato plants make complex adjustments to physiological, metabolic and molecular processes involving reactive oxygen species (ROS) scavengers, hormones, heat-shock transcription factors (Hsfs) and heat-shock proteins (Hsps) to survive under high temperatures^8^,^9^,^10^.

microRNAs (miRNAs), single-stranded RNA molecules with 19–24 nucleotides (nt) in length, play crucial regulatory roles in animals and plants^11^,^12^. MiRNAs are involved in regulating the responses of plants to abiotic stresses, such as cold and salt stress^13^,^14^. In plants, the miRNA gene is transcribed into a primary miRNA (pri-miRNA) and the pri-miRNA is trimmed into a miRNA precursor (pre-miRNA)^15^. Afterwards, the pre-miRNA is processed by Dicer-like (DCL) into miRNA::miRNA^*^ duplex^11^,^15^. Finally, the miRNA^*^ strand is usually degraded, while the mature miRNA joins with argonaute (AGO) to form the RNA-induced silencing complex (RISC)^11^. The major approaches for miRNAs identification in plants are direct cloning, bioinformatics-based prediction and sequencing^13^,^16^. Sequencing, especially high-throughput sequencing, is one of the most efficient approaches and has allowed a multitude of novel miRNAs with low abundance to be identified in plants such as *Arabidopsis thaliana*^17^, *Porphyra yezoensis*^18^, and *Brassica napus*^19^.

It is important not only to accurately identify the miRNAs but also to identify their target genes and elucidate the interactions between the miRNAs and their targets. The identification of miRNA targets is a key component of a thorough understanding of the miRNAs’ biological functions and regulatory mechanism^20^. MiRNAs regulate the expression of target genes at the post-transcriptional level by mRNA cleavage or translational inhibition^11^,^21^. Usually, miRNAs in plants show extensive complementarity to the coding or 3′ untranslated regions (3′ UTR) of the target transcripts; therefore, mRNA cleavage is thought to be the primary mechanism underlying miRNA functions in plants^21^,^22^. Computational target prediction, 5′ RACE and degradome sequencing are used to identify miRNA targets^16^,^18^,^23^. Degradome sequencing is a powerful tool because of its efficiency and accuracy and has been widely used in various plants, including *Cucumis sativus*^20^ and *Brassica Campestris* ssp. *Chinensis*^24^.

In our previous study, miRNAs responsive to low temperature and their targets were identified in the leaves of a chill-tolerant wild tomato (*Solanum habrochaites* L.) using high-throughput sequencing and degradome analysis^25^. A global identification of miRNAs and their target genes will lay the foundation for the elucidation of the complex miRNA-mediated network regulating plant growth and development^26^. miR398 is rapidly induced in *Arabidopsis* subjected to high temperature and its target genes are inhibited, indicating miR398 play roles in heat stress responsed in plants^27^. The expression profile of some miRNAs was altered in *Oryza sativa* under heat stress^28^. Heat-responsive miRNAs has been identified in *Triticum aestivum*, showing that diverse set of wheat miRNAs were responsive to high temperature and played roles in heat stress^26^. However, there have been no reports of a global identification of heat-responsive miRNAs and their targets in tomatoes, especially at different elevated temperatures.

The degree of damage caused by exposure to high temperatures to a tomato plant depends on the heat susceptibility of the tomato genotype^29^,^30^. In our previous study, the wild tomato LA2093 (*Solanum pimpinellifolium* L.) was identified as a heat-tolerant tomato with protective mechanisms active at high temperature^30^. Cellular damage or cell death may occur in plants after long-term exposure to moderately elevated temperatures, but severe damage and death may occur within minutes at acutely elevated temperatures^1^. Therefore, we first constructed three small-RNA (sRNA) libraries and three degradome libraries from the leaves of the heat-tolerant wild tomato at normal, moderately elevated and acutely elevated temperatures. The conserved and novel miRNAs in the three sRNA libraries were identified using high-throughput sequencing, and the miRNA targets were identified using degradome analysis. Our hypothesis is that (1) the biogenesis of some miRNAs is affected by elevated temperatures, and these miRNAs might play roles in heat tolerance in tomato plants by regulating the expression of their target genes; and (2) the responses of the miRNAs are altered at different elevated temperatures. The aim of this study is to (1) identify the miRNAs that respond to moderately and acutely elevated temperatures in tomatoes by high-throughput sequencing (2) discover the targets of these miRNAs and annotate their functions by degradome analysis; (3) validate the expression levels of the miRNAs and their target genes using quantitative real-time PCR (qRT-PCR). This study will help to clarify the responses of miRNAs and their target genes in tomato plants at moderately and acutely elevated temperatures and the miRNA-regulated mechanism of heat tolerance in tomato plants.

## Results

### Overview of sRNA sequencing data

High-throughput sequencing identified 10,850,996, 10,806,944 and 10,989,848 raw reads in the plants at normal temperature (NT), moderately elevated temperature (MET) and acutely elevated temperature (AET), respectively (Table 1). Of the total sRNAs, 34.28%, 33.51% and 38.63% (reads/reads) were filtered out in the NT, MET and AET libraries, respectively. After the redundant reads were screened out and removed, the 3,333,687, 3,964,329 and 4,483,258 valid reads in the NT, MET and AET libraries corresponded to 829,756, 1,095,508 and 560,147 unique reads, respectively (Table 1).

**Figure 1:**
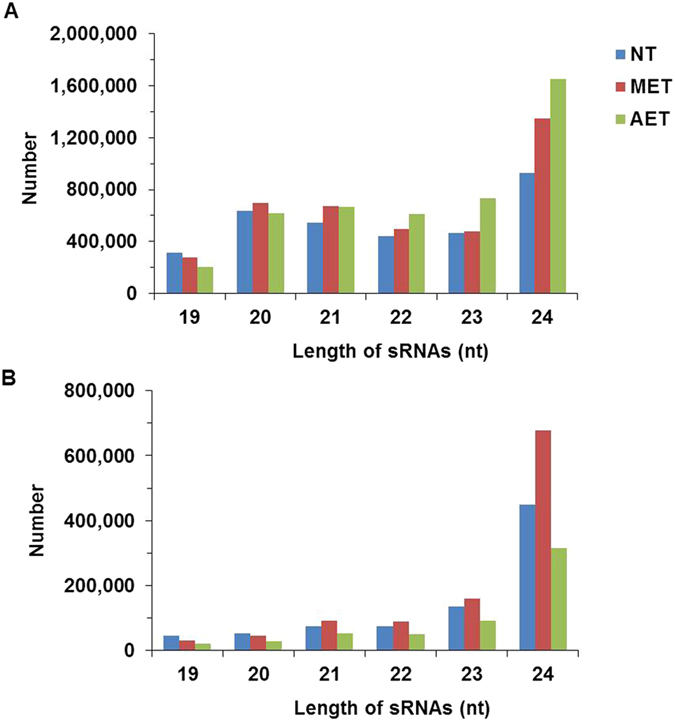
The length distribution of sRNAs in the wild tomato at a control temperature (26/18 °C, NT), moderately elevated temperature (33/33 °C, MET) and acutely elevated temperature (40/40 °C, AET). (**A**) Total sequences; (**B**) unique sequences.

Among all of the sequences, 24-nt sRNAs were the main class of sRNAs, accounting for 27.89%, 33.99% and 36.81% of all reads in the NT, MET and AET libraries, respectively (Fig. 1A). Among the unique sequences, 24-nt sRNAs represented the greatest proportion of sequences, constituting 54.12%, 61.77% and 56.35% in the NT, MET and AET libraries, respectively (Fig. 1B).

### Identification of conserved and novel miRNAs in tomato

**Figure 2:**
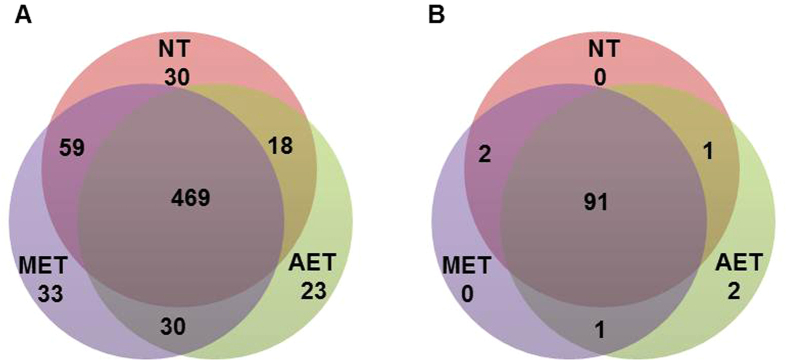
Venn diagrams of (**A**) conserved miRNAs and (**B**) novel miRNAs identified in the wild tomato at a control temperature (26/18 °C, NT), moderately elevated temperature (33/33 °C, MET) and acutely elevated temperature (40/40 °C, AET).

Some miRNAs shared the same precursors, such as spi-miR156d-5p and spi-miR156d-3p, spi-miR156a and spi-miR156i-p3_nta and so on (Supplementary Table S1). After statistic, for tomato, 662 conserved miRNAs from 532 miRNA precursors and 97 novel miRNAs from 92 miRNA precursors in total were identified (Supplementary Table S1). Pre-miRNA sequences of the miRNAs were shown in Supplementary Table S2. The majority of the conserved miRNAs (469/662) and novel miRNAs (91/97) were shared in the three libraries, which accounted for 70.8% and 93.8%, respectively (Fig. 2). The folding structures of the novel miRNAs were shown in Supplementary Figure S1.

Firstly, 39 conserved miRNAs were significantly up-regulated and 38 conserved miRNAs were significantly down-regulated in the MET library compared to the NT library (Supplementary Table S3). Of these miRNAs, the most-up-regulated miRNA was spi-miR5303b-p3_stu (2.79-fold), while the most-down-regulated miRNA was spi-miR166m_gma (4.44-fold). Secondly, the expression levels of 62 conserved miRNAs significantly increased and the expression levels of 57 conserved miRNAs significantly decreased in the AET library compared to the NT library (Supplementary Table S3). Of these miRNAs, the expression level of spi-miR7972_rgl increased most (3.89-fold), while the expression level of spi-miR8155_cpa decreased most (4.27-fold). Thirdly, 50 conserved miRNAs showed significantly increased expression and 60 conserved miRNAs showed significantly decreased expression in the AET library compared to the MET library (Supplementary Table S3). Of these miRNAs, the expression level of spi-miR7972_rgl increased most (4.57-fold), while the expression level of spi-miR172d-3p_stu decreased most (4.38-fold).

Similar to the conserved miRNAs, the expression levels of many novel miRNAs showed significant differences across libraries. Firstly, four novel miRNAs were found to be significantly up-regulated and 15 novel miRNAs were found to be significantly down-regulated in the MET library compared to the NT library (Supplementary Table S4). Of these miRNAs, the most-up-regulated miRNA was PC-70-5p (2.59-fold), while the most-down-regulated miRNA was PC-19-5p (2.67-fold). Secondly, 20 novel miRNAs showed significantly increased expression, while 11 novel miRNAs showed significantly decreased expression in the AET library compared to the NT library (Supplementary Table S4). Of these miRNAs, PC-27-5p increased most (3.89-fold), while PC-72-5p and PC-88-3p decreased most (3.57-fold). Thirdly, the expression levels of 30 novel miRNAs were significantly increased and five novel miRNAs were significantly decreased in the AET library compared to the MET library (Supplementary Table S4). Of these miRNAs, the expression level of PC-10-5p increased most (4.69-fold), while that of PC-15-5p decreased most (3.48-fold).

**Figure 3:**
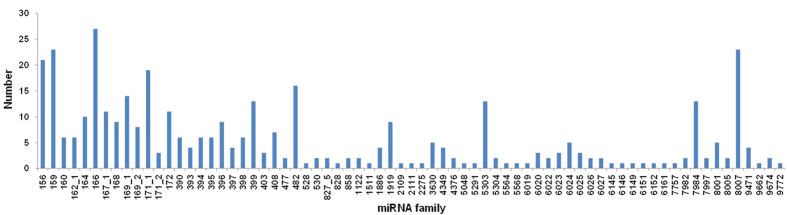
Number of miRNAs in each miRNAs family in the wild tomato.

Of the conserved miRNAs detected in the wild tomato LA2093, 390 miRNAs were assigned to 71 miRNA families by family analysis (Fig. 3). Of the 71 families, miR166 was the largest, with 27 miRNA members, followed by miR159, miR8007 and miR156, with 23, 23 and 21 miRNA members, respectively (Fig. 3). There were 20 families with only one member, such as miR528 and miR828 (Fig. 3).

### Overview of degradome sequencing data

There were 11,774,232, 11,471,728 and 10,853,424 raw reads identified in the NT, MET and AET libraries by degradome sequencing, corresponding to 3,596,303, 3,379,825 and 3,454,547 unique raw reads, respectively (Table 2). After blasting, 80.19%, 78.75% and 80.90% of the reads in the NT, MET and AET libraries, respectively, mapped to the reference data (ftp.jgi-psf.org/pub/compgen/phytozome/.v10.0/Slycopersicum_225_iTAGv2.3/annotation/Slycopersicum_225_iTAGv2.3.transcript.fa.gz) (Table 2).

### Target genes identification of conserved and novel miRNAs in tomato

Of the 662 conserved miRNAs, 138 conserved miRNAs targeted 349 sequences of 163 specific genes (Supplementary Table S5). Additionally, 73 target sequences of the conserved miRNAs were detected in all three libraries, but 67, 56 and 66 target sequences were detected only in the NT, MET and AET libraries, respectively (Supplementary Table S5). The NT library contained 212 target sequences of the conserved miRNAs, including 19, 8, 62, 0 and 123 sequences belonging to categories 0, 1, 2, 3 and 4, respectively. The MET library contained 186 target sequences of the conserved miRNAs, including18, 10, 57, 12 and 89 sequences belonging to categories 0, 1, 2, 3 and 4, respectively. Moreover, the AET library contained 184 target sequences of the conserved miRNAs, including 24, 6, 52, 1 and 101 sequences from categories 0, 1, 2, 3 and 4, respectively. A single conserved miRNA could regulate several target genes and more than one conserved miRNA could regulate one target gene. For example, spi-miR6300_gma targeted both *HSP70* and *HMT*; *HSP60-3A* was targeted by both spi-miR166g-3p_osa and spi-miR166c-3p; SPL9 was targeted by spi-miR156a, spi-miR156a_stu, spi-miR156f-5p_stu and spi-miR157d_ath.

Of the 97 novel miRNAs, eight novel miRNAs targeted 13 sequences of ten specific genes, with only one sequence targeted by PC-69-3p detected in all three libraries (Supplementary Table S6). The NT, MET and AET libraries contained eight (one from category 2 and seven from category 4), five (two from category 2 and three from category 4) and three (all from category 2) target sequences, respectively. The target genes could be regulated by both the conserved and the novel miRNAs. For example, *RLP32* might be regulated by spi-miR6022 and PC-49-3p. Compared to the conserved miRNAs, the novel miRNAs targeted some noteworthy genes. S-adenosyl-L-methionine-dependent methyltransferases are the key enzymes in the synthesis of phenylpropanoids, flavonoids and many other metabolic products in plants^31^. *AR401* (S-adenosyl-L-methionine-dependent methyltransferase superfamily protein) might be regulated by PC-69-3p, which was detected in all three libraries in this study. Pentatricopeptide repeat (PPR) protein is involved in RNA editing, RNA splicing, RNA cleavage and translation and plays essential roles in posttranscriptional processes in mitochondria and chloroplasts^32^. *CRR4* (PPR superfamily protein) might be regulated by PC-84-5p, which was detected only in the MET library.

### Functional analysis of miRNA targets in tomato

The GO functional annotation suggested that the target genes of conserved miRNAs were involved in many biological processes and molecular functions, including apoptotic process, oxidation-reduction process, protein phosphorylation, DNA-dependent regulation of transcription, ATP binding and so on (Supplementary Figure S2). Compared to the conserved miRNAs, the target genes of the novel miRNAs had some distinctive molecular functions, including nitronate monooxygenase, aspartic-type endopeptidase and methyltransferase activity (Supplementary Table S7). The KEGG analysis revealed few functions for the conserved miRNA targets, such as citrate cycle (TCA cycle), carbon fixation in photosynthetic organisms and so on (Supplementary Table S8).

The GO enrichment analysis showed that the target genes of the miRNAs with different expression levels were mainly involved in sequence-specific DNA-binding transcription-factor activity, regulation of transcription and sequence-specific DNA binding in the wild tomato plants at the moderately and acutely elevated temperatures (Supplementary Figure S3).

### qRT-PCR validation of miRNAs and target genes in tomato

**Figure 4:**
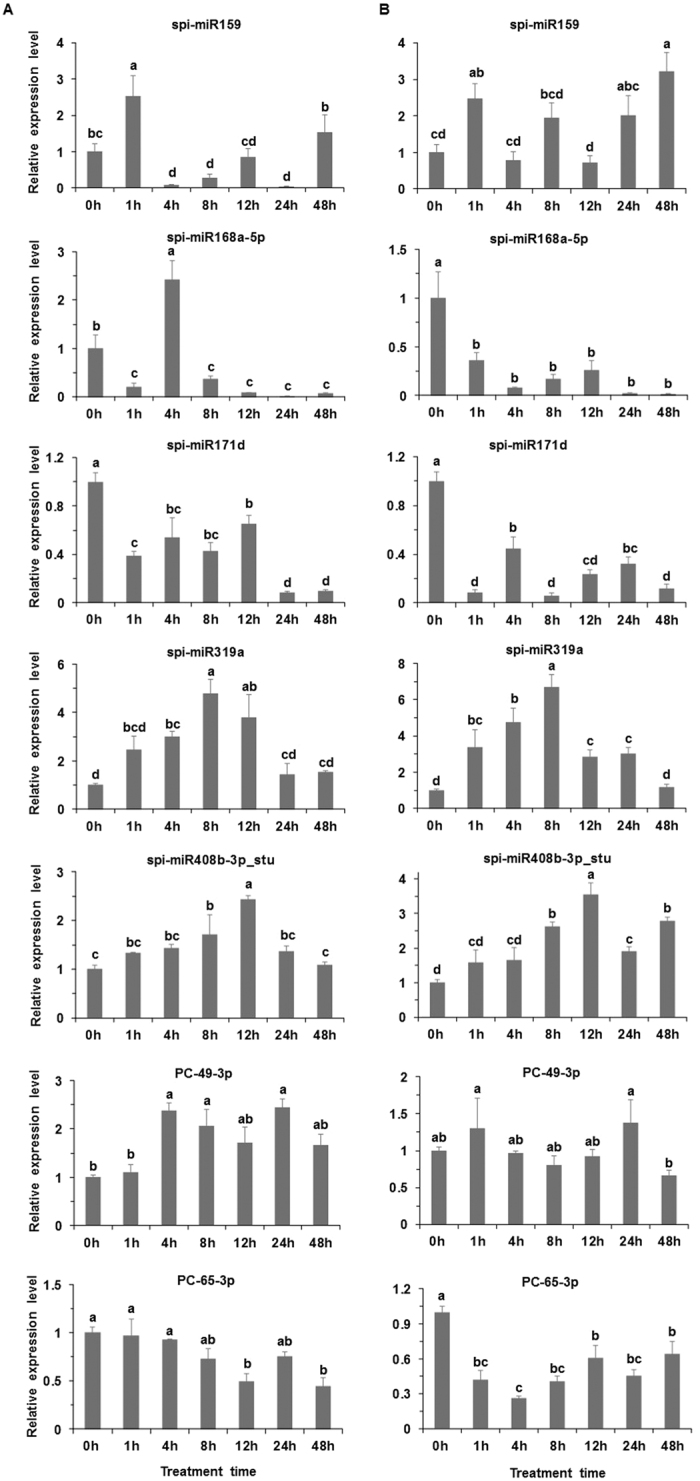
qRT-PCR validation of miRNAs in the wild tomato at control (0 h) or elevated temperature (1 h, 4 h, 8 h, 12 h, 24 h and 48 h). (**A**) Left, moderately elevated temperature (33/33 °C, MET); (**B**) right, acutely elevated temperature (40/40 °C, AET). The seven sub-graphs in A and B give the values for seven miRNAs. Each value was calculated as the mean of three samples ± standard error (SE). The different letters above the bar indicate significant difference (*P* < 0.05).

Among the conserved miRNAs, compared to the respective control, the expression level of spi-miR159 significantly increased at 1 h but decreased at 4 h, 8 h and 24 h at the moderately elevated temperature; the expression level of spi-miR168a-5p significantly decreased at the moderately elevated temperature except at 4 h; the expression level of spi-miR171d significantly decreased at the moderately elevated temperature (Fig. 4A). Compared to the control, the expression level of spi-miR319a was significantly higher at 4 h, 8 h and 12 h at the moderately elevated temperature; the expression level of spi-miR408b-3p_stu was significantly higher at 8 h and 12 h at the moderately elevated temperature (Fig. 4A). Among the novel miRNAs, compared to the control, the expression level of PC-49-3p significantly increased at 4 h, 8 h and 24 h at the moderately elevated temperature; the expression level of PC-65-3p significantly decreased at 12 h and 48 h at the moderately elevated temperature (Fig. 4A). The expression level of spi-miR159 was significantly higher at 1 h and 48 h, while the expression level of spi-miR168a-5p and spi-miR171d was significantly lower at the acutely elevated temperature as compared to the control (Fig. 4B). Compared to the control, the expression level of spi-miR319a significantly increased at 1 h, 4 h, 8 h, 12 h and 24 h at the acutely elevated temperature; the expression level of spi-miR408b-3p_stu was significantly higher at 8 h, 12 h, 24 h and 48 h at the acutely elevated temperature (Fig. 4B). The expression level of PC-49-3p showed no significant difference at different time points of at acutely elevated temperature; the expression level of PC-65-3p significantly decreased at the acutely elevated temperature compared to the control (Fig. 4B).

**Figure 5:**
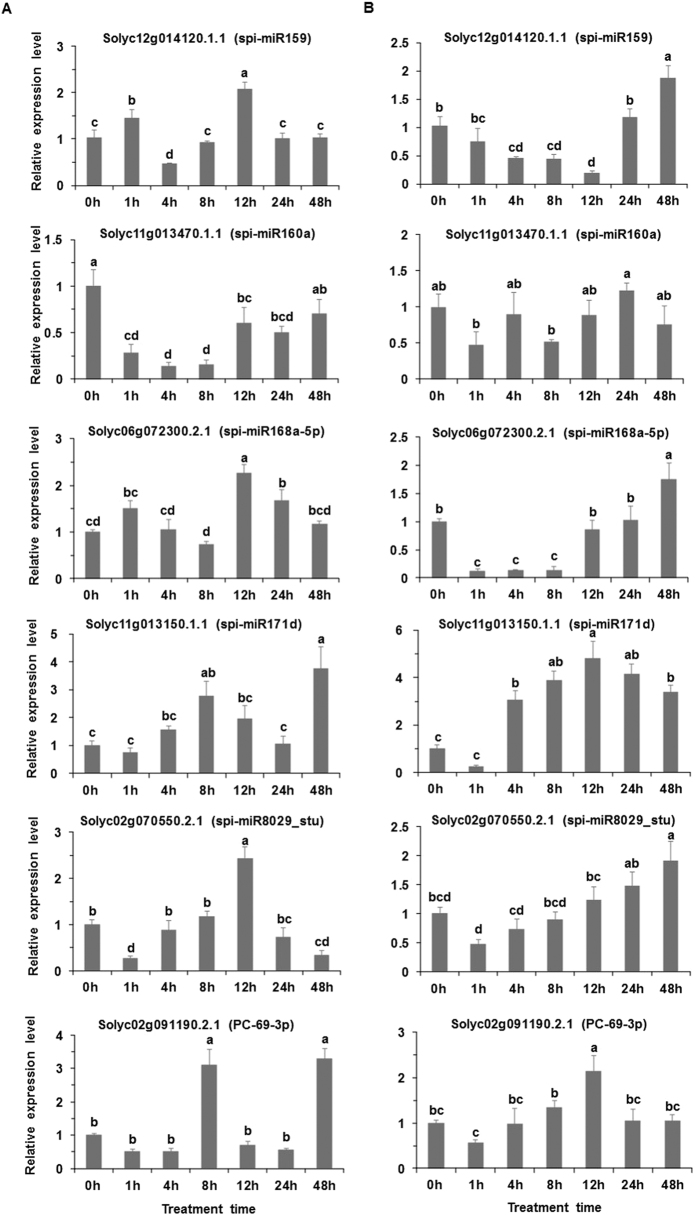
qRT-PCR validation of target genes in the wild tomato at control (0 h) or elevated temperature (1 h, 4 h, 8 h, 12 h, 24 h and 48 h). (**A**) Left, moderately elevated temperature (33/33 °C, MET); (**B**) right, acutely elevated temperature (40/40 °C, AET). The six sub-graphs in (**A**,**B**) give the values for six target genes. Each value was calculated as the mean of three samples ± standard error (SE). The different letters above the bar indicate significant difference (*P* < 0.05).

Compared to the control, the expression level of Solyc12g014120.1.1 cleaved by spi-miR159 significantly increased at 1 h and decreased at 4 h, with a significant increase at 12 h at the moderately elevated temperature; the expression level of Solyc11g013470.1.1 targeted by spi-miR160a significantly decreased at the moderately elevated temperature except at 48 h (Fig. 5A). Compared to the control, the expression level of Solyc06g072300.2.1 targeted by spi-miR168a-5p was significantly higher at 12 h and 24 h at the moderately elevated temperature in comparison with control (Fig. 5A). Compared to the control, the expression levels of Solyc11g013150.1.1 targeted by spi-miR171d and Solyc02g091190.2.1 targeted by PC-69-3p significantly increased at 8 h and 48 h at the moderately elevated temperature; the expression level of Solyc02g070550.2.1 cleaved by spi-miR8029_stu significantly decreased at 1 h and 48 h but increased at 12 h at the moderately elevated temperature (Fig. 5A). Compared to the control, the expression level of Solyc12g014120.1.1 was significantly lower at 4 h, 8 h and 12 h but higher at 48 h at the acutely elevated temperature; the expression level of Solyc11g013470.1.1 did not show significant difference at the acutely elevated temperature; the expression level of Solyc06g072300.2.1 significantly decreased at 1 h, 4 h and 8 h, with a significant increase at 48 h at the acutely elevated temperature (Fig. 5B). Compared to the control, the expression level of Solyc11g013150.1.1 significantly increased at 4 h, 8 h, 12 h, 24 h and 48 h at the acutely elevated temperature (Fig. 5B). Compared to the controls, the expression levels of Solyc02g070550.2.1 and Solyc02g091190.2.1were significantly higher at 48 h and 12 h, respectively, at the acutely elevated temperature (Fig. 5B).

## Discussion

### Conserved and novel miRNAs in tomato at the elevated temperatures

In our previous study, 67 tomato genotypes were screened in three levels of heat stress and the heat-sensitive and heat-tolerant tomato showed different physiological responses to heat stress^30^. High temperatures are less deleterious to the growth and development of the heat-tolerant tomato than those of the heat-sensitive tomato^29^,^30^. miRNAs, which are non-coding, play important roles in the regulation of plant growth and development and stress responses^33^,^34^,^35^. High-throughput sequencing has permitted research on heat-responsive miRNAs in *Triticum aestivum*^26^, *Brassica rapa*^36^,^37^, *Populus tomentos*a^38^ and *Saccharina japonica*^39^. However, it was still unknown how the heat-tolerant wild tomato (*Solanum pimpinellifolium* L.) responds to high temperature at the miRNA level and which genes targeted by the miRNAs are involved in the response to high temperatures in tomato plants. Additionally, it was unknown whether the response of miRNAs and their target genes differs between two different high temperatures. Therefore, we globally identified the heat-responsive miRNAs and their target genes under moderately and acutely elevated temperatures in a heat-tolerant wild tomato.

After screening and removal of redundant reads, 662 conserved miRNAs from 532 precursors and 97 novel miRNAs from 92 precursors were identified in this study (Supplementary Table S1), enriching the numbers of tomato miRNAs. Of the sRNAs identified in our study, the 24-nt sRNAs showed the highest abundance (Fig. 1), in agreement with the results from *Cucumis sativus*^20^ and *Raphanus sativus*^40^. A proportion of the conserved miRNAs (75.4%) and the novel miRNAs (93.8%) were shared among the three libraries (Fig. 2), suggesting that the majority of miRNAs are constitutively expressed at normal and high temperature conditions. The family analysis revealed that the miR166 family was the most populated family in the wild tomato plant, with 27 miRNAs (Fig. 3). Some miRNAs are conserved among some plant species, while others are species-specific^25^.

### Heat-responsive miRNAs in tomato at the elevated temperatures

There were 12 heat-responsive miRNAs identified in *Triticum aestivum*^26^. In *Brassica rapa*, five miRNA families were responsive to heat stress^37^. Wang *et al*.^36^ found that exposure to high temperatures decreased the abundance of chloroplast sRNAs (csRNAs) in *Brassica rapa* ssp. *Chinensis*. In *Populus tomentos*a, 52 heat-responsive miRNAs were identified^38^. Some miRNAs were up- or down-regulated when the plants were subjected to stress, suggesting that these miRNAs play crucial roles in plant stress tolerance^41^. In this study, the miRNAs that were significantly up- or down-regulated when the tomato plants were exposed to the elevated temperatures were considered to be heat-responsive. In summary, 96 and 150 miRNAs responded to the moderately and acutely elevated temperature, respectively (Supplementary Tables S3 and S4), which increased the number of heat-responsive miRNAs. This indicated that more miRNAs responded to the acutely elevated temperature than to the moderately elevated temperature in tomato. Most of the miRNAs (53/96) responding to the moderately elevated temperature were down-regulated, while most of the miRNAs (82/150) responding to the acutely elevated temperature were up-regulated. In *Populus euphratica*, most drought-responsive miRNAs were up-regulated^42^. In *Brachypodium distachyon* and *Solanum habrochaites,* most chilling-responsive miRNAs were down-regulated^13^,^25^. In *Populus tomentos*a, most heat-responsive miRNAs were down-regulated^38^. We inferred that the ratio of up-regulated miRNAs to down-regulated miRNAs was related to the plant species and the type of stress.

Moreover, some heat-responsive miRNAs were conserved, while other heat-responsive miRNAs were species-specific. On one hand, the changes in the expression levels of some heat-responsive miRNAs were consistent across species. For example, miR156 and miR167 were induced in *Triticum aestivum* and *Brassica rapa* by exposure to high temperatures (40 °C and 46 °C, respectively)^26^,^37^. In this study, spi-miR156f-5p_stu, spi-miR167a and spi-miR167b-3p_stu were significantly up-regulated (1.18, 1.19 and 1.65-fold, respectively) in the library from the plants held at 33 °C compared to the control library (Supplementary Table S3). By comparison, spi-miR167a was significantly up-regulated (1.05-fold) and spi-miR156b-p3 was detected only in the library from the plants held at 40 °C compared to the control library (Supplementary Table S3). These results indicate that expression of miR156 and miR167 in tomato plants is induced not only by exposure to acutely elevated temperatures (≥40 °C) but also by exposure to moderately elevated temperatures that were slightly higher than the optimum temperature for plant growth. miR398 and miR399 were inhibited in *Triticum aestivum* and *Brassica rapa* by exposure to high temperatures^26^,^37^. In this study, there were no significant changes in the expression levels of miR398 and miR399 in the 33 °C library compared to the control library (Supplementary Table S3). However, spi-miR398b-3p_stu targeting *CSD1* was significantly down-regulated (1.00-fold) in the 40 °C library compared to the control library and spi-miR399-p5 was not detected in the 40 °C library (Supplementary Tables S3 and S5). These indicated that miR398 and miR399 might only function at the acutely elevated temperatures.

On the other hand, some heat-responsive miRNAs might behave differently among species. For example, miR160 and miR168 were up-regulated in *Triticum aestivum* but down-regulated in *Populus tomentos*a by exposure to high temperatures^26^,^38^. In this study, miR160 showed no significant changes at the moderately elevated temperature, while spi-miR168a-5p_ath was significantly down-regulated (1.47-fold) (Supplementary Table S3). By comparison, spi-miR160a-3p_stu was detected only at the acutely elevated temperature, while spi-miR168a-3p was significantly down-regulated (1.07-fold) and spi-miR168a-3p_ath was significantly up-regulated (2.89-fold) under this condition (Supplementary Table S3). The different responses of the same miRNAs could be explained by differences in (1) heat-stress treatment in terms of duration and temperature and (2) plant species^39^. We found that some miRNAs (including miR156, miR167 and miR168) in tomato plants might respond when the plants are exposed to temperatures only slightly higher than the normal growing temperature, while others (miR160, miR398 and miR399) might only respond to acutely elevated temperatures.

### miRNAs play crucial roles in temperature stress by regulating the target genes in tomato

We found that 138 conserved miRNAs targeted 349 sequences (163 specific genes) and eight novel miRNAs targeted 13 sequences (10 specific genes) (Supplementary Tables S5 and S6). Most of the target genes of the conserved and novel miRNAs were not identified, possibly due to the low abundance of cleavage products or interactions between the miRNAs and their targets other than mRNA cleavage^25^.

In our previous study, the heat-tolerant wild tomato maintained higher chlorophyll content and net photosynthetic rate compared to the heat-sensitive tomato when exposed to seven days of heat stress^30^. *PyK*, which is targeted by spi-miR2275c-5p_ata and *RPE*, which is targeted by spi-miR3630-3p_vvi, play roles in carbon fixation in photosynthetic organisms. We suggest that miRNAs targeting the genes regulating carbon fixation in the heat-tolerant tomato might contribute to the maintenance of regular physiological activities in leaves at elevated temperatures, providing enhanced heat tolerance. The synthesis of Hsps was one plant response to the elevated temperatures^43^. Hsps are involved in the folding, assembly, translocation and degradation of protein and play crucial roles in plant responses to abiotic stresses by assisting in protein refolding^44^. The overexpression of *Hsp70* could improve the heat tolerance of *Arabidopsis thaliana*^45^. In this study, spi-miR6300_gma targeted *Hsp70* in the tomato plants at the moderately elevated temperature; spi-miR166c-3p and spi-miR166g-3p_osa targeted *Hsp60-3A* in the tomato plants at the acutely elevated temperature. This result suggests that these miRNAs could play important roles in plant responses to elevated temperatures by regulating *Hsps*. *AGO1* played crucial roles in the plant miRNAs production process^46^. The induction of *AGO1* in tomato under the elevated temperature as indicated by Fig. 5 could cause the change of other miRNAs abundance, which partly explains the reason why the variety and expression level of miRNAs changed in tomato under the elevated temperature. Moreover, *PHB* and other related classes of HD-ZIP transcriptional regulators played critical roles in promotion of vegetative growth in response to various signals^47^. MiR166 was down-regulated and thereby induced the expression of *PHB* when the heat-tolerant tomato received signals indicating elevated temperatures. We deduced that the regulation of *PHB* mediated by miR166 actively responded to the signal indicating elevated temperatures, which might increase heat tolerance in the tomato plants.

On one hand, some miRNAs (e.g., miR166 and miR408) showed consistent changes in expression levels in the tomato plants at the moderately and acutely elevated temperatures. On the other hand, certain miRNAs (e.g., miR171) exhibited the opposite changes at the moderately and acutely elevated temperatures, perhaps because certain miRNAs responded differently to different elevated temperatures. Moreover, certain novel miRNAs and their targets were involved in the plant’s response to the moderately elevated temperature (PC-52-5p, targeting *LRR-TPK*) and the acutely elevated temperature (PC-59-3p, targeting *MAPR3*), respectively. The qRT-PCR validation of expression levels of seven miRNAs and six target genes were generally consistent with the sequencing results, showing the accuracy of the sequencing result. Moreover, the expression level of the miRNAs and their targets showed opposite change at some time points, indicating that miRNAs played roles in tomato responding to the elevated temperature by regulating their target genes. For example, the expression level of spi-miR168a-5p decreased while the expression level of its target increased at 12 h and 24 h of the moderately elevated temperature and at 48 h of the acutely elevated temperature (Figs 4 and 5). Similarly, the expression level of spi-miR171d decreased while the expression level of its target increased at 8 h of the moderately elevated temperature and at 4 h, 8 h, 12 h, 24 h and 48 h of the acutely elevated temperature (Figs 4 and 5).

High- and low-temperature signals appear to be transduced by independent pathway components at the level of perception and signal transduction^3^. However, the metabolic and physiological responses to the high and low temperature were similar, indicating that some tolerance factors are common^3^. For example, the ROS scavengers and hormones were involved in the heat- and cold-stress responses in tomatoes^8^,^48^. In our previous research, we found that miR156, miR319, miR396 and miR398 were up-regulated and miR168 was down-regulated in a wild chill-tolerant tomato at low temperature^25^. In this study, we found that miR156 and miR396 were up-regulated and miR168 was down-regulated in the heat-tolerant wild tomato at the moderately elevated temperature. Moreover, miR319 and miR398 were down-regulated in the heat-tolerant wild tomato at the acutely elevated temperature. Therefore, both the low temperature and the moderately elevated temperature induced the expression of miR156 and miR396 and inhibited the expression of miR168 in the tomato plants. Meanwhile, the low temperature and acutely elevated temperature altered the production of miR319 and miR398, but in the opposite direction. Moreover, low temperatures caused the up-regulation of miR166 expression and the down-regulation of *PHB* expression in *Arabidopsis thaliana*^49^. Therefore, the regulation of *PHB* mediated by miR166 might have actively responded to both the heat and cold stress, but in the opposite direction. In summary, miRNAs actively respond to the high and low temperatures and play critical roles in improving temperature-stress tolerance in tomato plants by regulating target genes.

## Methods

### Plant materials, growth condition and sample collection

The heat-tolerant wild tomato LA2093 (*Solanum pimpinellifolium* L.), provided by Tomato Genetics Resource Center, University of California, was the plant material for sequencing. Single seeds were sown in a plug tray after soaking and pregermination and then cultured in climate chambers (RDN-560E-4, Dongnan Instrument Co, Ltd, Ningbo, China) at an air temperature of 26/18 °C (14 h/10 h, day/night), a light intensity of 280 ± 20 μmol m^−2^ s^−1^ and 50 ± 10% relative humidity. On the twentieth day after sowing, healthy and uniform seedlings were transferred to plastic plots (11 cm diameter, 9 cm height) and cultured in the climate chambers with the same environmental parameters. The seedlings were irrigated by flooding the bench for 10 min two times per day with 1/2 nutrient solution, according to the Japanese Garden test formula.

The seedlings were randomly divided into three groups for temperature treatments on the seventh day after transfer. The first group was kept at a normal temperature and used as a control (26/18 °C, NT); the second group was exposed to a moderately elevated temperature (33/33 °C, MET); and the third group was exposed to an acutely elevated temperature (40/40 °C, AET). The temperature reached 33 °C in half an hour and 40 °C in an hour in the climate chamber. According to the preliminary experiment, the soil moisture and electrical conductivity (EC) during the temperature treatments were in the normal ranges of 40–55% and 260–380 mS/m, as measured by WET Sensor (Delta-T Ltd, Burwell, England), when the seedlings were irrigated in the same way as before but with one more irrigation with water. Therefore, to keep the soil moisture and EC in a normal range and eliminate the effects of water deficit and drought stress, the experimental groups were subjected to an additional irrigation with water once per day during the temperature treatments.

The second fully expanded leaf was collected at 1 h, 4 h, 8 h, 12 h, 24 h and 48 h after treatment, immediately frozen in liquid nitrogen and stored at −80 °C. To decrease the effects of individual differences, leaves from five seedlings were mixed as one replicate, with three replicates per time point. Seedlings from which leaf samples had been taken were not used for further samples. The samples taken at 8 h were used for sequencing, and the samples taken at 1 h, 4 h, 8 h, 12 h, 24 h and 48 h were used for qRT-PCR analysis.

### sRNA libraries construction and high-throughput sequencing

Leaf samples from LA2093 exposed to 26/18 °C, 33/33 °C and 40/40 °C for 8 h were used to construct three sRNA libraries, which we called the NT, MET and AET libraries, respectively. To construct the three sRNA libraries, three leaf samples were subjected to sRNA extraction using TruSeq Small RNA Sample Preparation Kits (Illumina, San Diego, USA), respectively. Following isolation and ligation, the sRNAs were reverse transcribed to cDNAs. The cDNAs were used for PCR amplification and gel purification. The purified cDNAs from three sRNA libraries were sequenced with Illumina Hiseq2500 (LC-BIO, Hangzhou, China).

### sRNA data analysis and identification of conserved and novel miRNAs

After sequencing, raw reads were further analysed using the ACGT101-miR program (LC Sciences, Houston, Texas, USA). After removing junk reads and sequences <19 nt and >24 nt, remaining sequences were aligned using Rfam (http://rfam.janelia.org), *Solanaceae* Genome Network database (http://sgn.cornell.edu/) and repeat database (http://www.girinst.org/repbase). The sequences that matched Rfam, mRNA, rRNA, tRNA, snoRNA, snRNA, other non-coding RNAs and repeat sequences were filtered out. miRBase is the central online repository for miRNA data^50^. The valid sequences were analysed by blasting against the reported mature miRNAs and pre-miRNAs in plants from miRBase 21.0 (ftp://mirbase.org/pub/mirbase/CURRENT/). The valid sequences mapping to specific species mature miRNAs in hairpin arms were identified as conserved miRNAs. The valid sequences mapping to the other arm of known specific species precursor hairpin opposite to the annotated mature miRNA-containing arm were considered to be novel 5p- or 3p-derived miRNA candidates. A maximum of one mismatch between the target miRNAs and the known miRNAs from miRBase database was allowed. Secondary structures of the pre-miRNAs were predicted using RNAfold software (http://rna.tbi.univie.ac.at/cgi-bin/RNAfold.cgi)^14^, since a stable hairpin structure was an important indicator^51^. Only the pre-miRNAs with stable hairpin structures were considered. The miRNAs that are known among plants as compared with miRBase 21.0 and with stable hairpin structures were classified as conserved miRNAs^25^. For novel miRNAs, two more indexes were applied. Minimal folding free energy index (MFEI) is another important factor for miRNA identification^52^. Novel miRNAs were only considered when the MEFIs of their pre-miRNAs were ≥0.80 in this study. Moreover, the normalised copy number of the novel miRNAs was required to be ≥10 in at least one sample. The expression levels of the miRNAs were analyzed based on the sequences of mature miRNAs and the miRNAs with the same sequences shared the expression levels.

### Degradome sequencing

Total RNAs were extracted using Trizol reagent (Invitrogen, CA, USA) following the manufacturer’s procedure. The total RNAs quantity and purity were analysed using a Bioanalyser 2100 and an RNA 6000 Nano LabChip Kit (Agilent, CA, USA). All samples were assigned RNA integrity number (RIN) values of at least seven. Approximately 20 μg of total RNAs were used to construct the degradome libraries. The NT, MET and AET degradome libraries were constructed according to the methods described by Addo-Quaye *et al*.^23^ and Ma *et al*.^53^, with minor modifications. The mRNA was mixed with biotinylated random primers, and then the RNA containing the biotinylated random primers was captured by beads and ligated to 5′ adaptors. First-strand cDNA was generated from the ligated sequence after reverse transcription. A number of DNA products were produced by PCR amplification. Following purification, digestion, ligation and repurification, the cDNA library was sequenced with an Illumina Hiseq2500 (LC-BIO, Hangzhou, China). CleaveLand 3.0 software was used for data analysis after degradome sequencing^54^. With ftp.jgi-psf.org/pub/compgen/phytozome/.v10.0/Slycopersicum_225_iTAGv2.3/annotation/Slycopersicum_225_iTAGv2.3.transcript.fa.gz as reference data, the degradome sequences were matched to mRNAs using the Oligomap short reads aligner^55^. Target genes of the miRNAs were predicted by Targetfinder. A penalty score (alignment score) criterion was introduced based on the alignment between each miRNA and its potential target. Mismatched pairs were scored as 1, and G:U pairs were scored as 0.5. If the mismatches were located between the second base and the thirteenth base inclusive counting from the 5′ end of the miRNA sequence the score was doubled.

The targets were classified into five categories according to the results of target prediction and degradome sequencing and the abundance of the cleavage sequences. Category 0 miRNA-guided cleavage products were the most significant with only one maximum in target plot (Supplementary Figure S4). Category 1 comprised the most abundant products with more than one degradome tag in target plot. Category 2 products were present at abundances between the median and the maximum. Category 3 comprised the products with scores below or equal to the median. Category 4 sequences were only one raw read.

### Functional analysis of target genes

To clarify the functions of the target genes, GO functional annotation and KEGG pathway analysis of the miRNA targets were performed based on the GO database (http://www.geneontology.org/) and KEGG database (http://www.genome.jp/kegg/), respectively. The miRNAs with significantly different expression levels and their target genes were deprived according to the sRNAs sequencing and degradome sequencing, respectively. The functions of target genes were deprived according to the GO functional annotation.

### qRT-PCR validation of miRNAs and target genes

The samples taken at 1 h, 4 h, 8 h, 12 h, 24 h and 48 h were subjected to qRT-PCR. Total RNAs were extracted with Trizol reagent (Invitrogen, CA, USA), and the quantity and purity of the total RNA were detected using a Bioanalyser 2100 and RNA 6000 Nano LabChip Kit (Agilent, CA, USA). The samples that met a standard of quantity and purity were used for qRT-PCR validation of seven miRNAs and six target genes. The stem-loop RT primers, forward primers and reverse primers for miRNA qRT-PCR were designed according to the criteria described by Tang *et al*.^56^. The primers for mRNA qRT-PCR were designed using primer 5.0 (Primer-E Ltd., Plymouth, UK). The sequences of the primers for the miRNA and mRNA qRT-PCR are shown in Supplementary Tables S9 and S10, respectively.

Total RNAs were reverse transcribed into cDNA using the Prime Script RT reagent Kit (TaKaRa, Dalian, China) in an Eppendorf Mastercycler Gradient (Mastercycler^®^ep realplex, Hamburg, Germany). The reaction for reverse transcription were performed as follows: 42 °C for 60 min, 70 °C for 15 min and on ice for 3 min. The qRT-PCR amplifications were carried out with SYBR Premix Ex Taq^TM^, following the manufacturer’s instructions (Takara, Dalian, China) in an Eppendorf real-time PCR machine (Mastercycler^®^ep realplex, Hamburg, Germany). qRT-PCR reactions of miRNAs and target genes were performed as follows: 95 °C for 2 min, followed by 40 cycles of 95 °C for 15 s, 60 °C for 15 s and 72 °C for 20 s. Tomato U6 small nuclear RNA (U6snRNA) and actin were used as the reference genes for qRT-PCR of miRNAs and mRNAs, respectively. The reactions were repeated three times, and the expression levels were calculated by the 2^−ΔΔCt^ method.

### Statistical analysis

To compare the expression levels of the miRNAs or the target genes in tomatoes at normal temperature (NT), the moderately elevated temperature (MET) and the acutely elevated temperature (AET), a modified global normalization (TPM, tags per million) were used to correct copy numbers among different samples. A chi-square test and Fisher’s exact test were performed after the raw data were normalised from high-throughput sequencing. The log_2_ ratio was used as the threshold to detect differences in miRNA expression levels or the changes in target genes degradation. The miRNAs or target genes were considered to be significantly up-regulated or down-regulated when the *p* values of both the chi-square test and Fisher’s exact test were ≤0.05 and the |log_2_ ratio| was ≥1, according to our previous study^25^. qRT-PCR data were analyzed by analysis of variance (ANOVA) using SPSS 16.0 (SPSS Inc., Chicago, IL, USA). Based on miRNAs sequencing, target prediction and degradome sequencing, target genes predicted by Targetfinder and detected by degrdome sequencing with different expression levels cleaved by miRNAs with different expression levels were used for GO enrichment analysis. GO enrichment analysis was conducted with hypergeometric distribution (LC-BIO, Hangzhou, China). The formula for calculation is


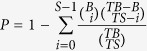


where TB was total number of mRNAs; TS was the number of mRNAs corresponding to selected miRNAs; B was the number of mRNAs annotated with specific functions; S was the number of mRNAs annotated with specific functions corresponding to selected miRNAs; *P* value ≤ 0.05 was regarded as the threshold.

## Additional Information

**Accession codes:** The sequencing data of this study has been submitted to Gene Expression Omnibus (GEO) under the accession number of GSE71819 at http://www.ncbi.nlm.nih.gov/geo/query/acc.cgi?token= wrmxwkiejxildkr&acc= GSE71819.

**How to cite this article**: Zhou, R. *et al*. Identification of miRNAs and their targets in wild tomato at moderately and acutely elevated temperatures by high-throughput sequencing and degradome analysis. *Sci. Rep.* **6**, 33777; doi: 10.1038/srep33777 (2016).

## Supplementary Material

Supplementary Information

Supplementary Table S1

Supplementary Table S2

Supplementary Table S3

Supplementary Table S4

Supplementary Table S5

Supplementary Table S6

## Figures and Tables

**Table 1:** Data of sRNA sequences in the NT, MET and AET library.

Types	NT library^A^				MET library^A^				AET library^A^			
	Total sRNA number	Percent of total (%)	Unique sRNA number	Percent of unique (%)	Total sRNA number	Percent of total (%)	Unique sRNA number	Percent of unique (%)	Total sRNA number	Percent of total (%)	Unique sRNA number	Percent of unique (%)
Raw reads	10,850,996	100.00	1,937,422	100.00	10,806,944	100.00	1,967,034	100.00	10,989,848	100.00	1,215,890	100
3ADT&length filter^B^	3,985,067	36.73	878,490	45.34	3,358,332	31.08	618,514	31.44	2,421,294	22.03	513,294	42.22
Junk reads	21,863	0.20	10,187	0.53	28,840	0.27	14,522	0.74	30,510	0.28	6,570	0.54
Rfam^C^	720,517	6.64	50,539	2.61	580,535	5.37	40,566	2.06	675,042	6.14	34,628	2.85
mRNA	2,989,464	27.55	183,508	9.47	3,031,582	28.05	210,920	10.72	3,564,171	32.43	112,495	9.25
Repeats^D^	9,757	0.09	774	0.04	9,021	0.08	827	0.04	5,887	0.05	476	0.04
rRNA	568,456	5.24	34,268	0.32	444,594	4.11	26,649	0.25	514,197	4.68	23,682	0.22
tRNA	111,052	1.02	9,357	0.09	104,212	0.96	8,066	0.07	128,180	1.17	6,867	0.06
snoRNA	3,106	0.03	925	0.01	2,952	0.03	853	0.01	2,748	0.03	547	0
snRNA	2,354	0.02	1,237	0.01	2,680	0.02	1,213	0.01	3,181	0.03	741	0.01
other Rfam RNA	35,549	0.33	4,752	0.04	26,097	0.24	3,785	0.04	26,736	0.24	2,791	0.03
Valid reads	3,333,687	30.72	829,756	42.83	3,964,329	36.68	1,095,508	55.69	4,483,258	40.79	560,147	46.07

^A^NT, control at normal temperature; MET, heat stress at moderately elevated temperature; AET, heat stress at acutely elevated temperature.

^B^Reads lacking three ADTs or with lengths <19 nt or >24 nt were removed.

^C^Collection of many common noncoding RNA families other than micro RNAs; http://rfam.janelia.org.

^D^Downloaded from http://www.girinst.org/repbase.

**Table 2:** Data summary identified in the NT, MET and AET library by degradome sequencing.

Libraries		Raw reads	Unique raw reads	cDNA mapped reads	Unique cDNA mapped reads	Number of input cDNAs	Number of coverd cDNAs
NT	Number	11,774,232	3,596,303	9,441,429	2,537,930	34,727	22,865
	Ratio	—	—	80.19%	70.57%	—	65.84%
MET	Number	11,471,728	3,379,825	9,033,761	2,345,027	34,727	22,889
	Ratio	—	—	78.75%	69.38%	—	65.91%
AET	Number	10,853,424	3,454,547	8,779,928	2,427,370	34,727	22,963
	Ratio	—	—	80.90%	70.27%	—	66.12%

NT, control at normal temperature; MET, treatment at moderately elevated temperature; AET, treatment at acutely elevated temperature.
