# Supplementary material for: Identification of miRNAs and their targets in wild tomato at moderately and acutely elevated temperatures by high-throughput sequencing and degradome analysis
Source: Sci Rep. 2016 Sep 22;6:33777. doi: 10.1038/srep33777 (PMC5031959; doi:10.1038/srep33777)
Supplement: Supplementary Information [file srep33777-s1.pdf]

Supplementary Information in pdf format of the paper titled ‘Identification of miRNAs and their targets in wild tomato at moderately and acutely elevated temperatures by high-throughput sequencing and degradome analysis’ by Rong Zhou, Qian Wang, Fangling Jiang, Xue Cao, Mintao Sun, Min Liu and Zhen Wu.

Supplementary Information of the paper titled 'Identification of miRNAs and their targets in wild tomato at moderately and acutely elevated temperatures by high-throughput sequencing and degradome analysis' by Rong Zhou, Qian Wang, Fangling Jiang, Xue Cao, Mintao Sun, Min Liu and Zhen Wu.

Supplementary Figure S1. Folding structures of novel miRNAs in tomato LA2093 at a control temperature (26/18°C, NT), moderately elevated temperature (33/33°C, MET) and acutely elevated temperature (40/40°C, AET)

Supplementary Figure S2. GO functional annotation of target genes cleaved by conserved miRNAs in tomato LA2093

Supplementary Figure S3. GO enrichment analysis of the target genes that predicted by Targetfinder and detected by degrdome sequencing with different expression levels cleaved by miRNAs with different expression levels in tomato LA2093. **(A)** MET vs NT, **(B)** AET vs NT. The x-axis indicated rich factor; the y-axis indicated GO term. The size of the circle indicated the number of genes with the functions; the different colours of the circle indicated different significance levels.

Supplementary Figure S4. Target plots of some mRNAs from category 0 in tomato LA2093 at **(A)** a control temperature (26/18°C, NT), **(B)** moderately elevated temperature (33/33°C, MET) and **(C)** acutely elevated temperature (40/40°C, AET)

Output of sir_graph ( )
by D. Stewart and M. Zuker

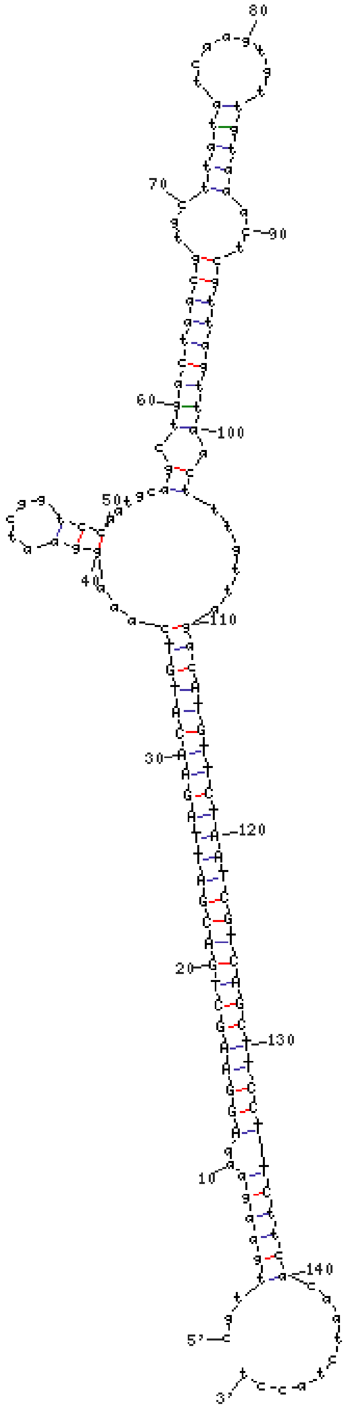

dG = -66.7 PC-1

PC-1

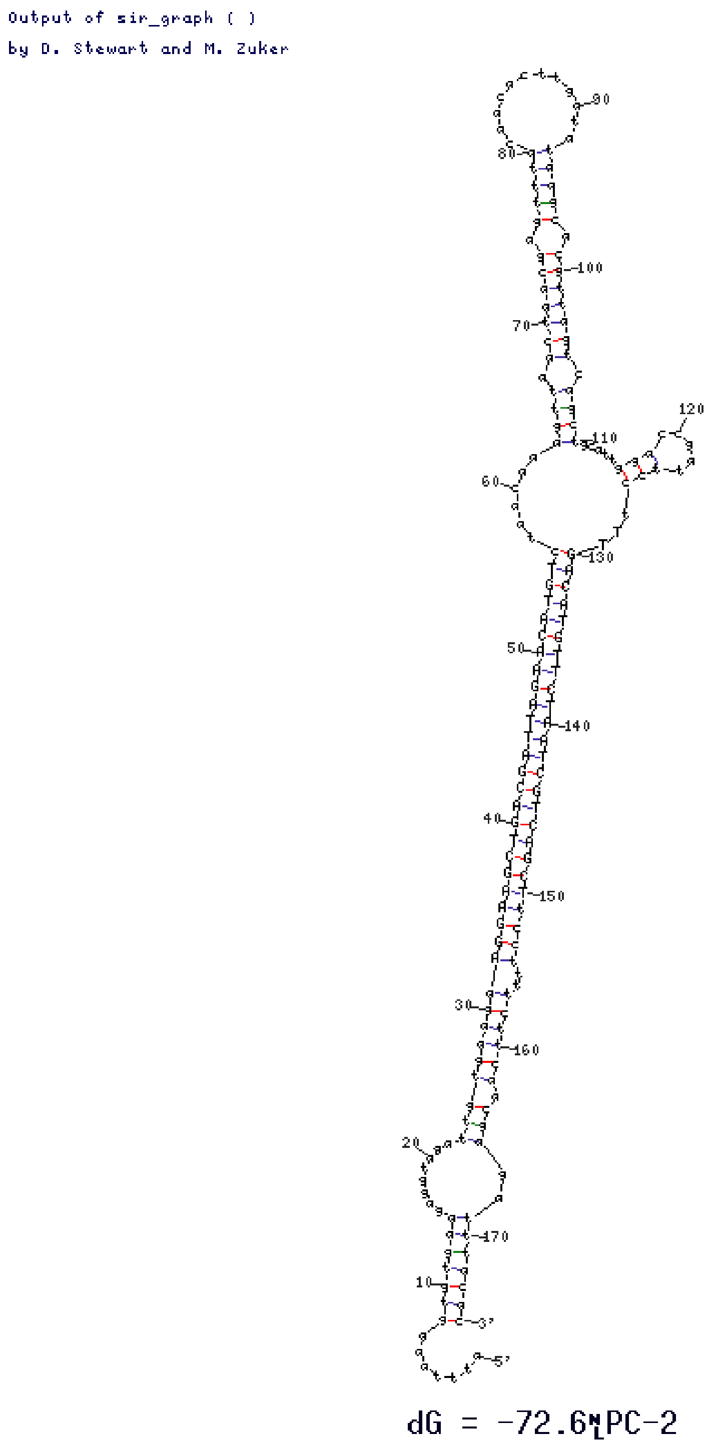
Output of sir_graph ( )
by D. Stewart and M. Zuker
10
20
30
40
50
60
70
80
90
100
110
120
130
140
150
160
170
3'
5'
dG = -72.6
PC-2

PC-2

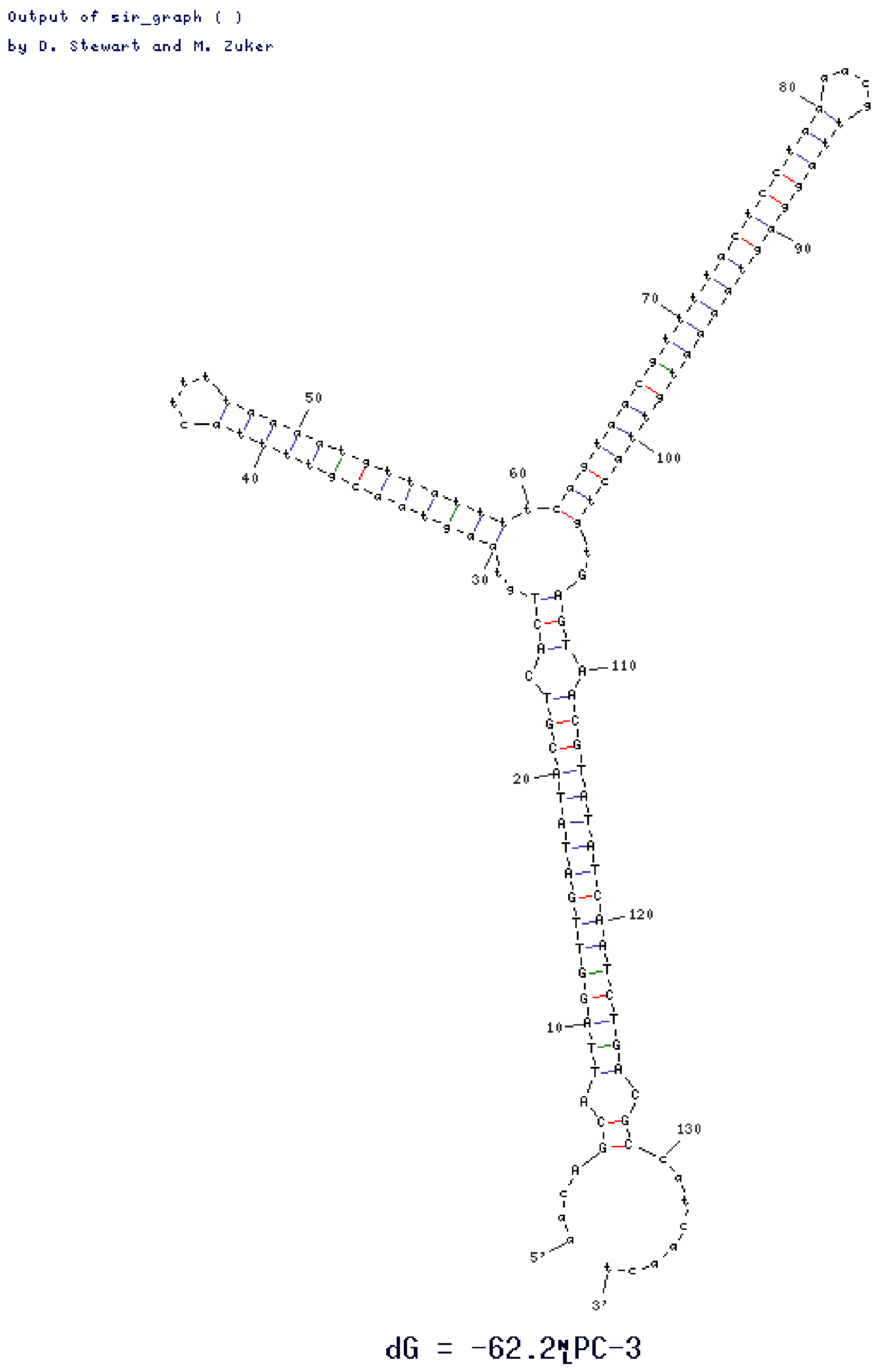

PC-3

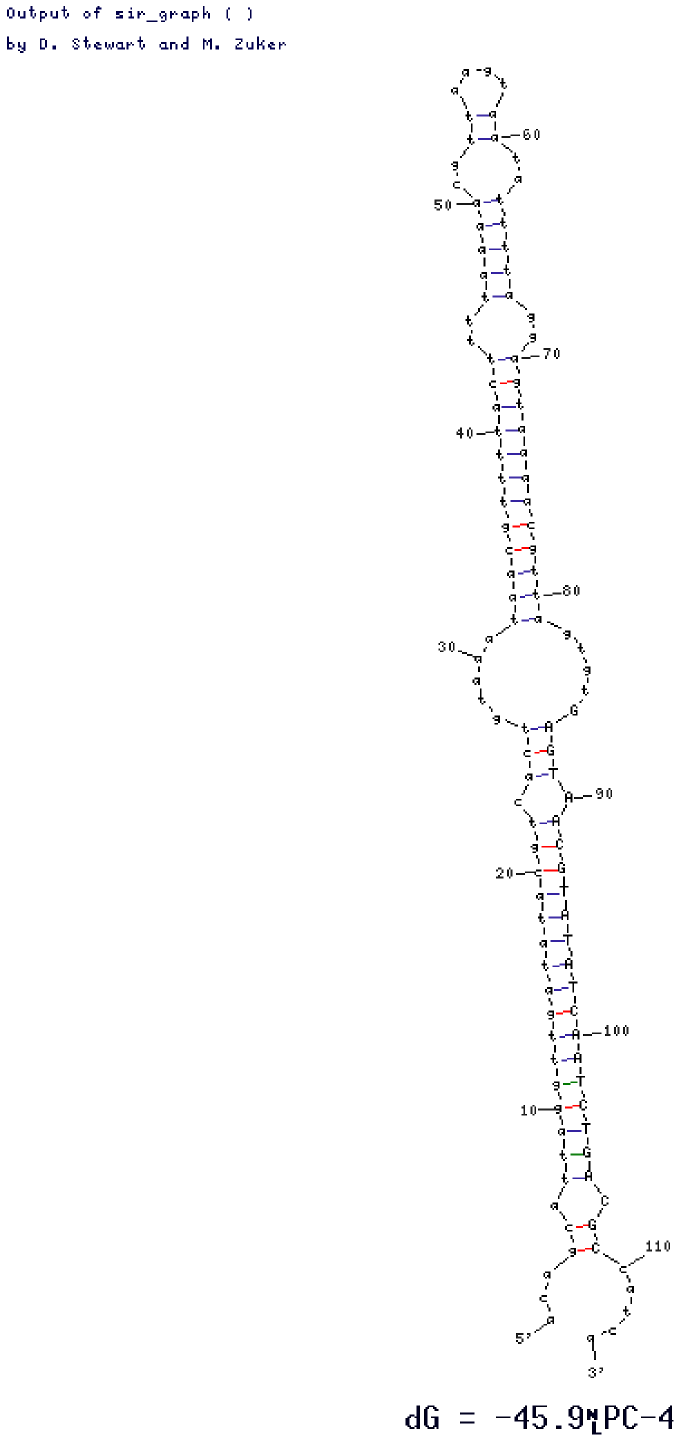
Output of sir_graph ( )
by D. Stewart and M. Zuker
60
50
70
40
80
30
90
20
100
10
110
5'
3'
dG = -45.9 PC-4

PC-4

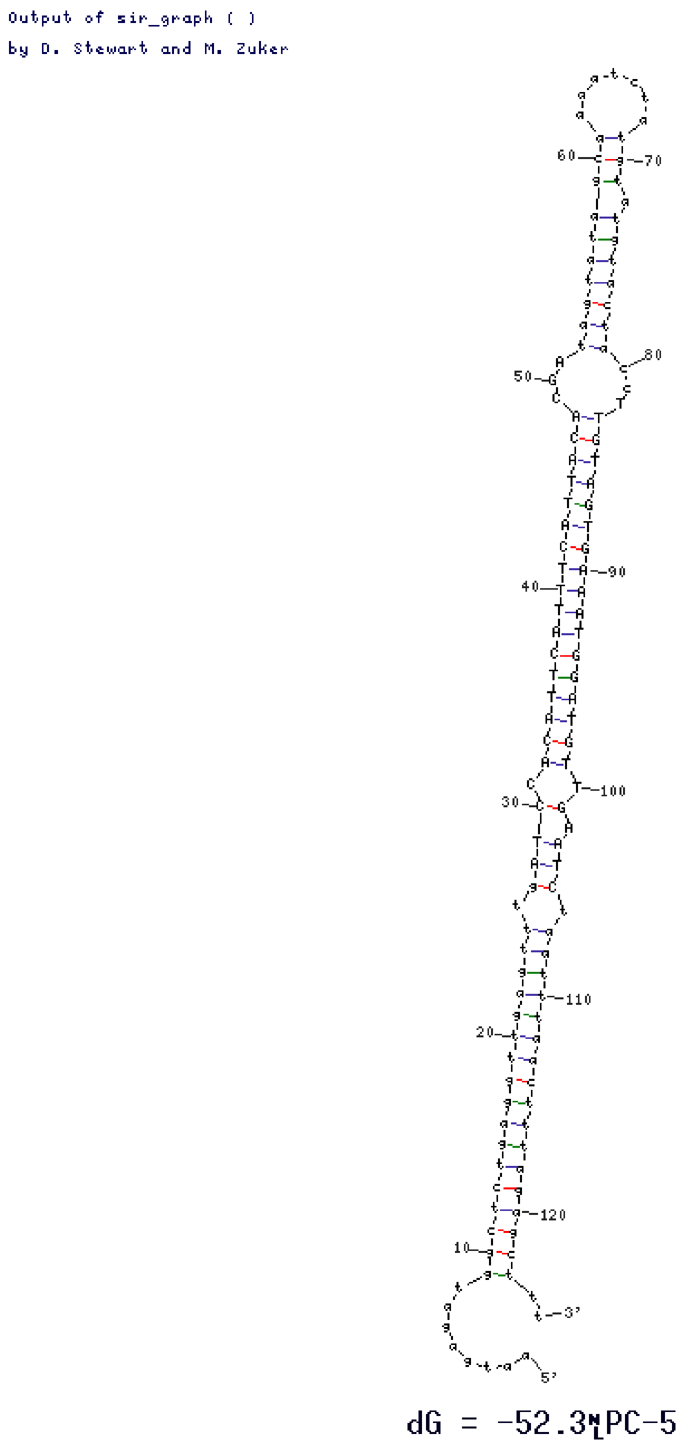

PC-5

Output of sir_graph ( )
by D. Stewart and M. Zuker

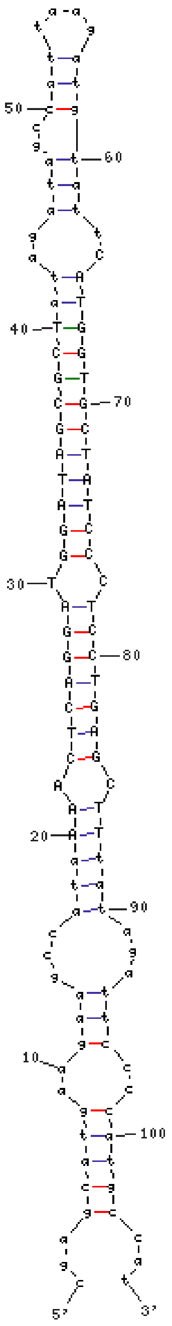

dG = -45[illegible]PC-6

PC-6

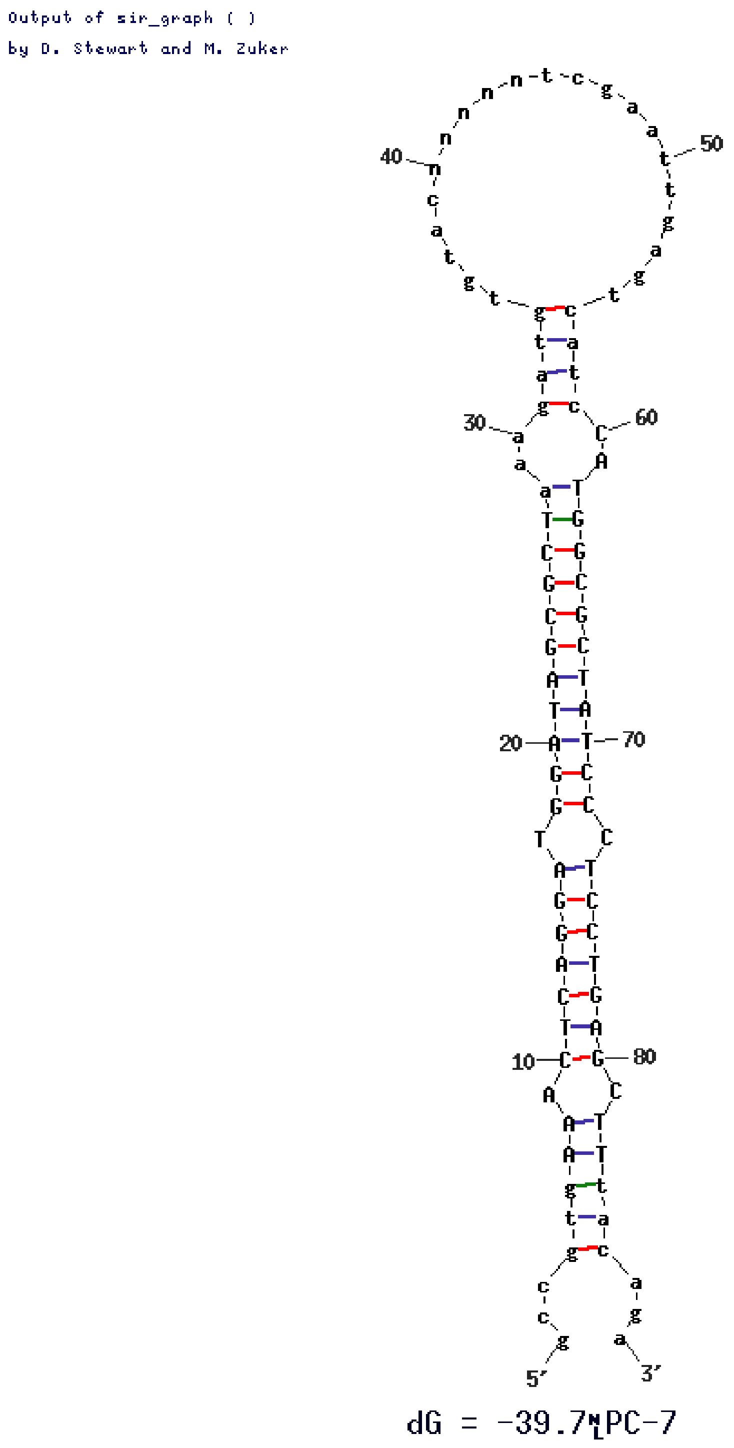
Output of sir_graph ( )
by D. Stewart and M. Zuker
10
20
30
40
50
60
70
80
5'
3'
dG = -39.7
PC-7

PC-7

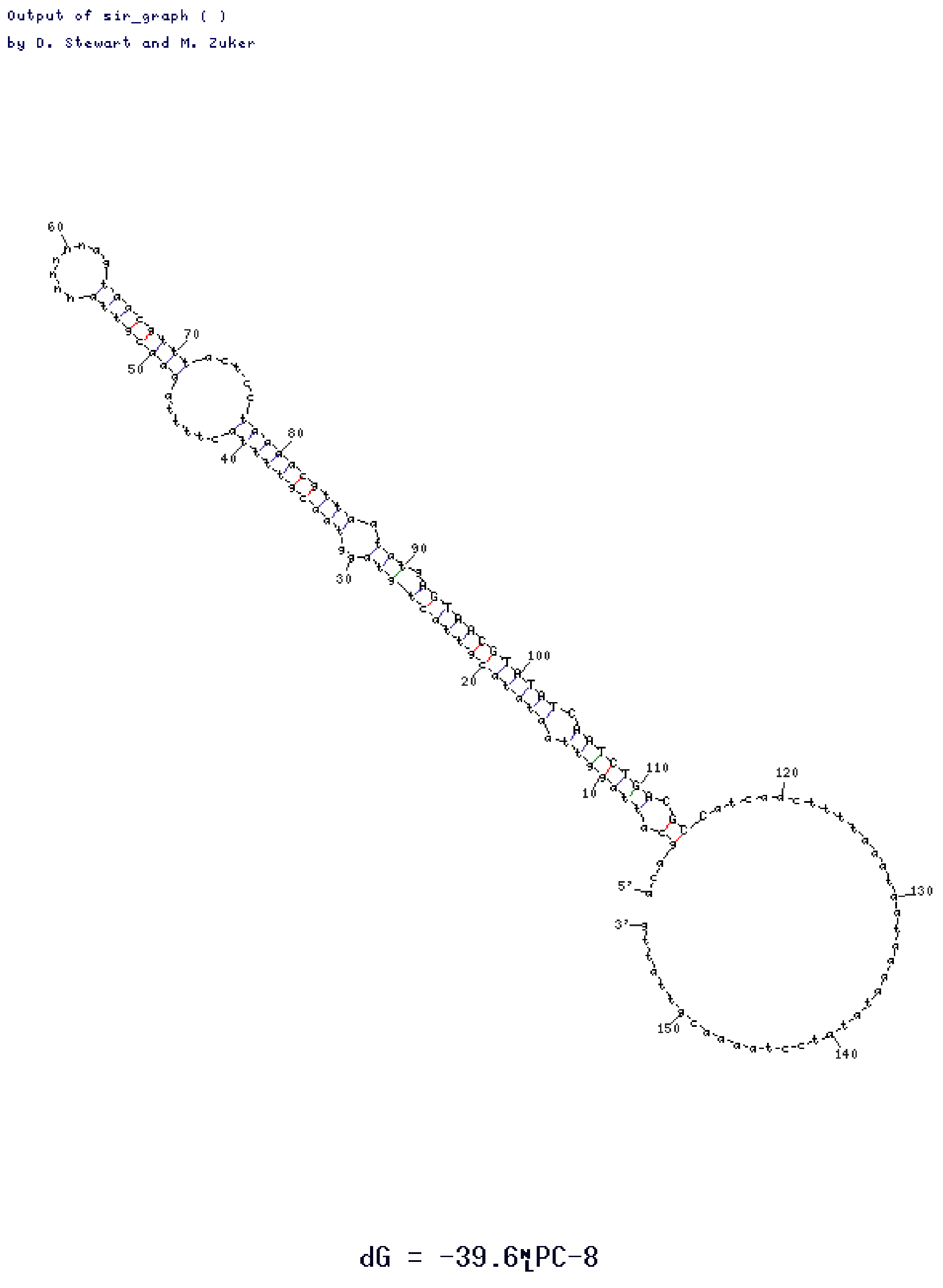
Output of sir_graph ( )
by D. Stewart and M. Zuker
60
70
50
80
40
90
30
100
20
110
10
120
5'
130
3'
150
140
dG = -39.6[illegible]PC-8

PC-8

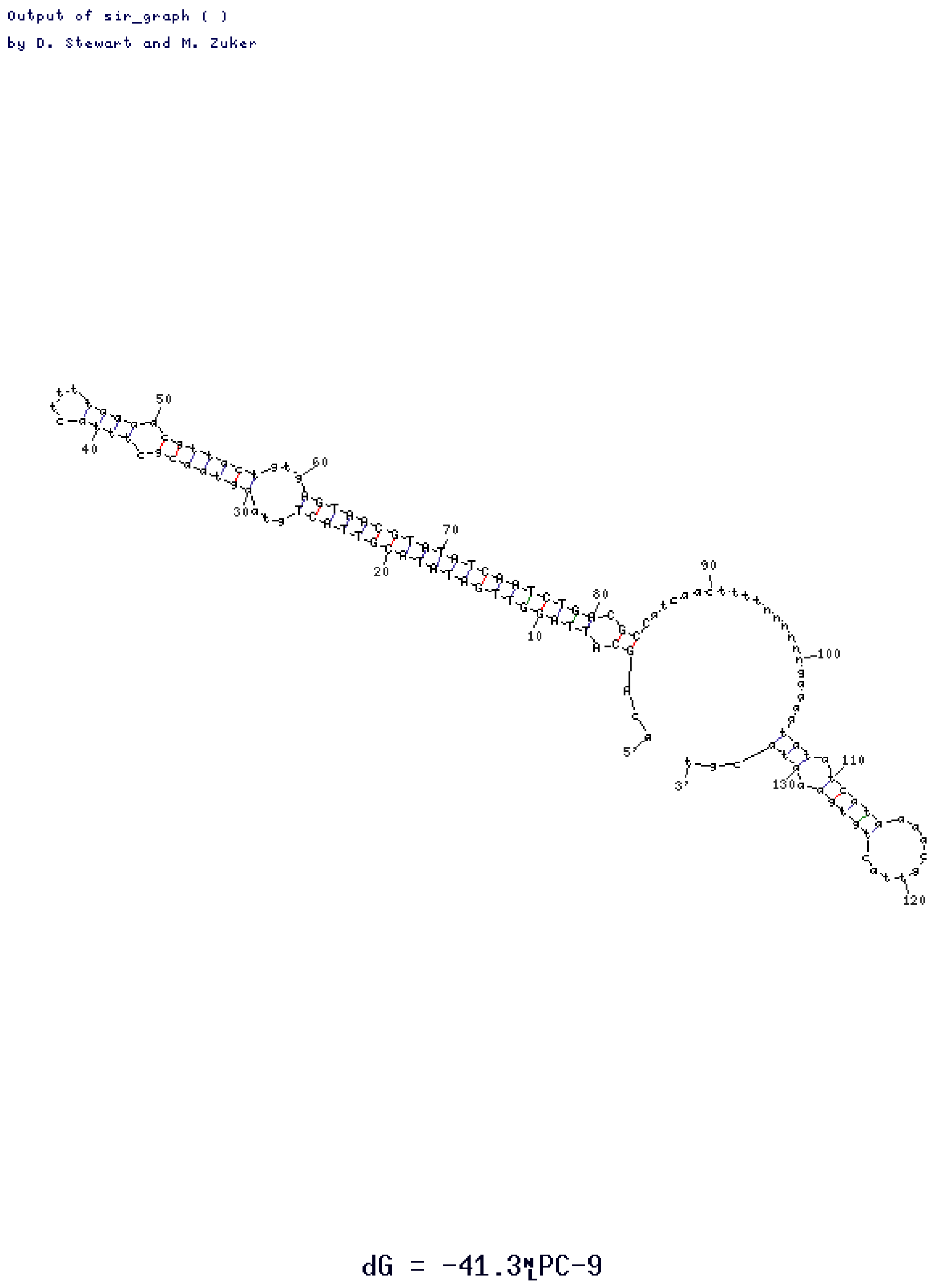

PC-9

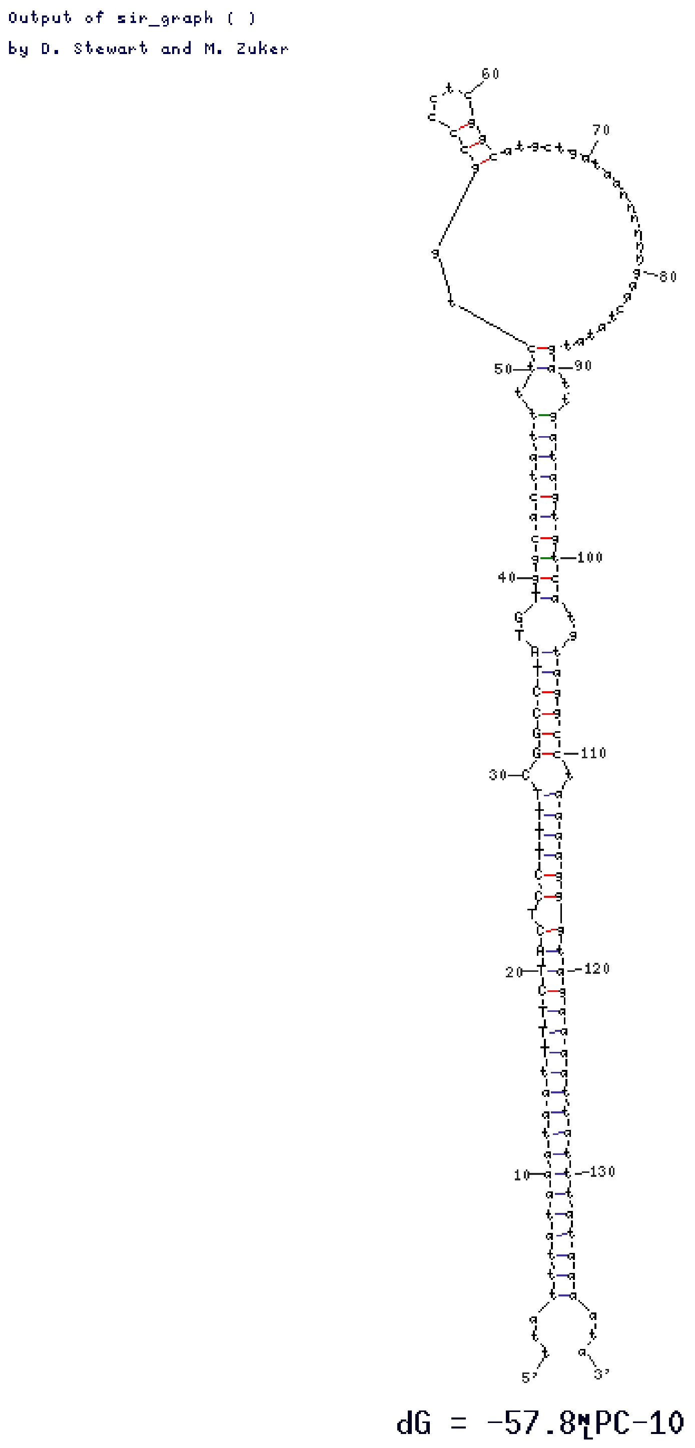

PC-10

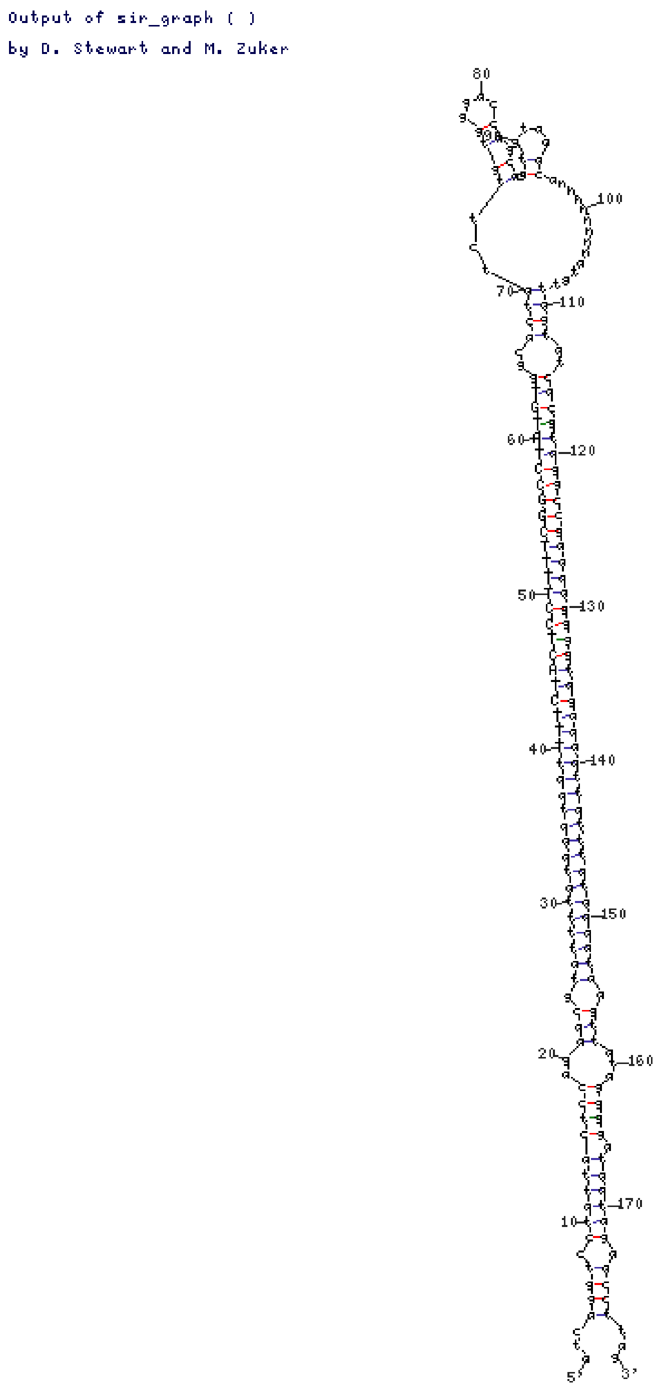

dG = -83.1 PC-11

PC-11

Output of sir_graph ( )
by D. Stewart and M. Zuker

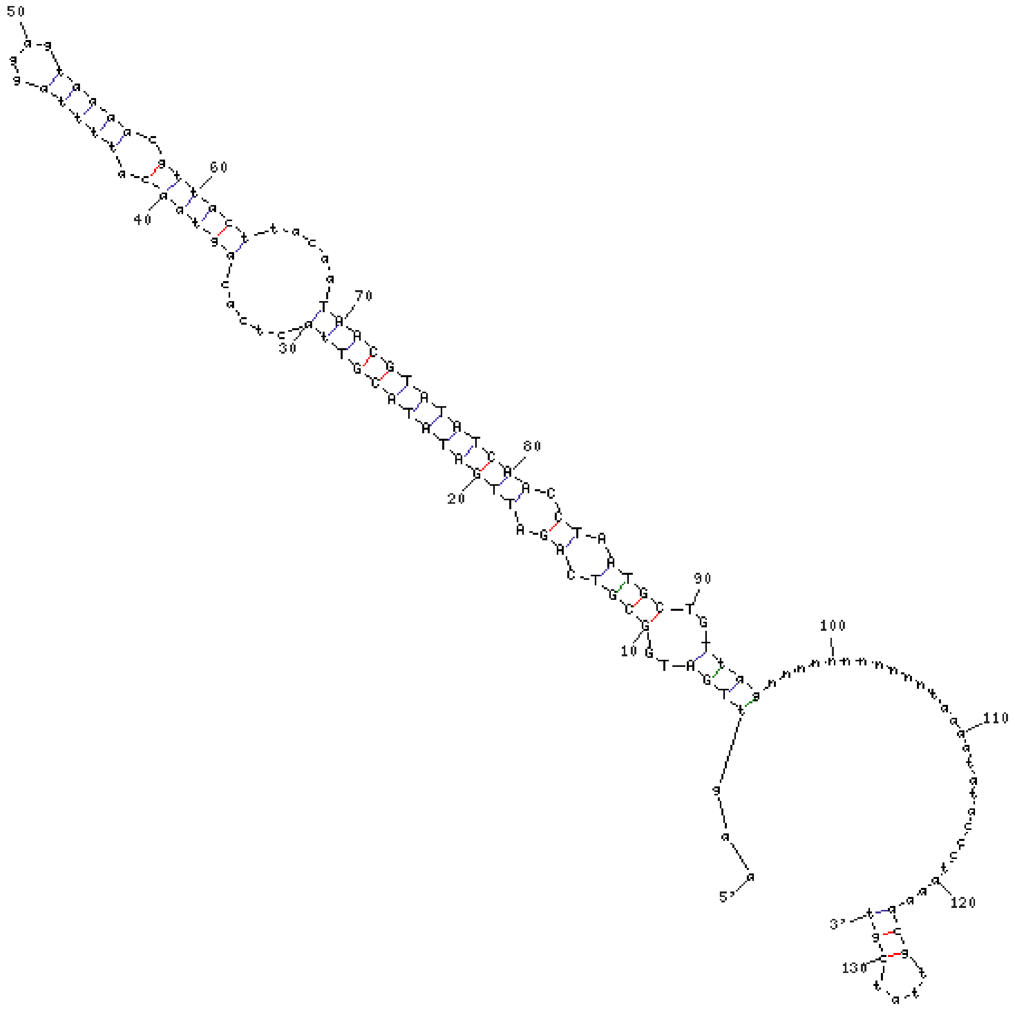

dG = −33[illegible]PC−12

PC-12

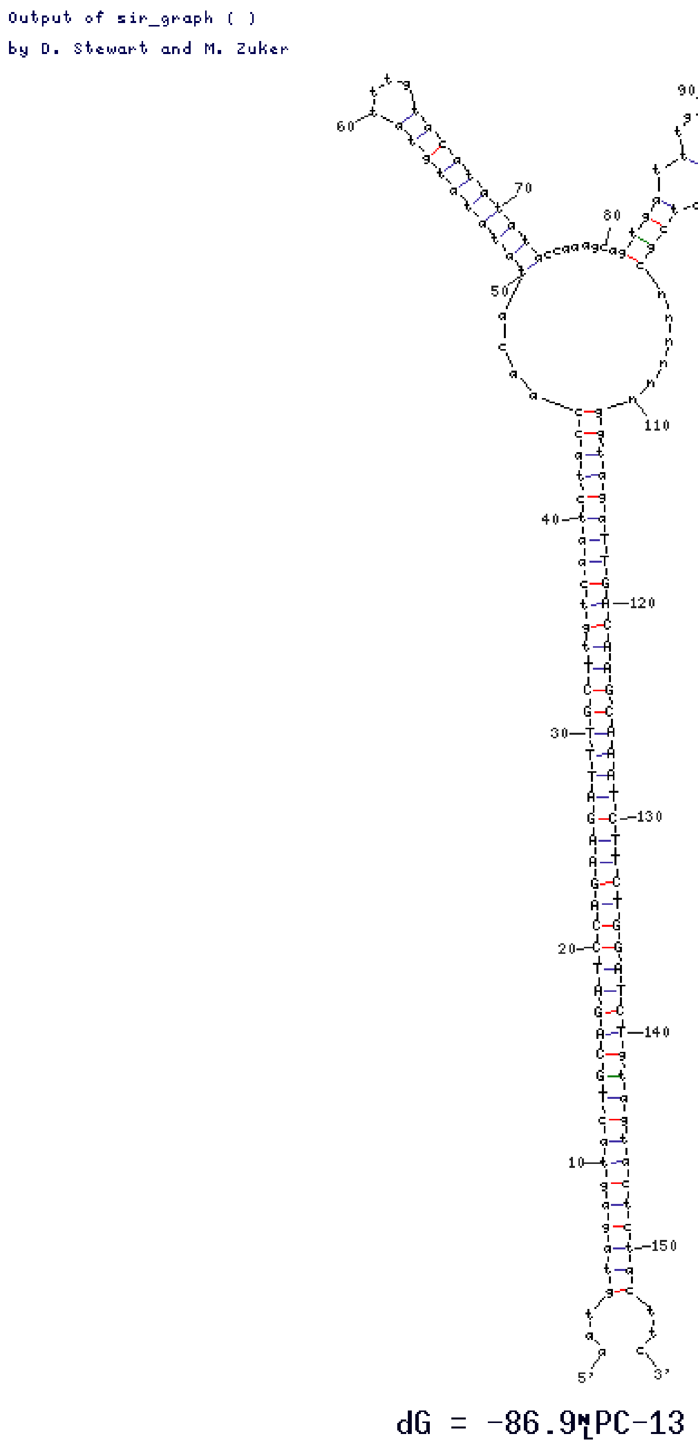

PC-13

Output of sir_graph ( )
by D. Stewart and M. Zuker

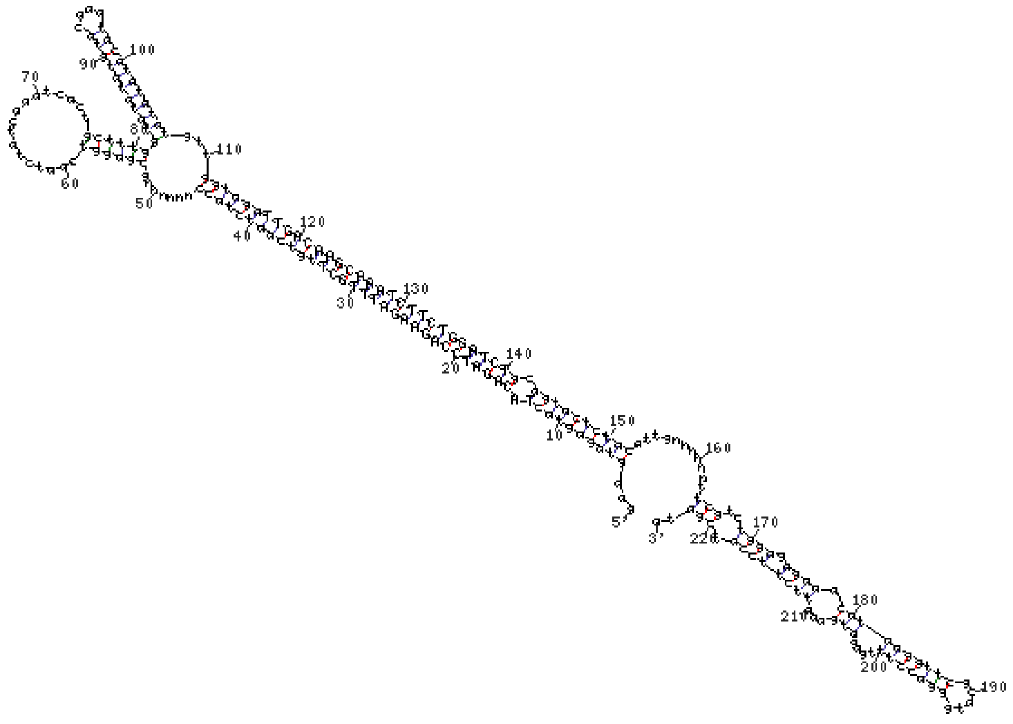

dG = -98.1 PC-14

PC-14

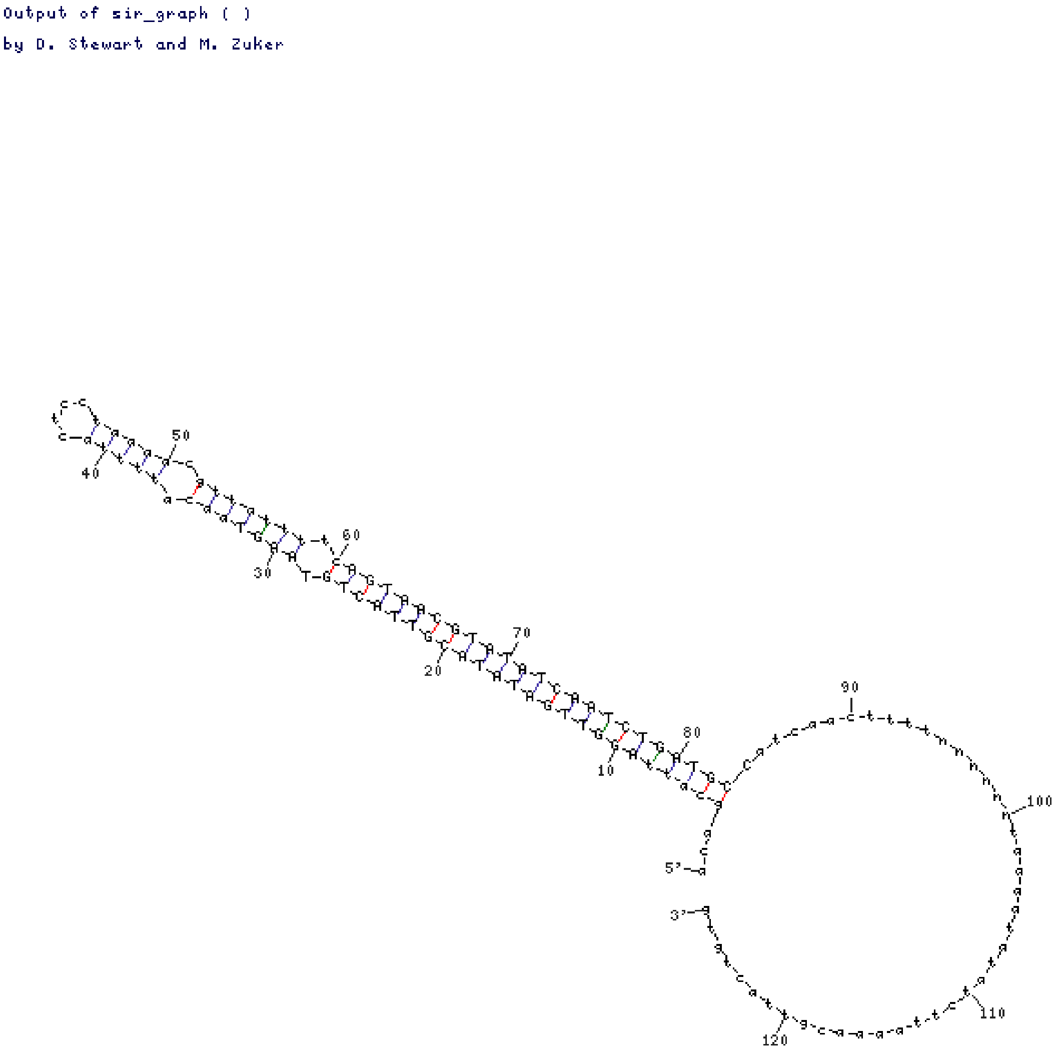

dG = -44.2 PC-15

PC-15

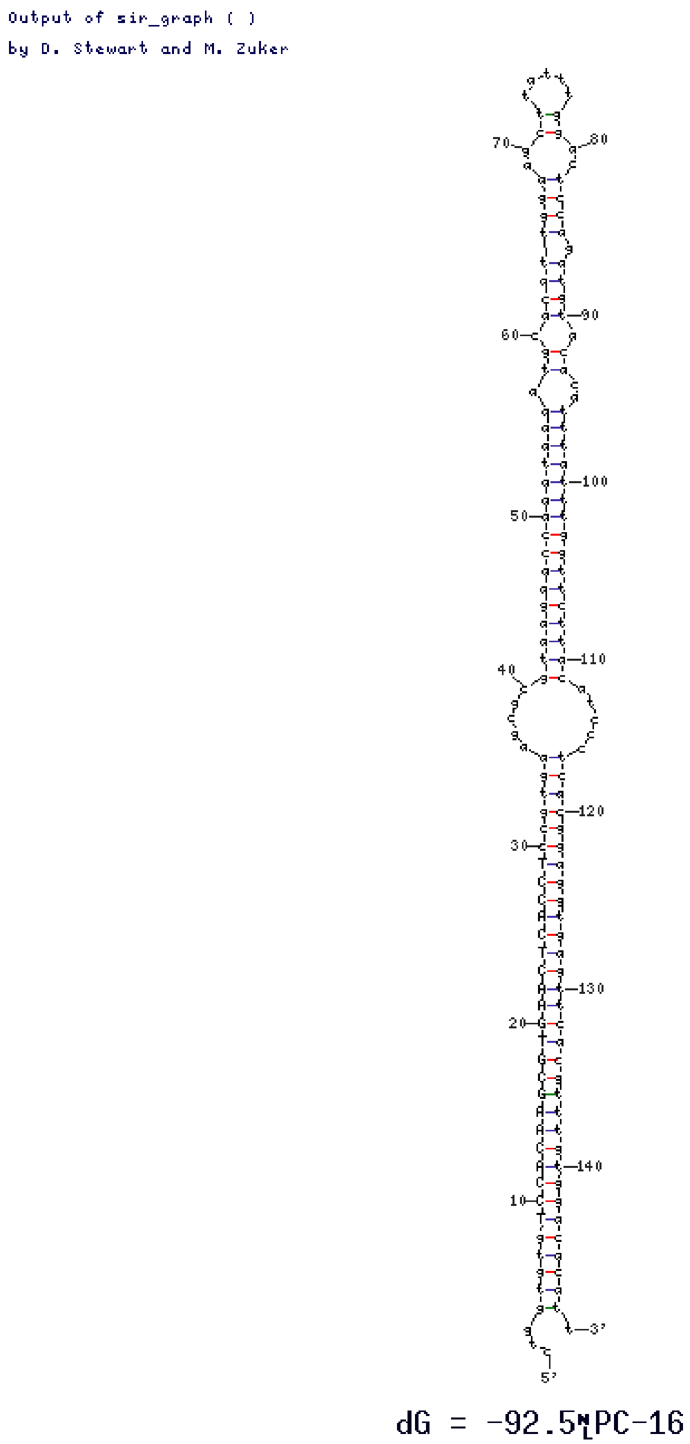

PC-16

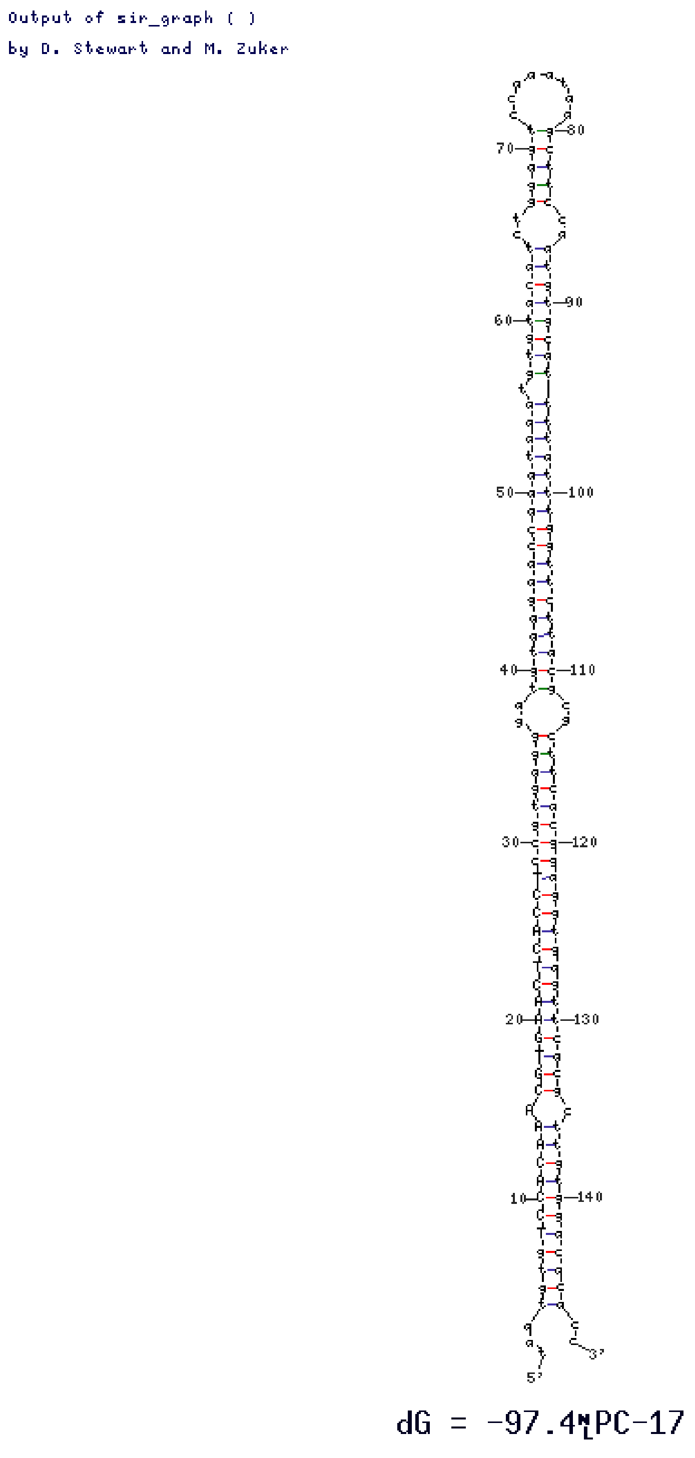

PC-17

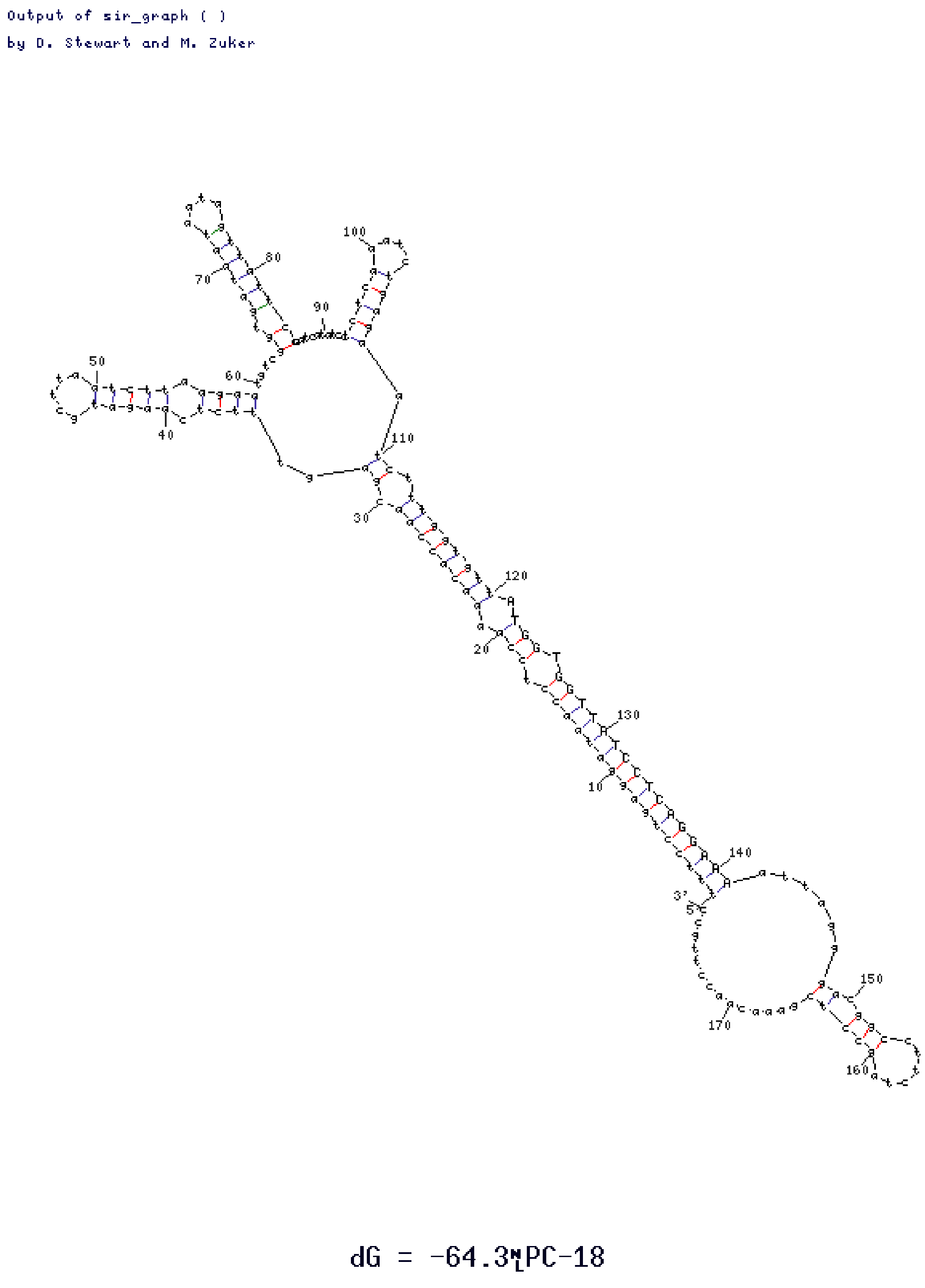

PC-18

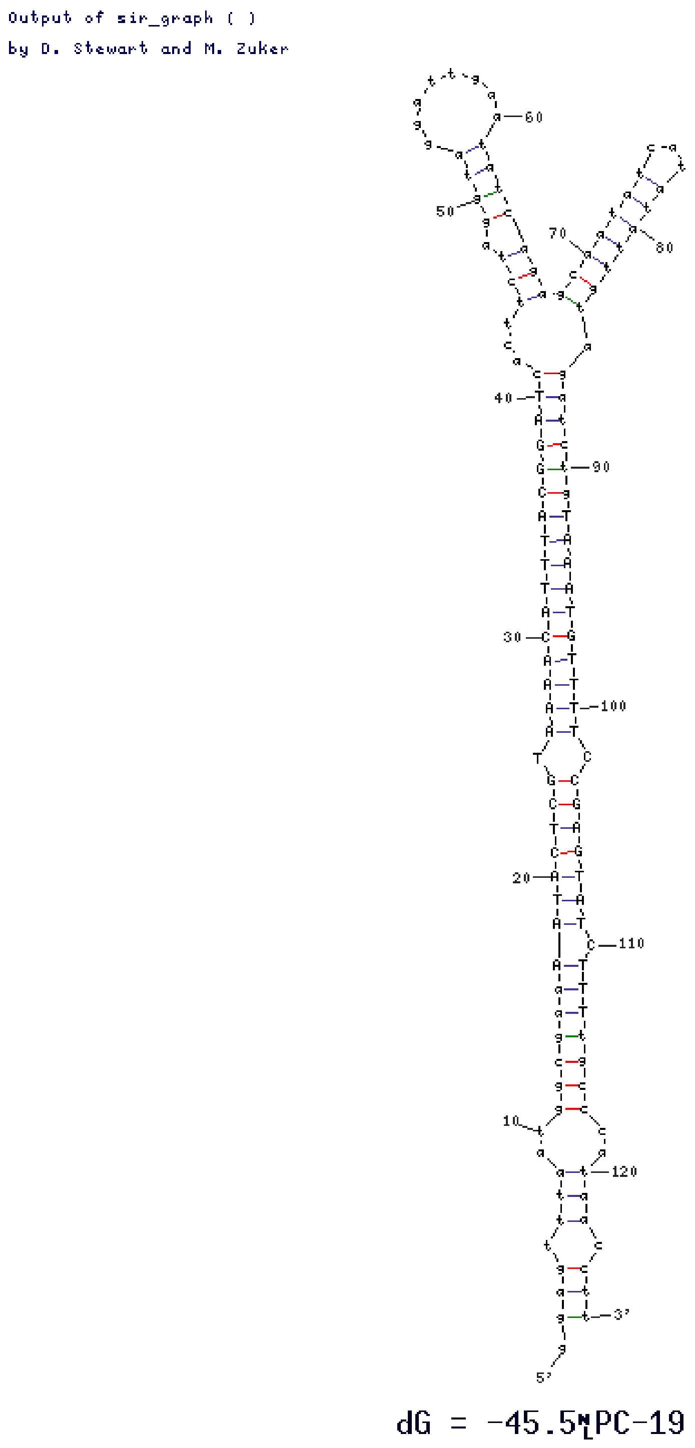

PC-19

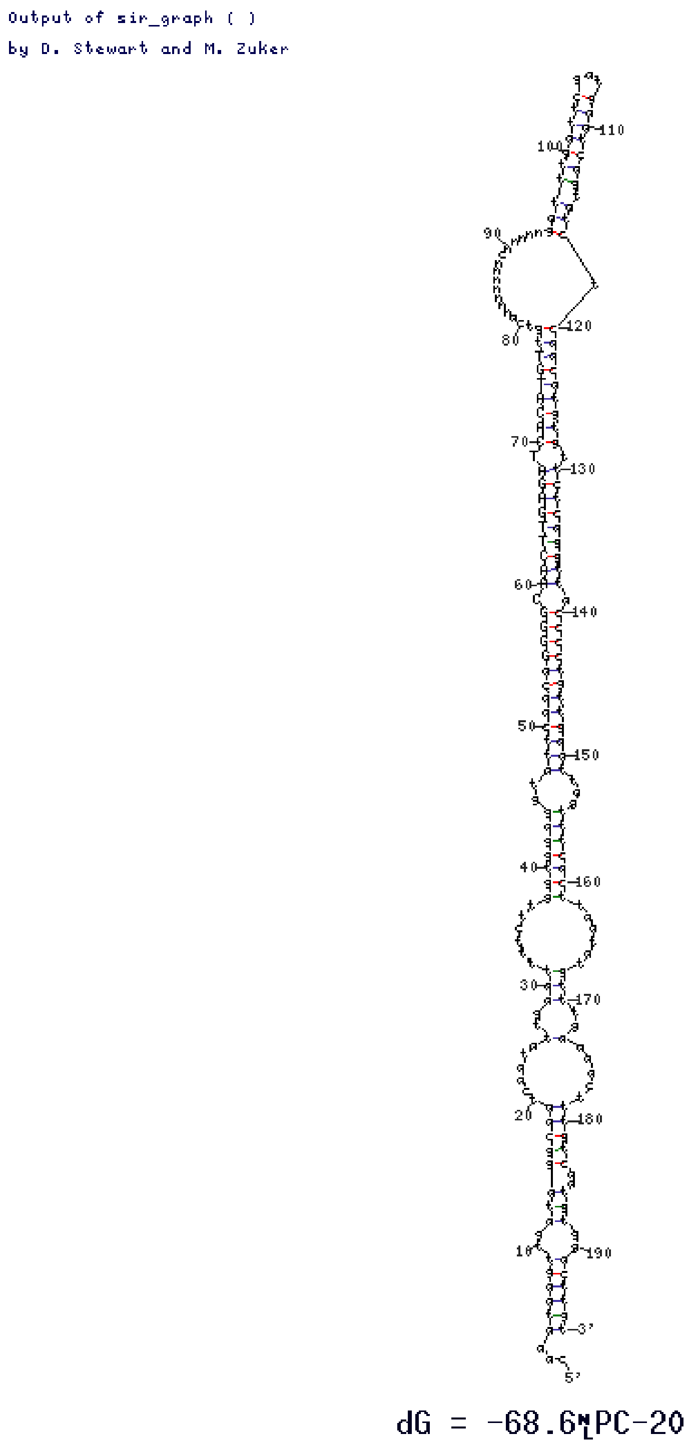

PC-20

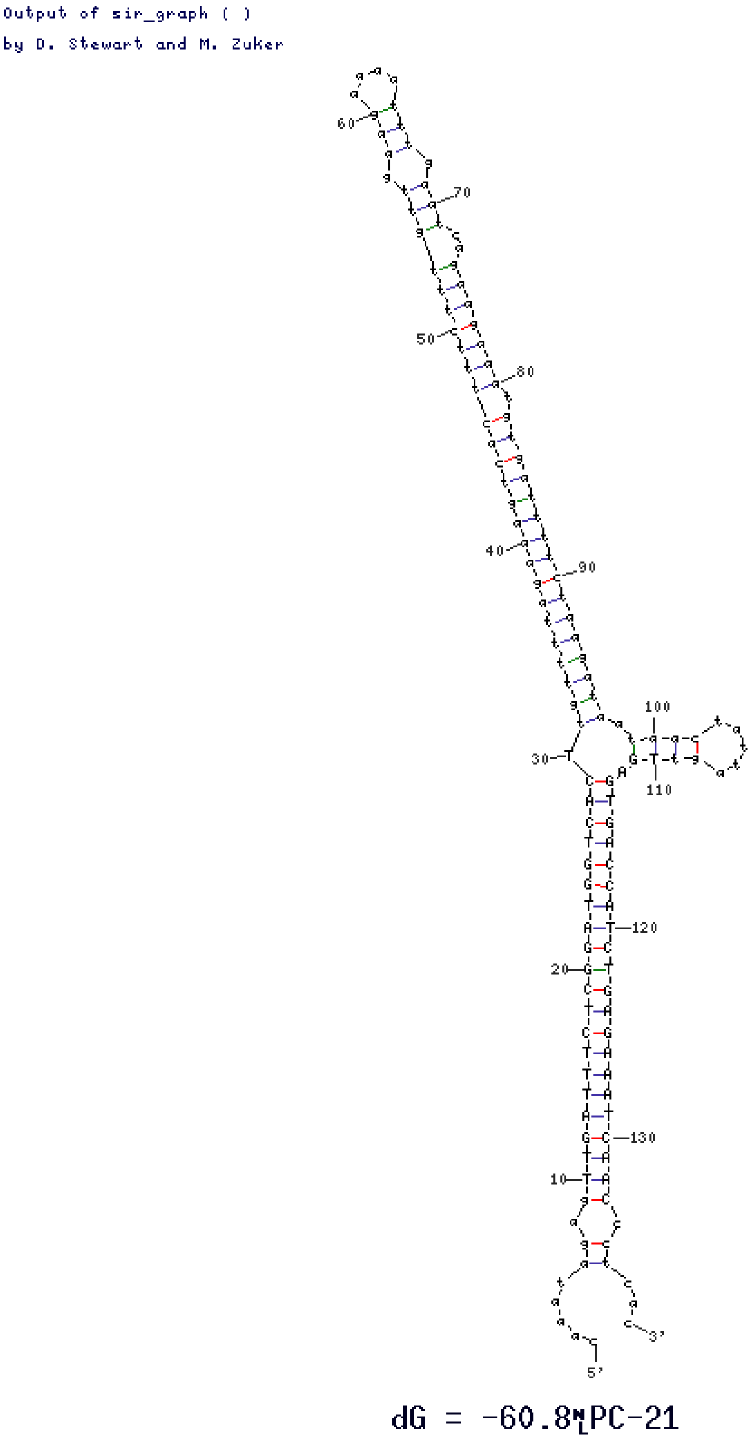

PC-21

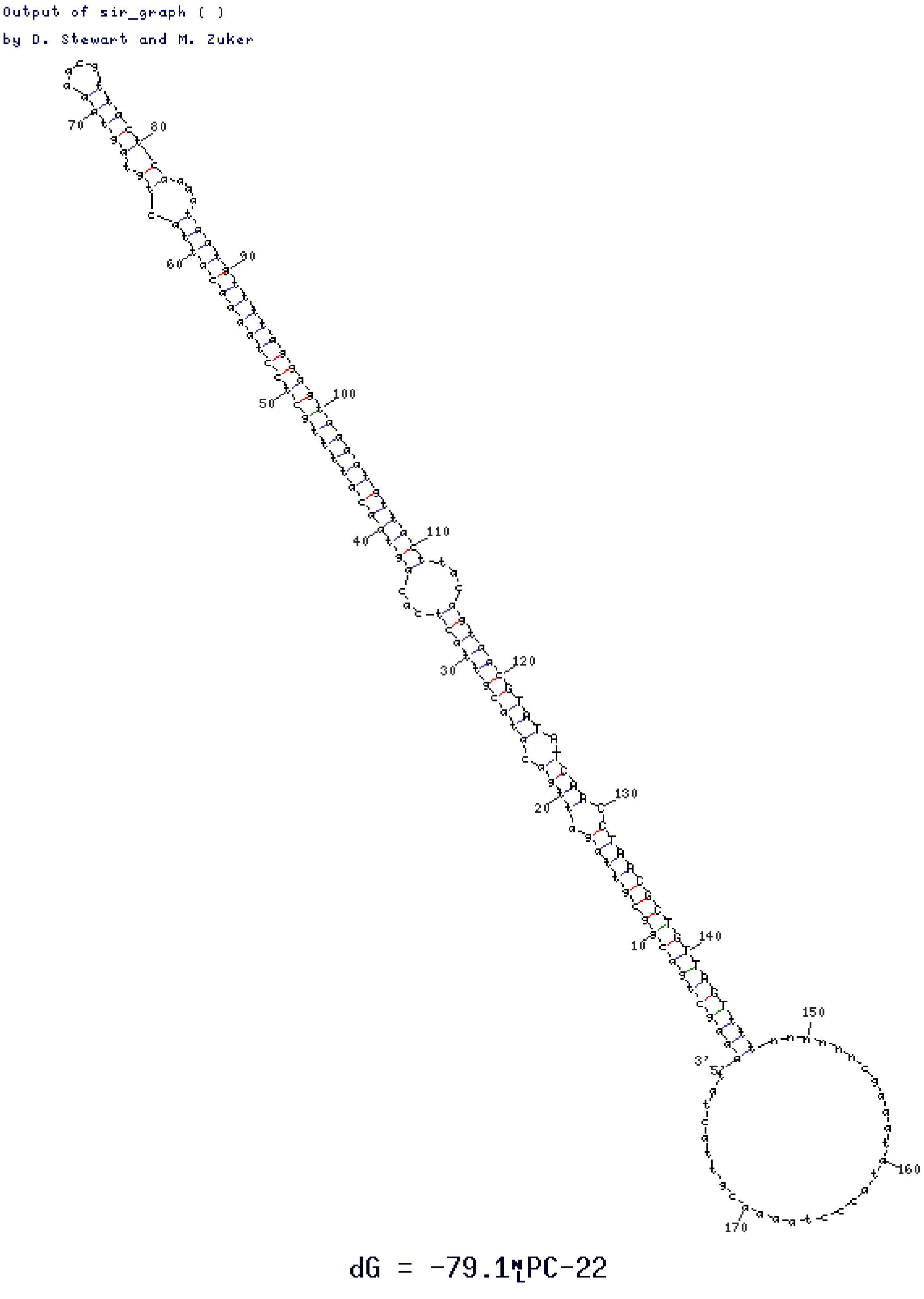

PC-22

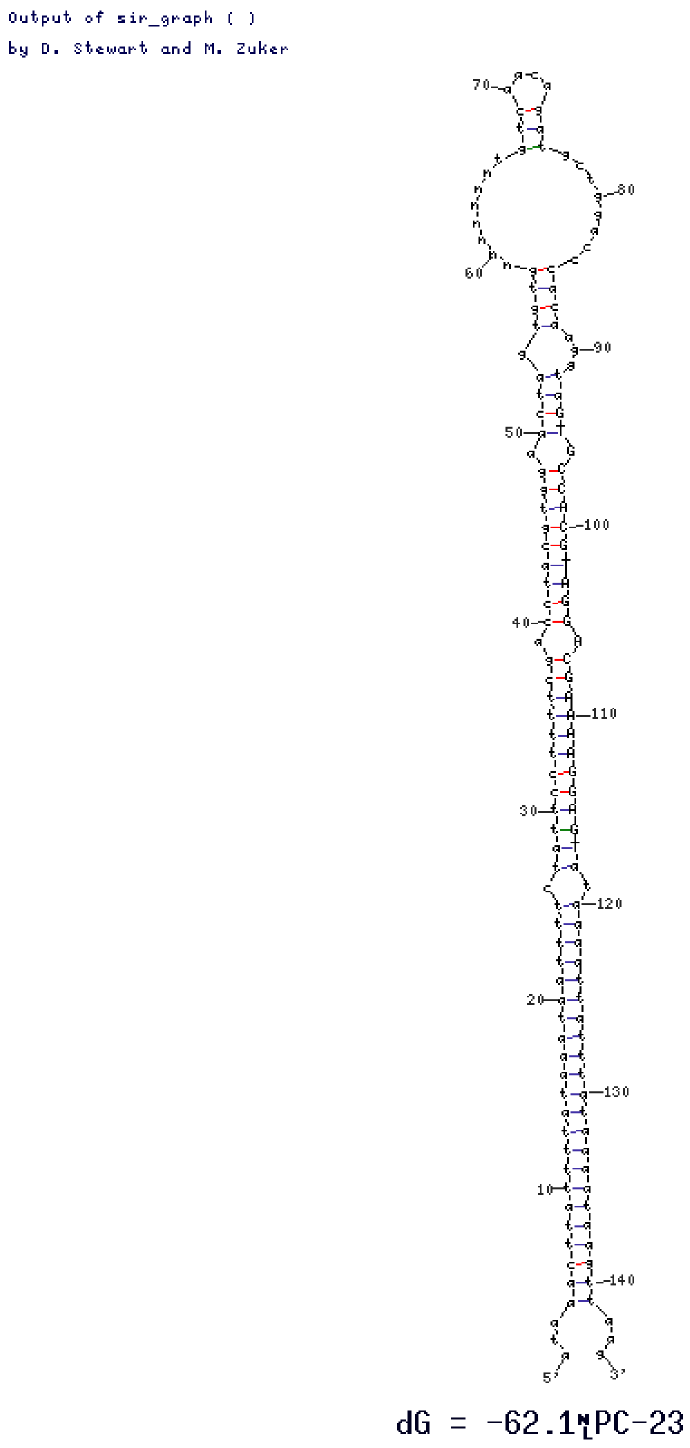
Output of sir_graph ( )
by D. Stewart and M. Zuker
70
80
60
90
50
100
40
110
30
120
20
130
10
140
5'
3'
dG = -62.1
PC-23

PC-23

Output of sir_graph ( )
by D. Stewart and M. Zuker

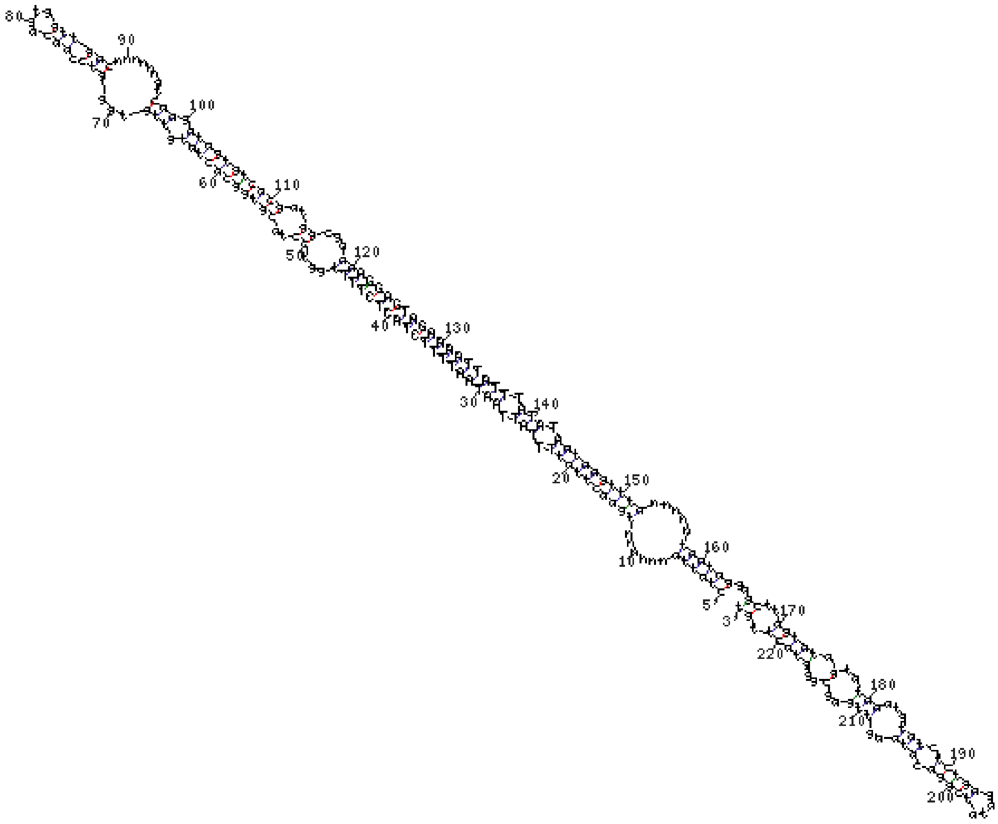

dG = -72.3 PC-24

PC-24

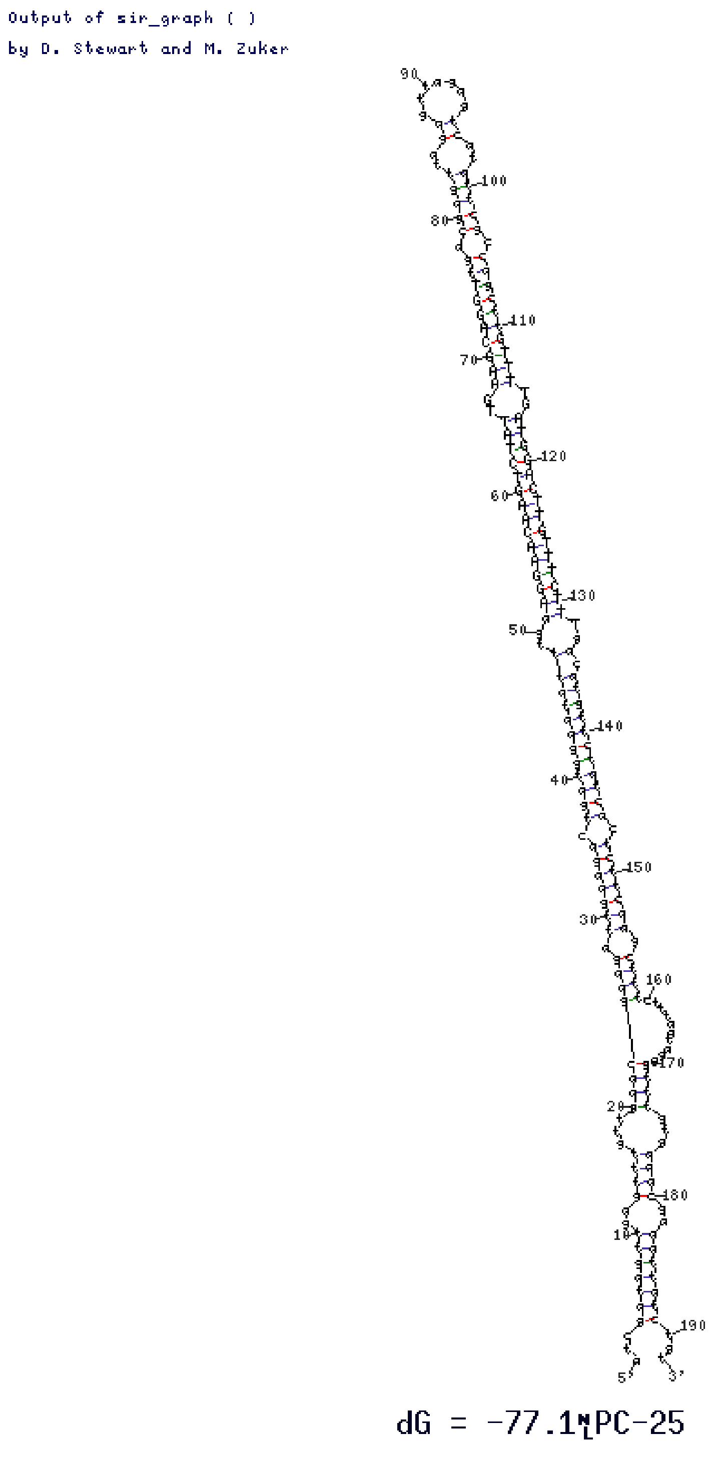

PC-25

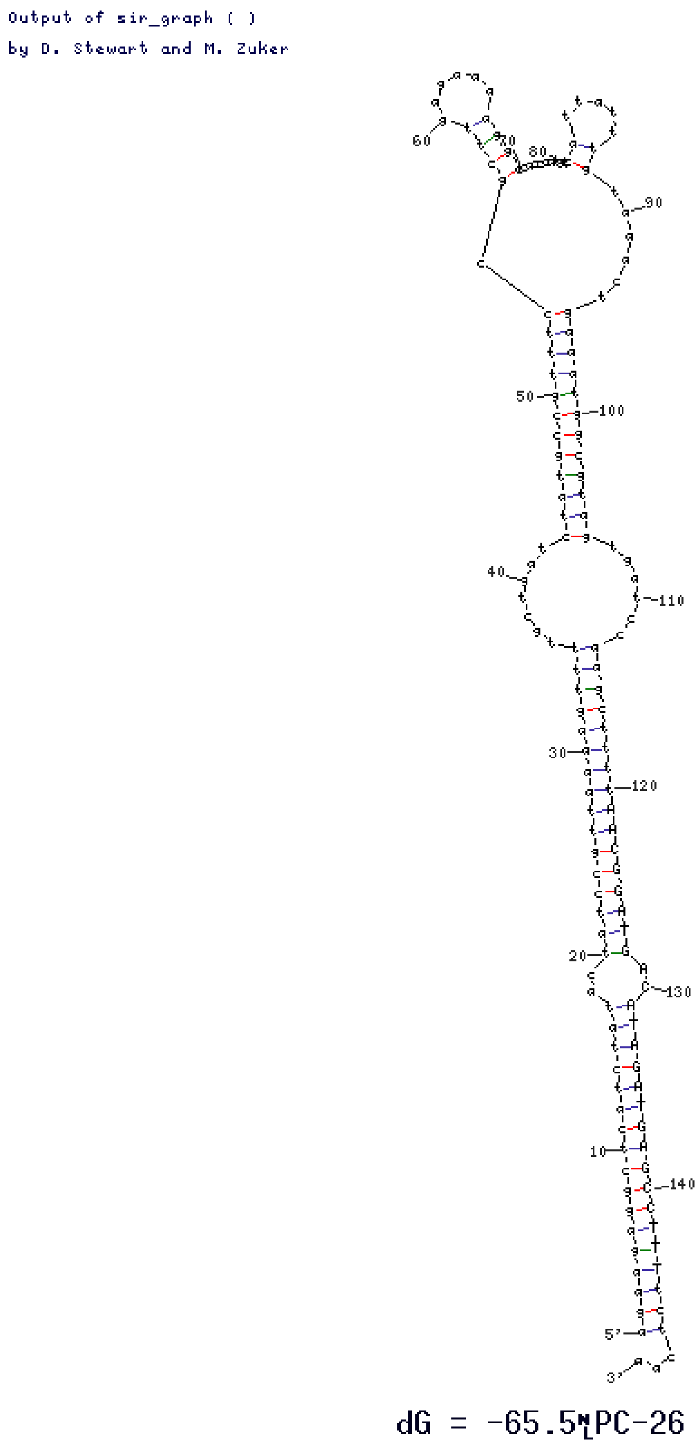

PC-26

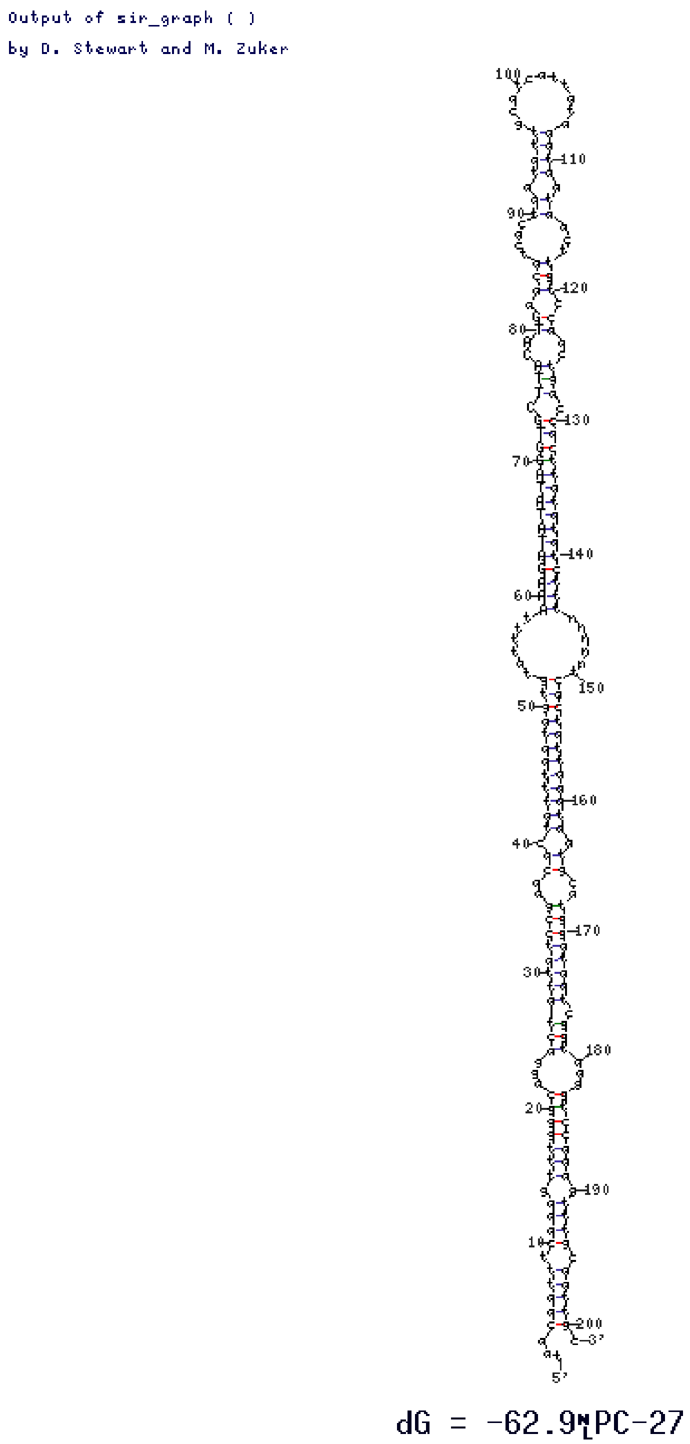

PC-27

Output of sir_graph ( )
by D. Stewart and M. Zuker

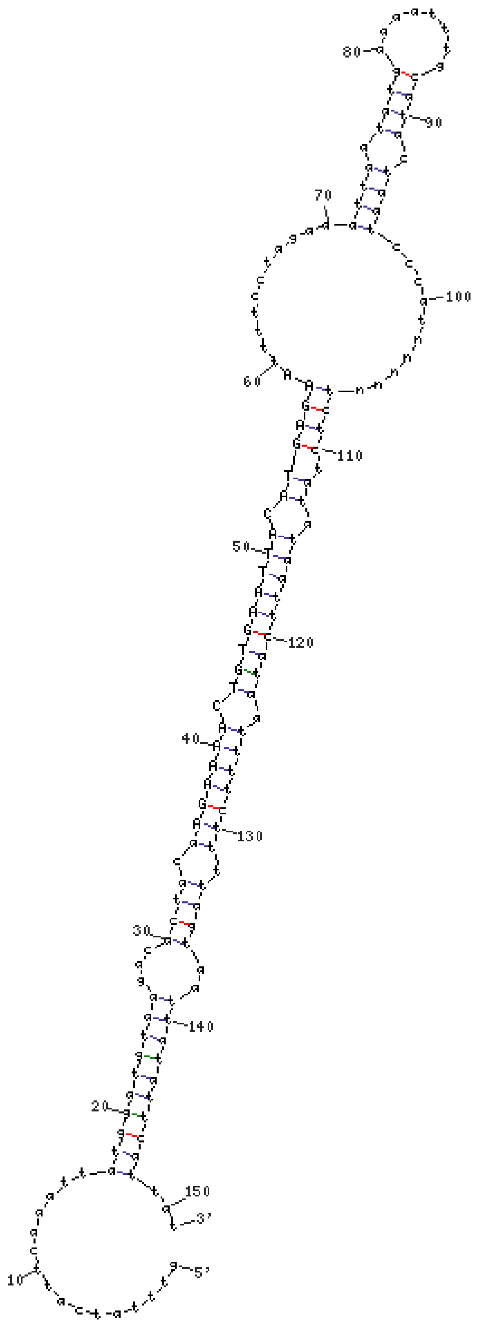

dG = -31.8 PC-28

PC-28

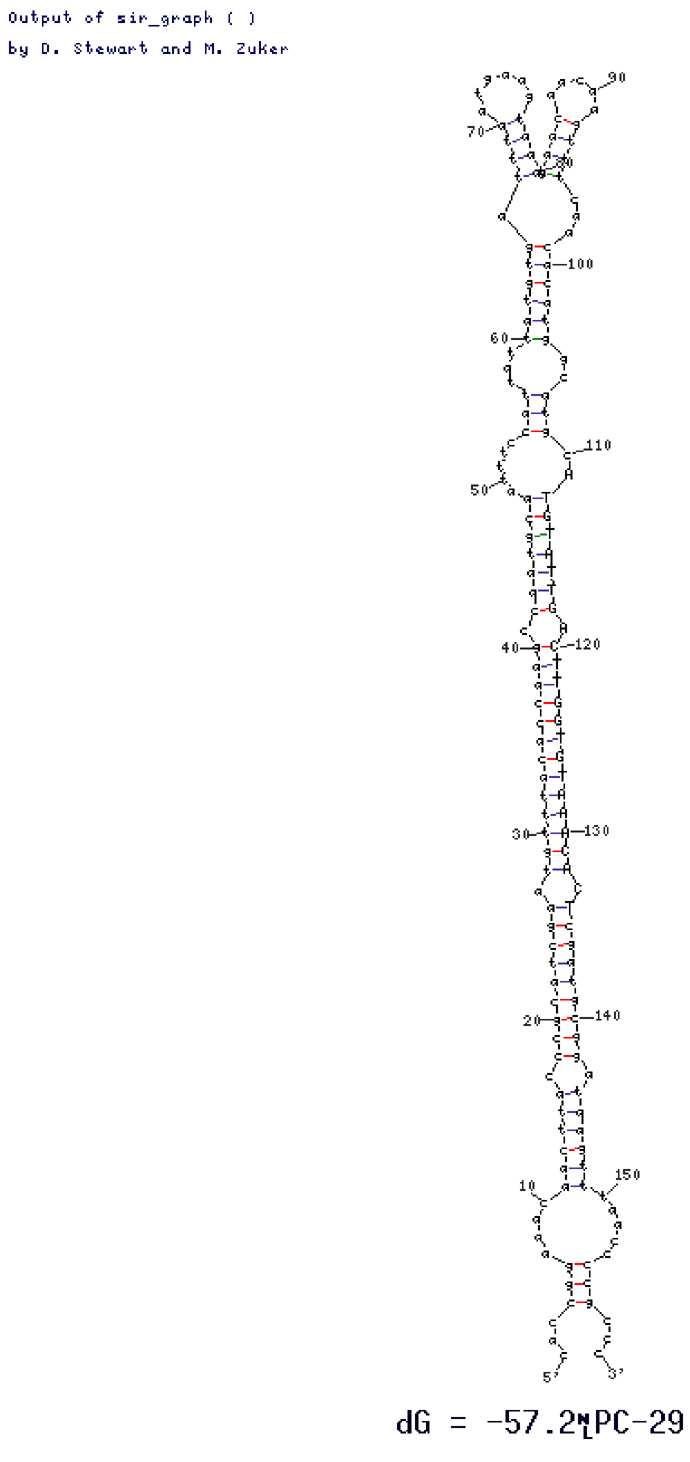

PC-29

Output of sir_graph ( )
by D. Stewart and M. Zuker

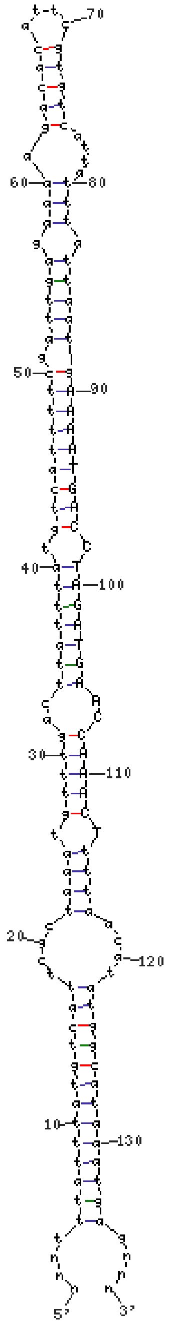

dG = -42.5 PC-30

PC-30

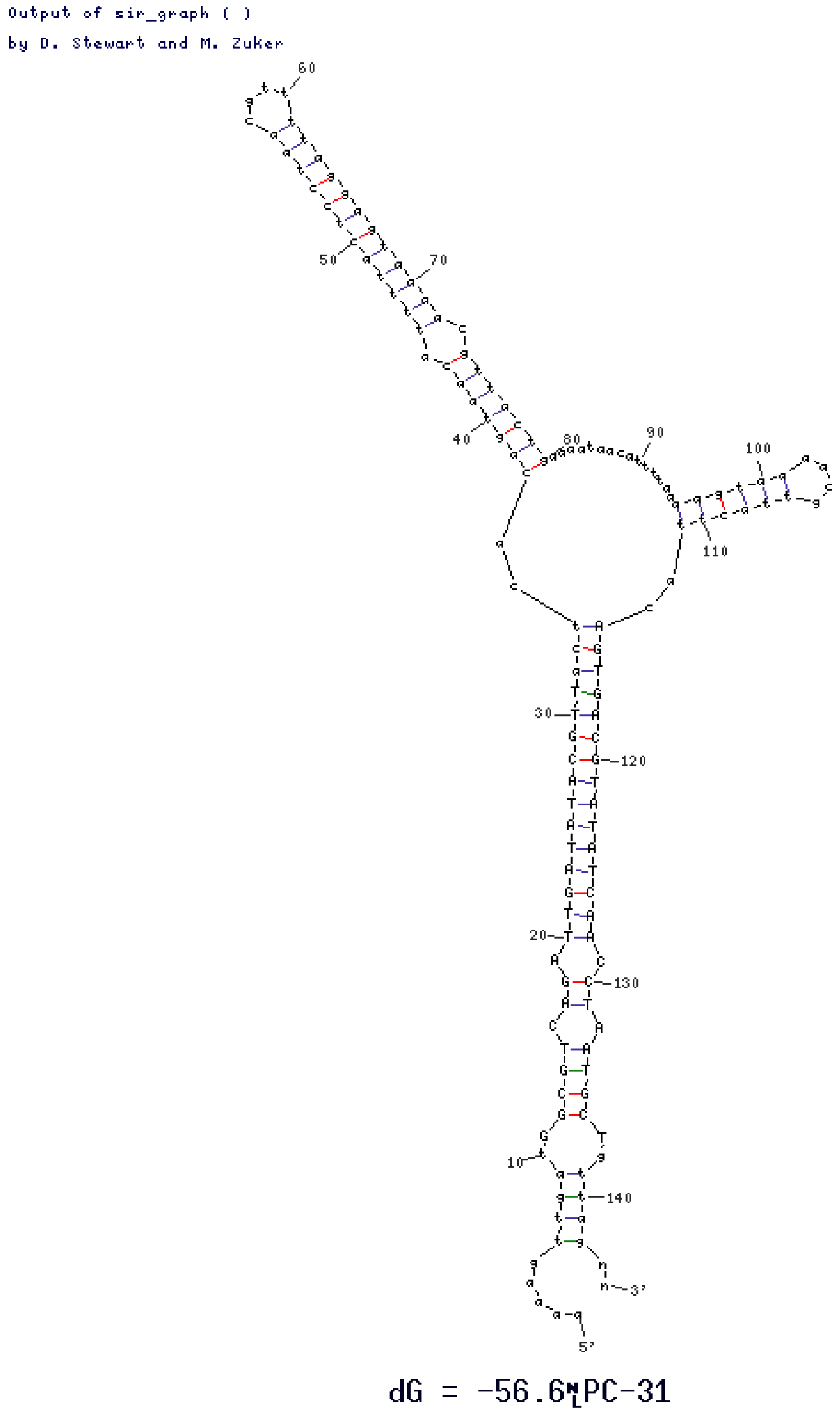
Output of sir_graph ( )
by D. Stewart and M. Zuker
60
50
70
40
80
90
100
110
30
120
20
130
10
140
3'
5'
dG = -56.6 PC-31

PC-31

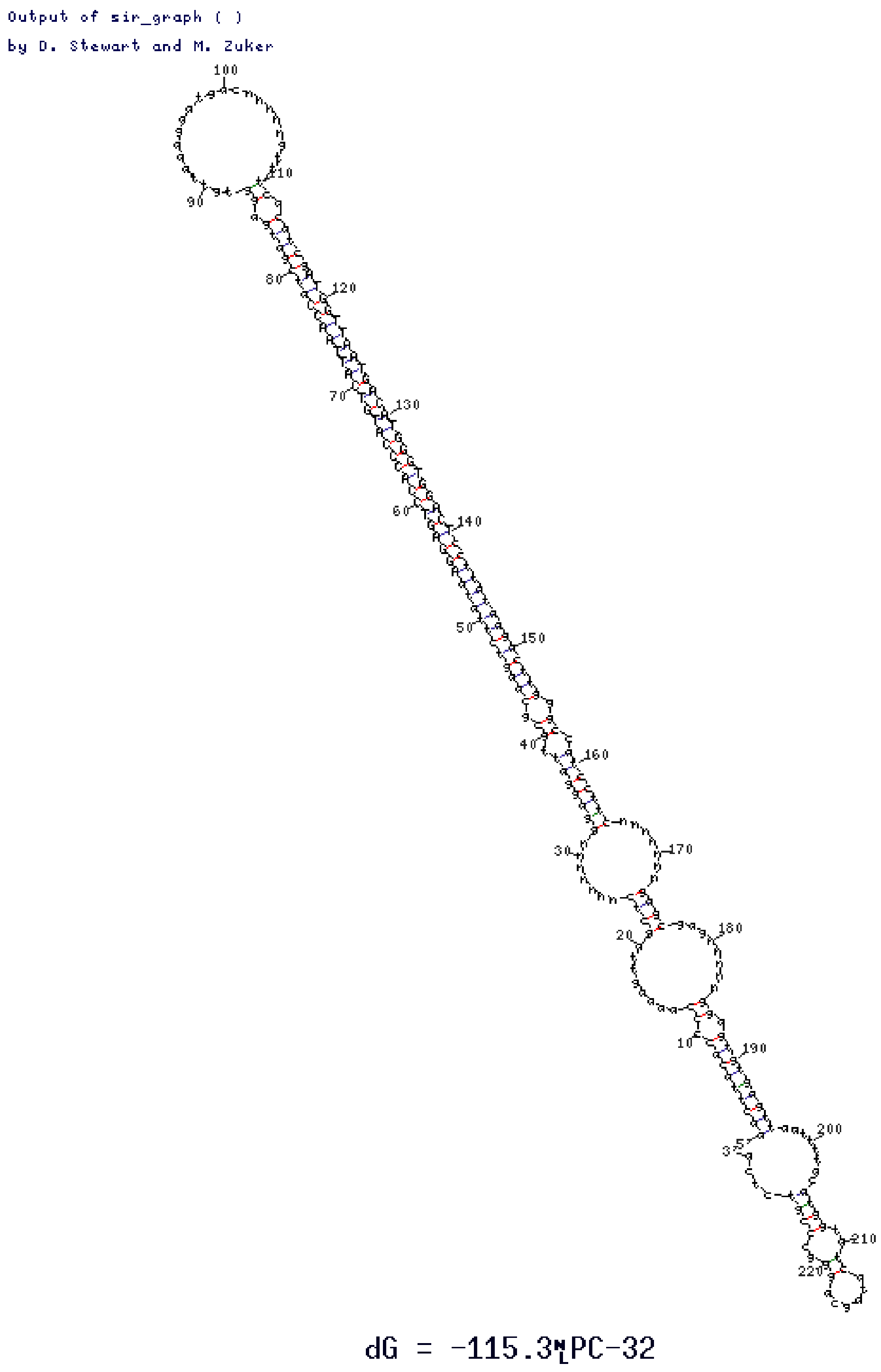

PC-32

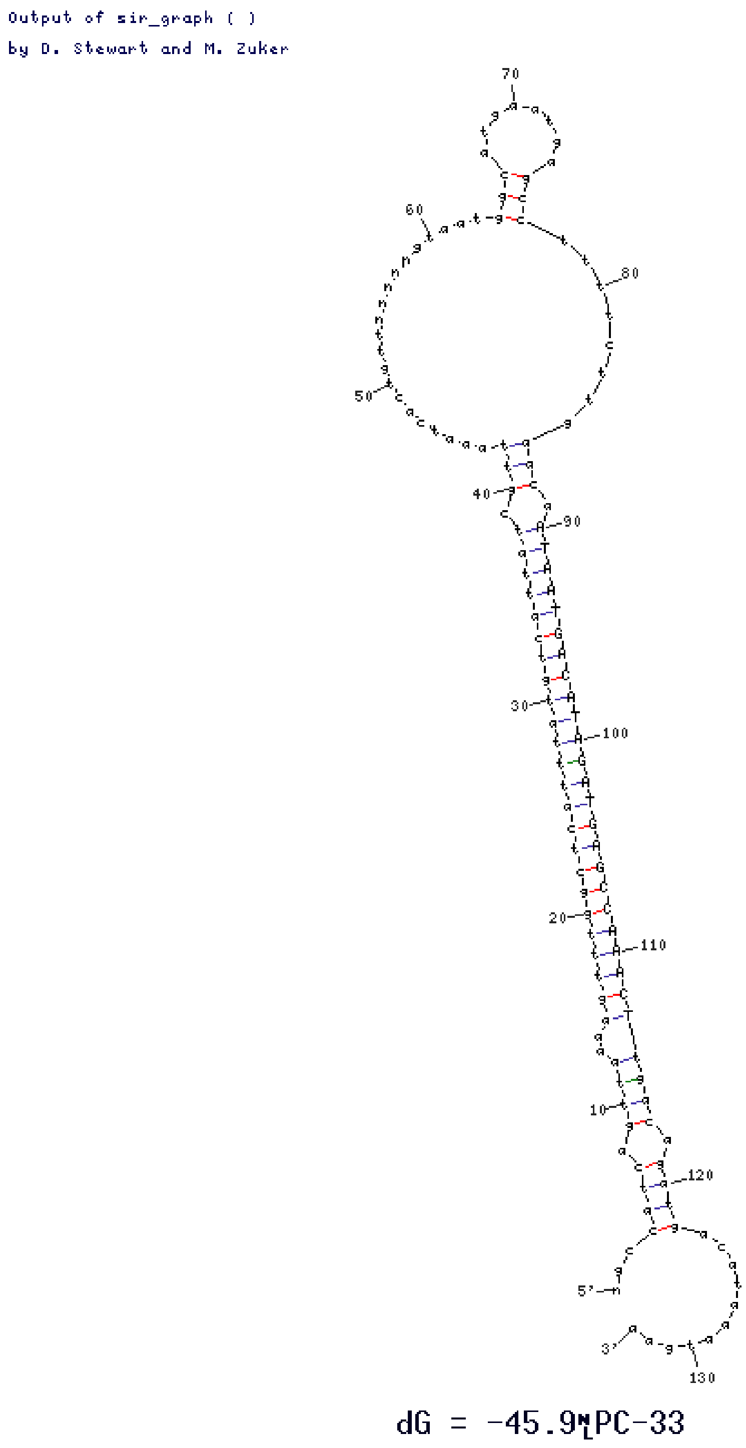

PC-33

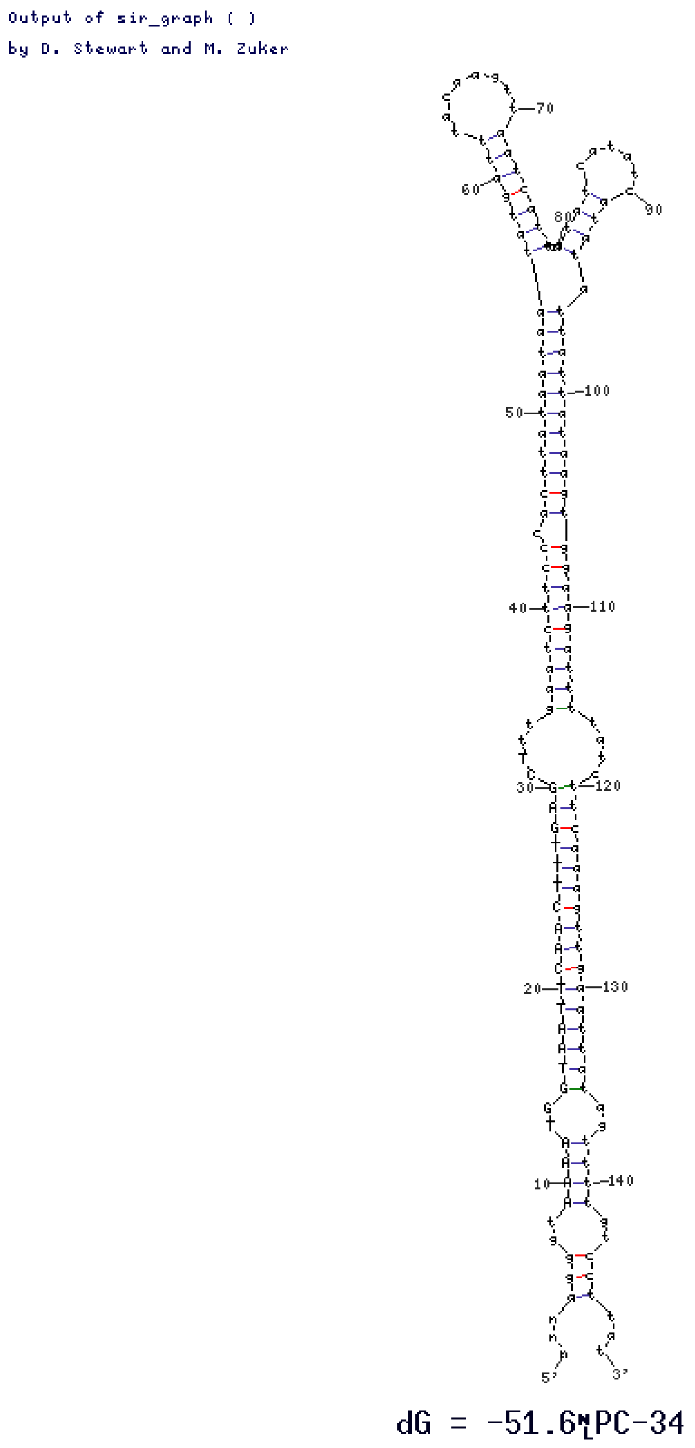

PC-34

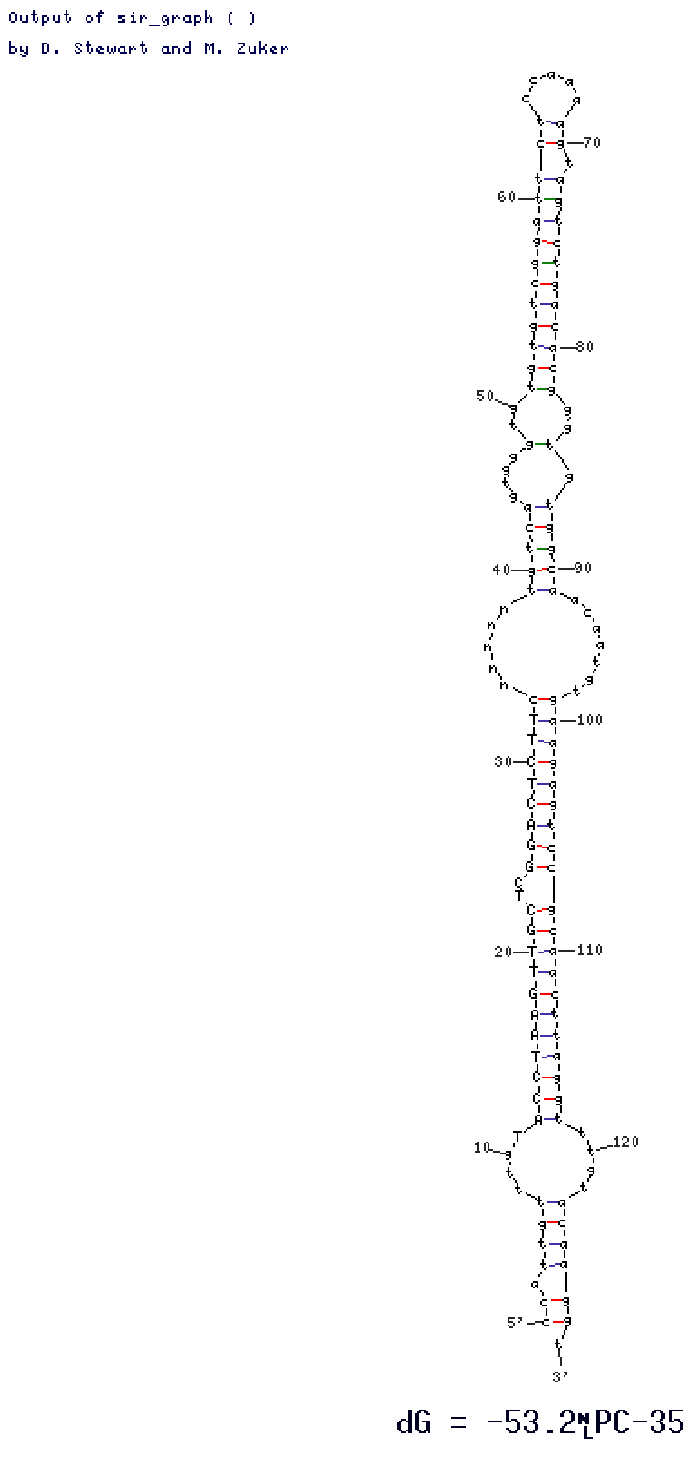

PC-35

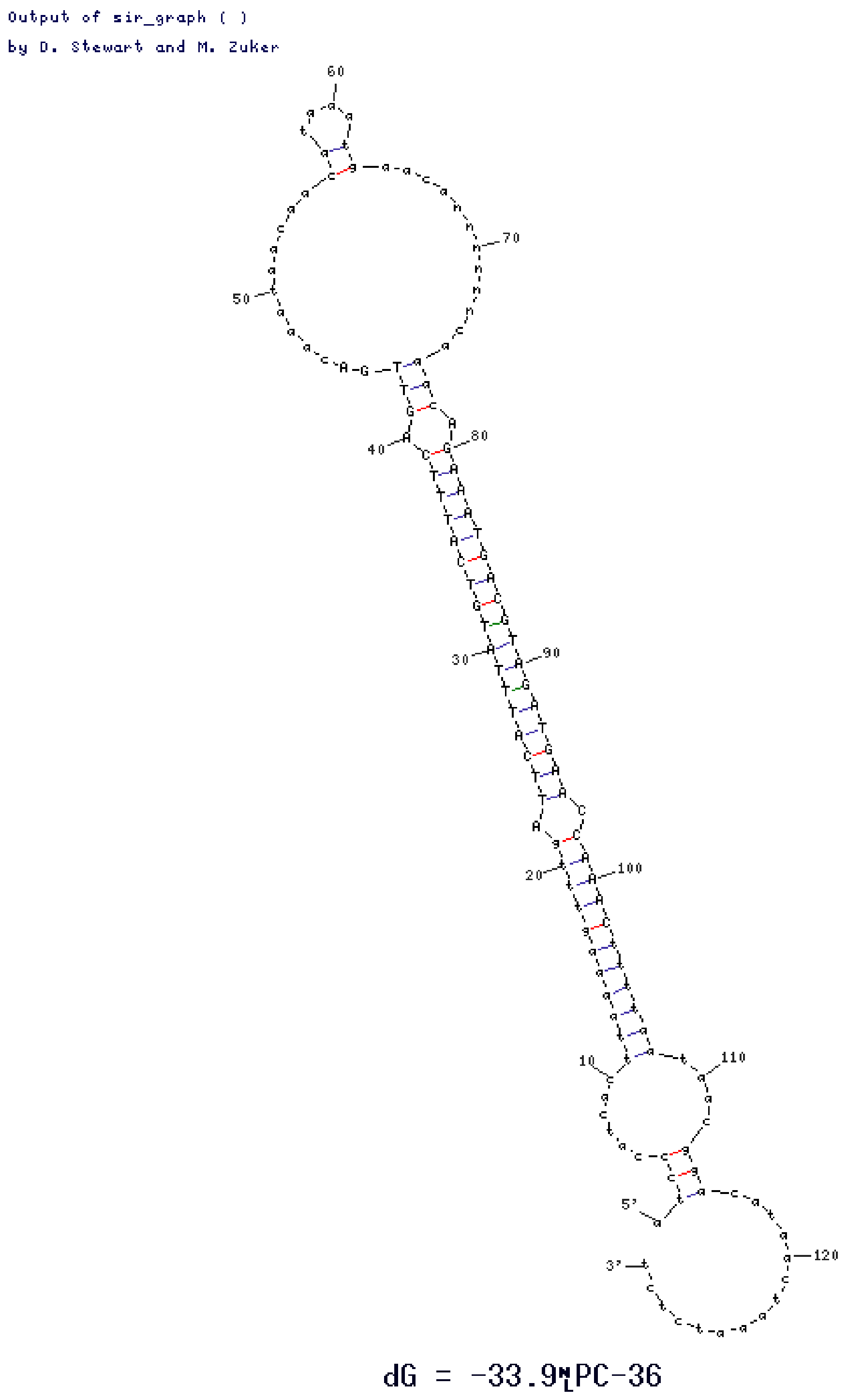

PC-36

Output of sir_graph ( )
by D. Stewart and M. Zuker

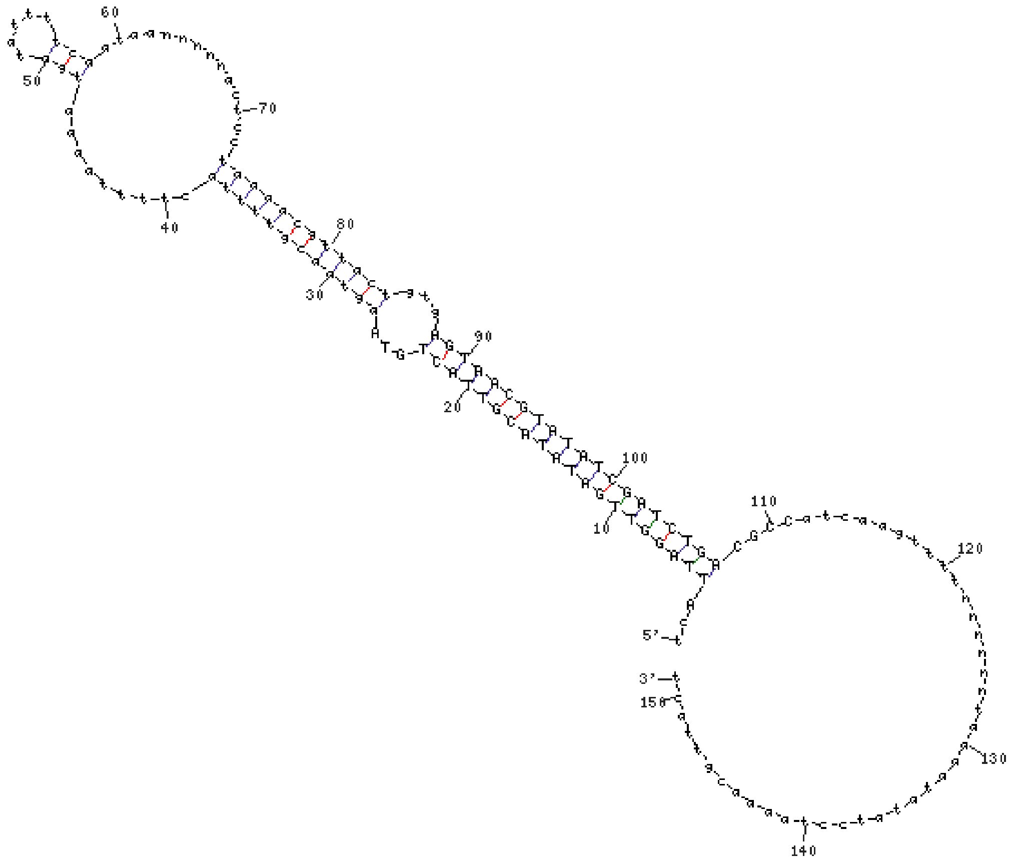

dG = -38.3 [illegible] PC-37

PC-37

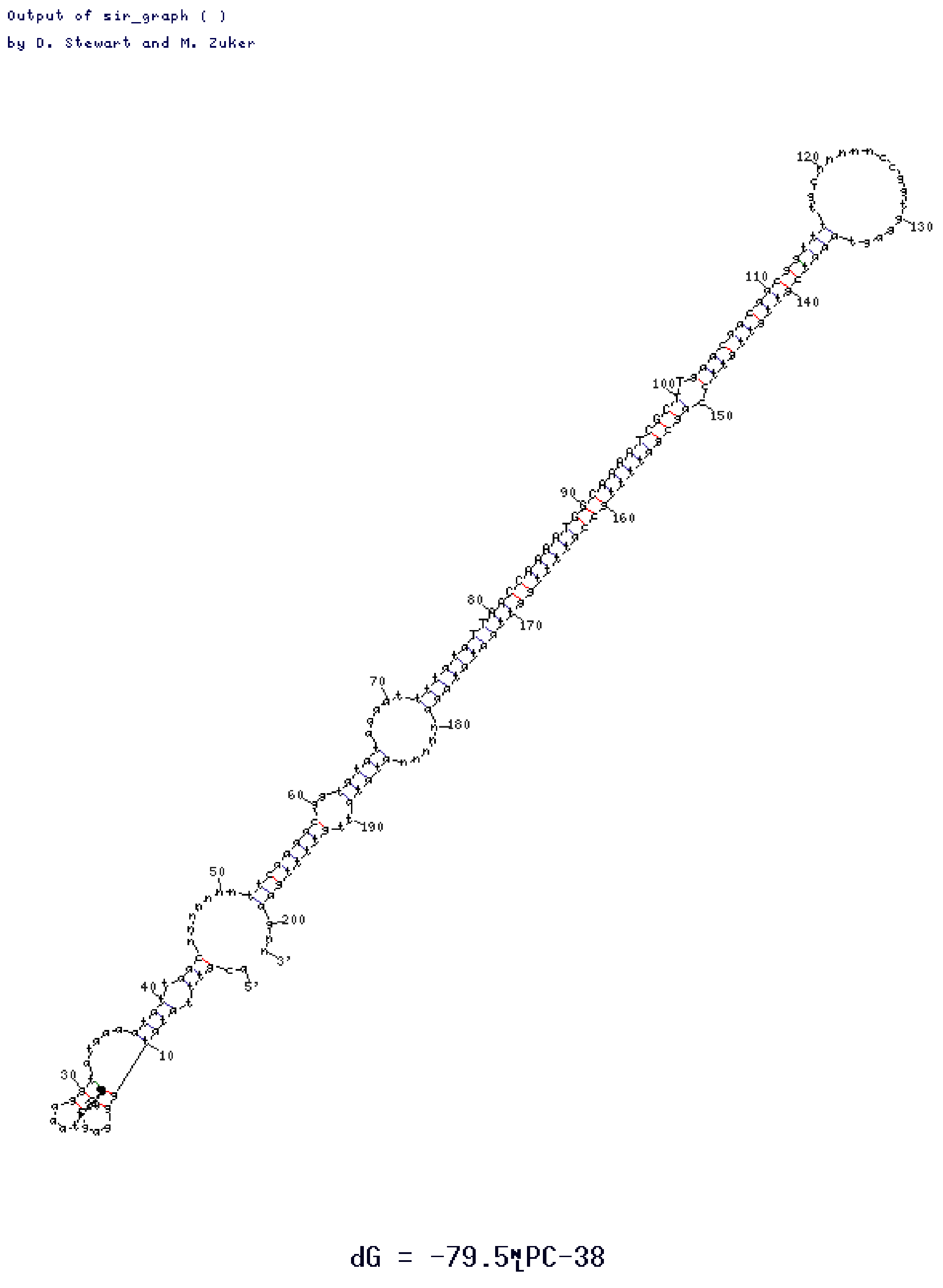
Output of sir_graph ( )
by D. Stewart and M. Zuker
120
130
110
140
100
150
90
160
80
170
70
180
60
190
50
200
3'
5'
40
10
30
dG = -79.5 PC-38

PC-38

Output of sir_graph ( )
by D. Stewart and M. Zuker

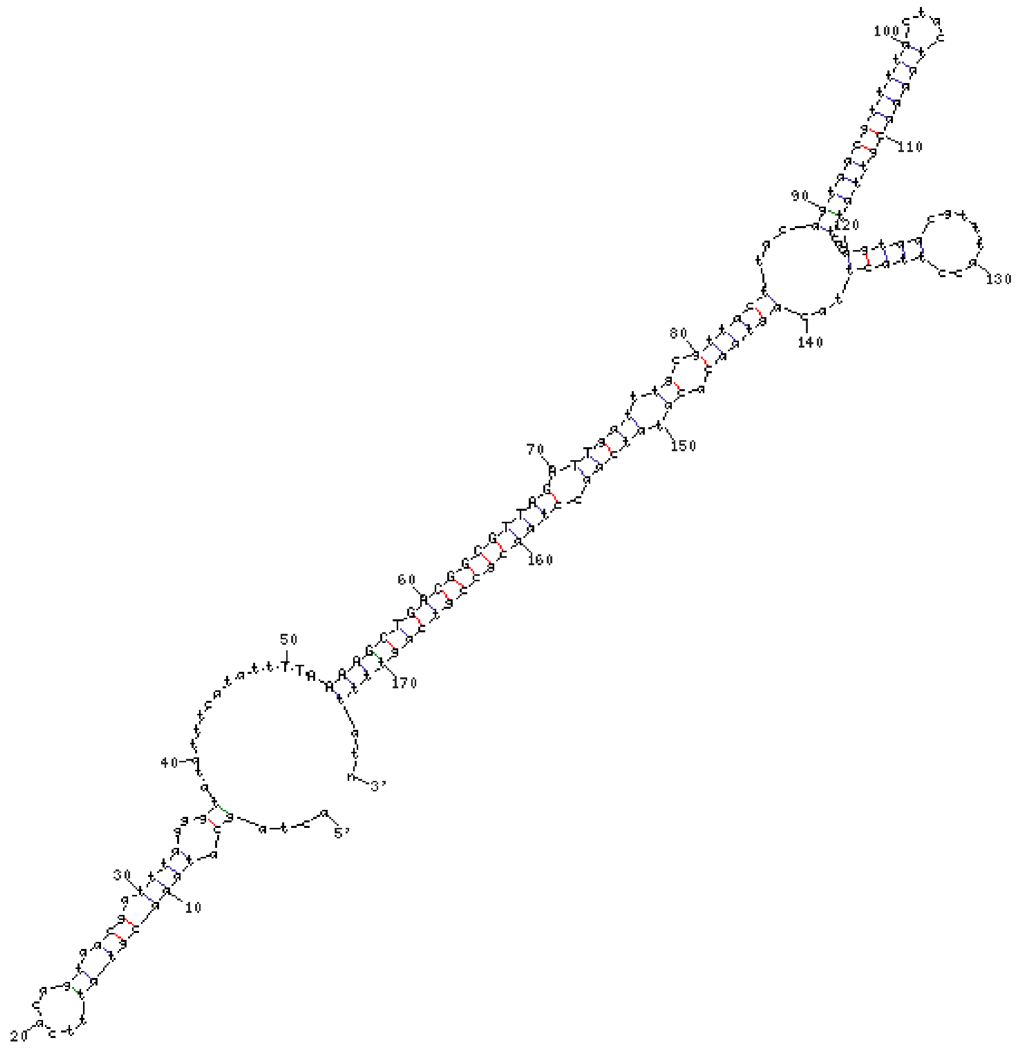

dG = -55.7 PC-39

PC-39

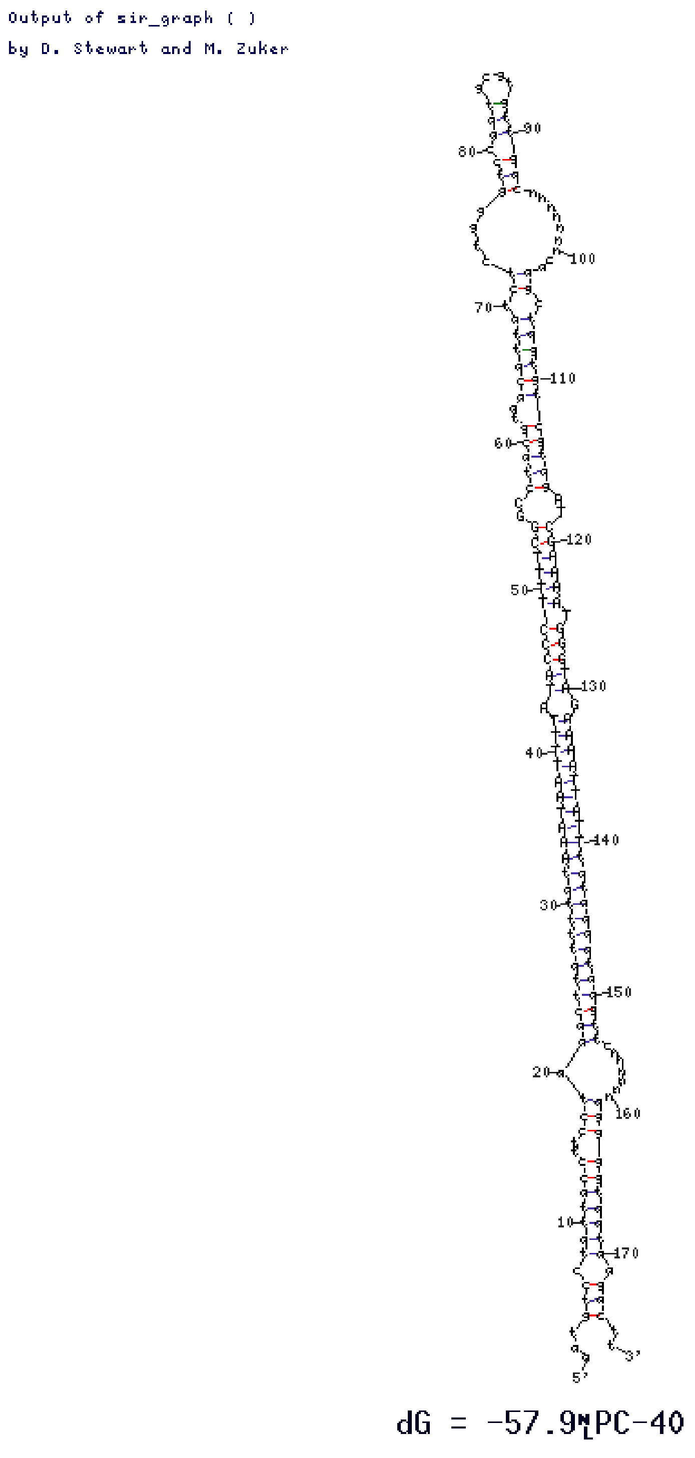

PC-40

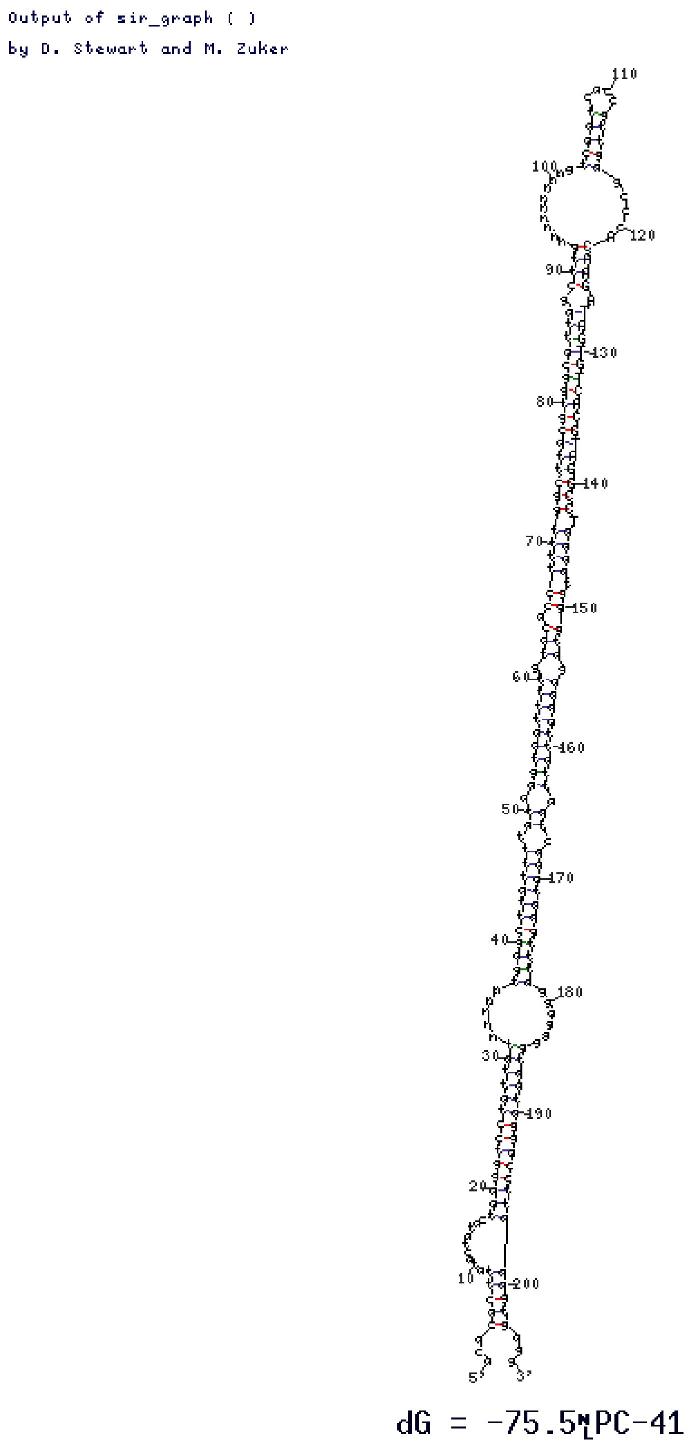
Output of sir_graph ( )
by D. Stewart and M. Zuker
110
100
120
90
130
80
140
70
150
60
160
50
170
40
180
30
190
20
10
200
5'
3'
dG = -75.5
PC-41

PC-41

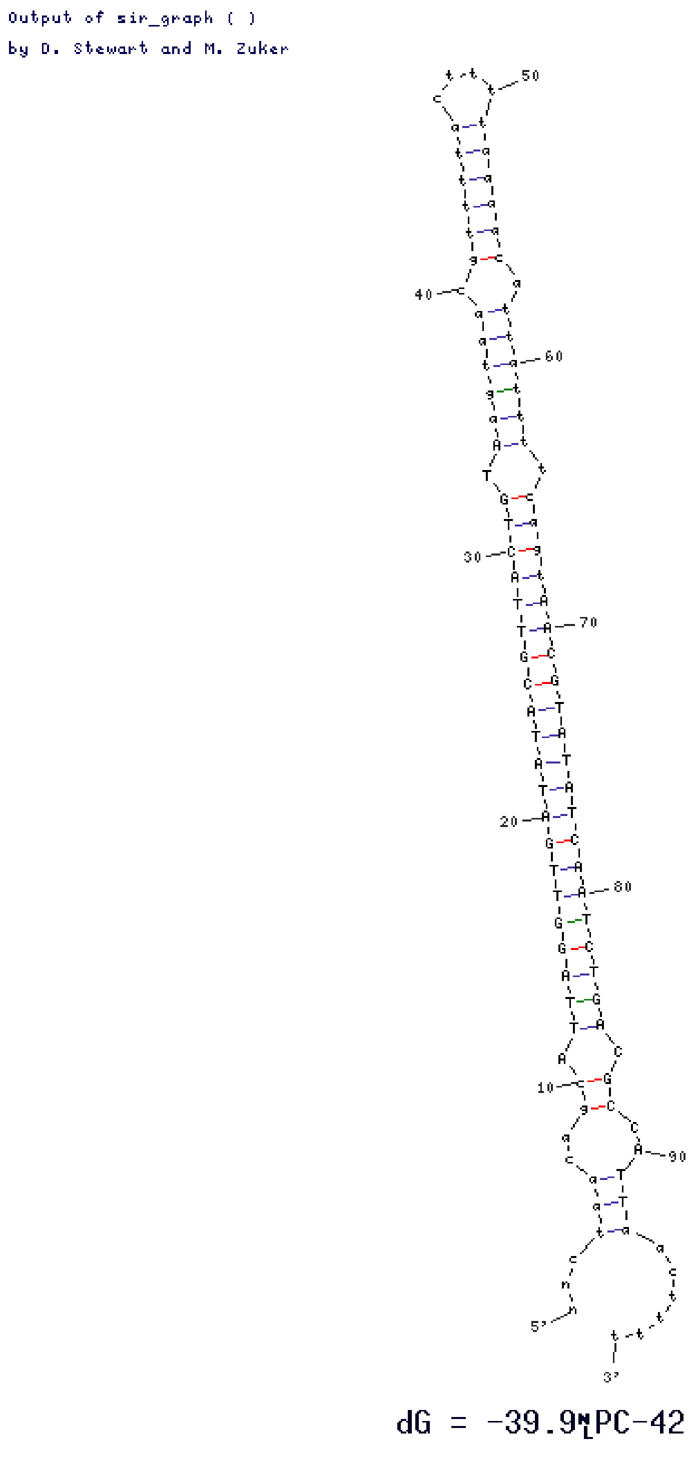

PC-42

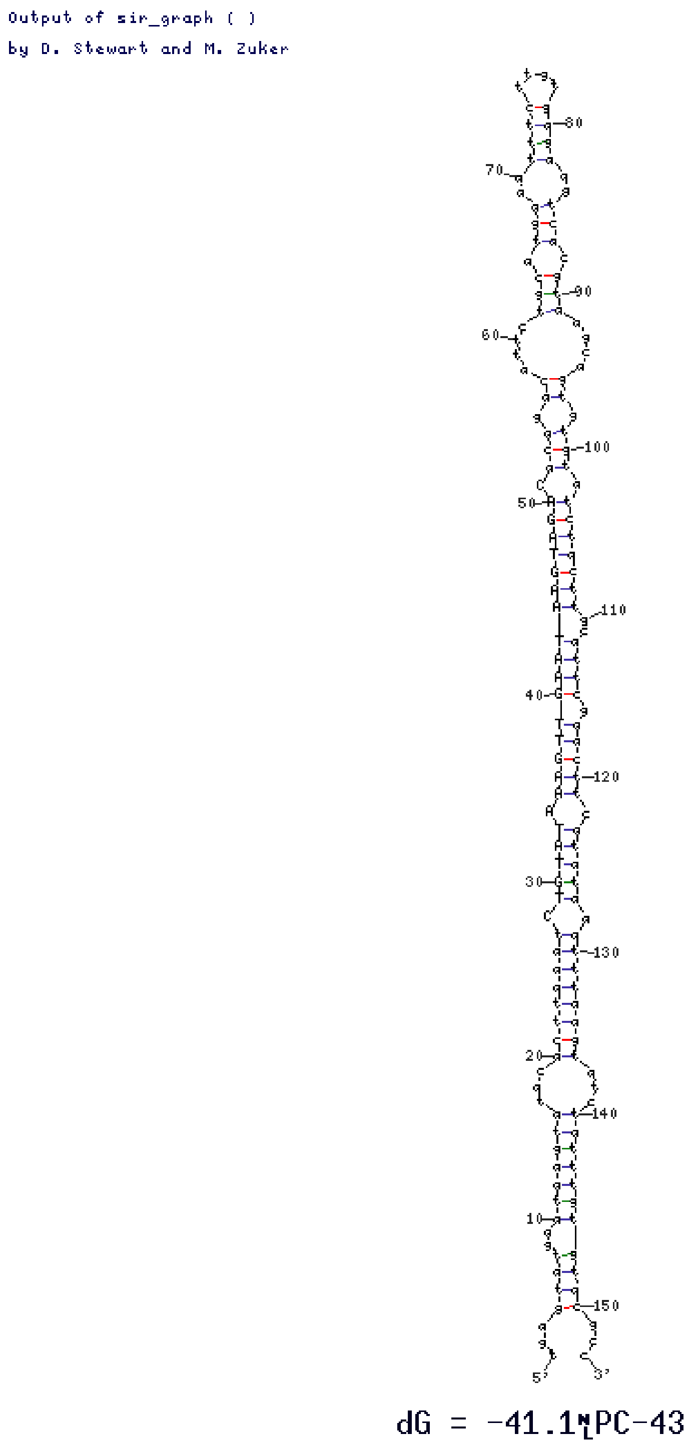

Output of sir_graph ( )
by D. Stewart and M. Zuker
80
70
90
60
100
50
110
40
120
30
130
20
140
10
150
5'
3'
dG = -41.1 PC-43

PC-43

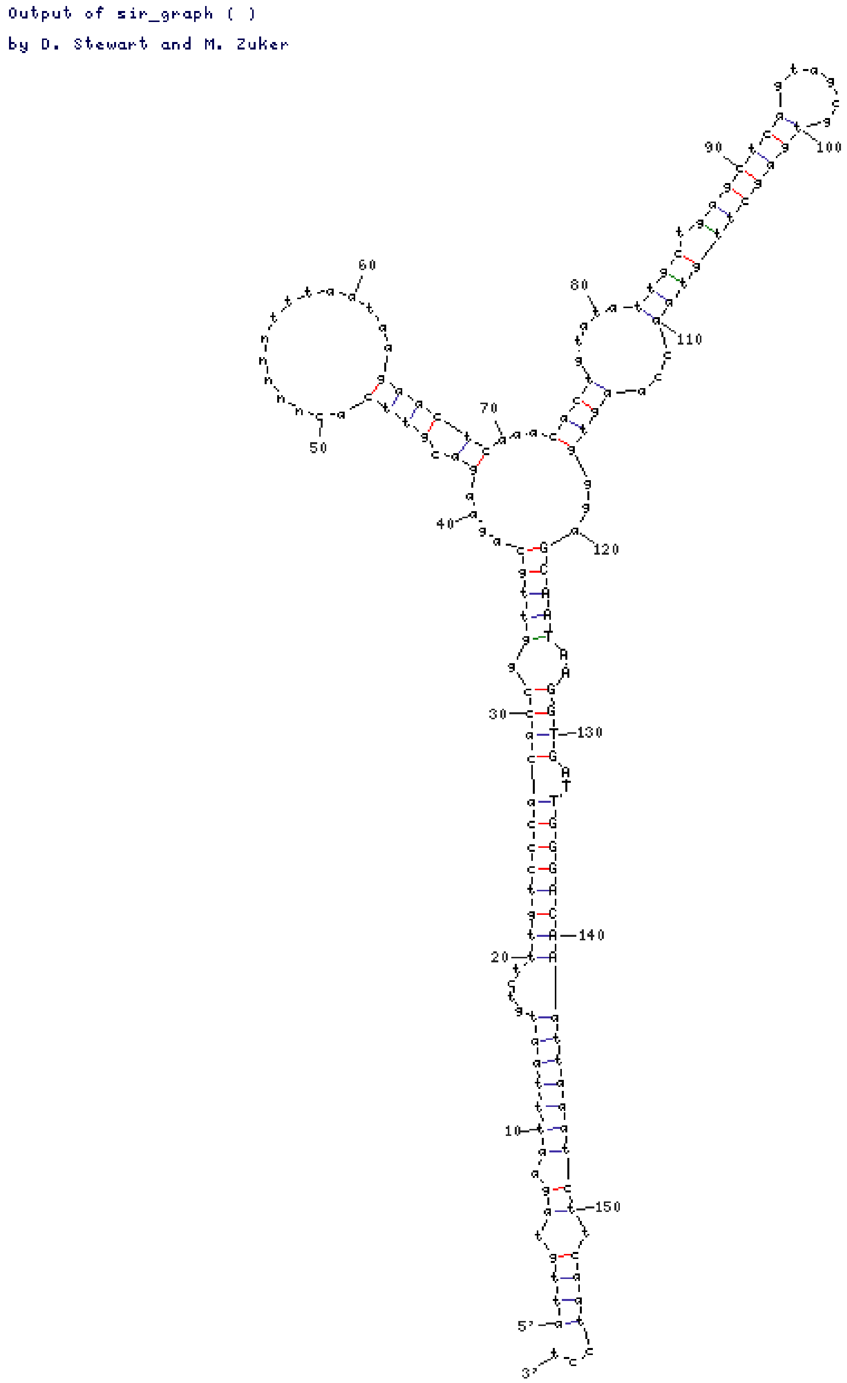

dG = −45[illegible]PC-44

PC-44

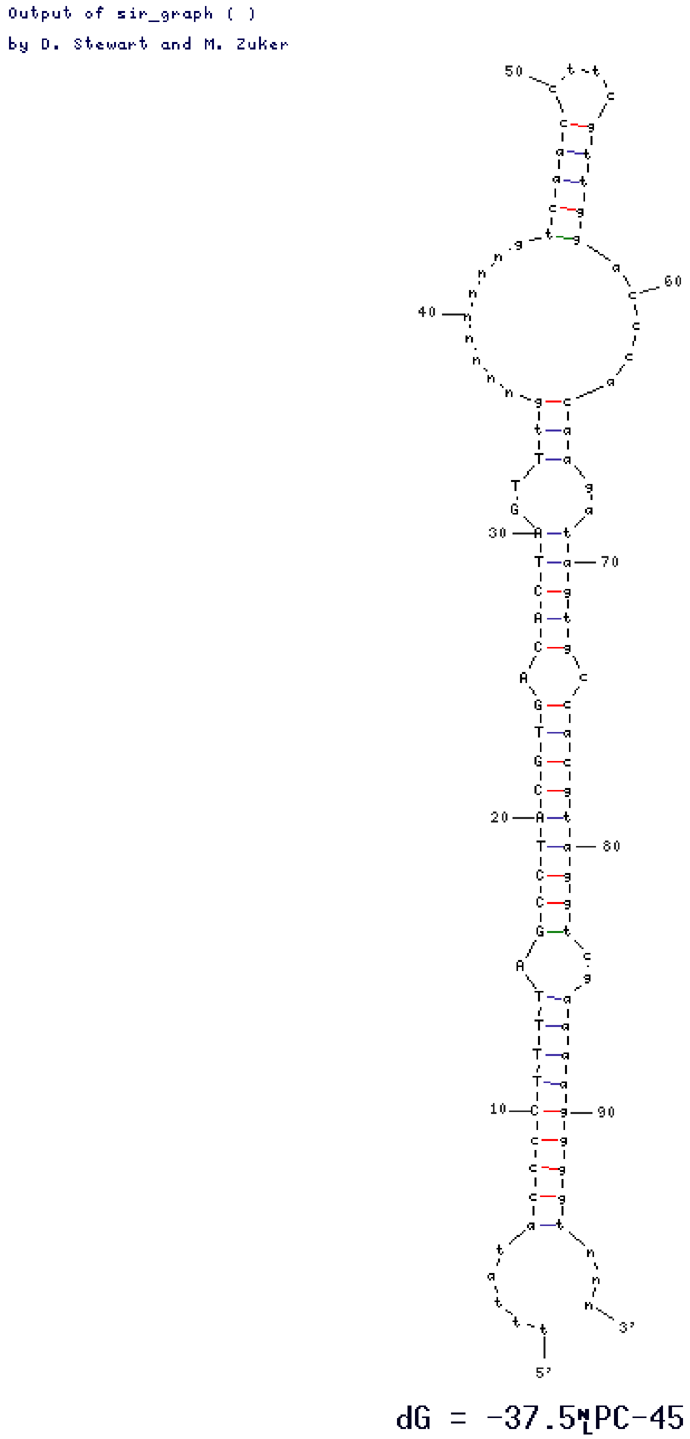

PC-45

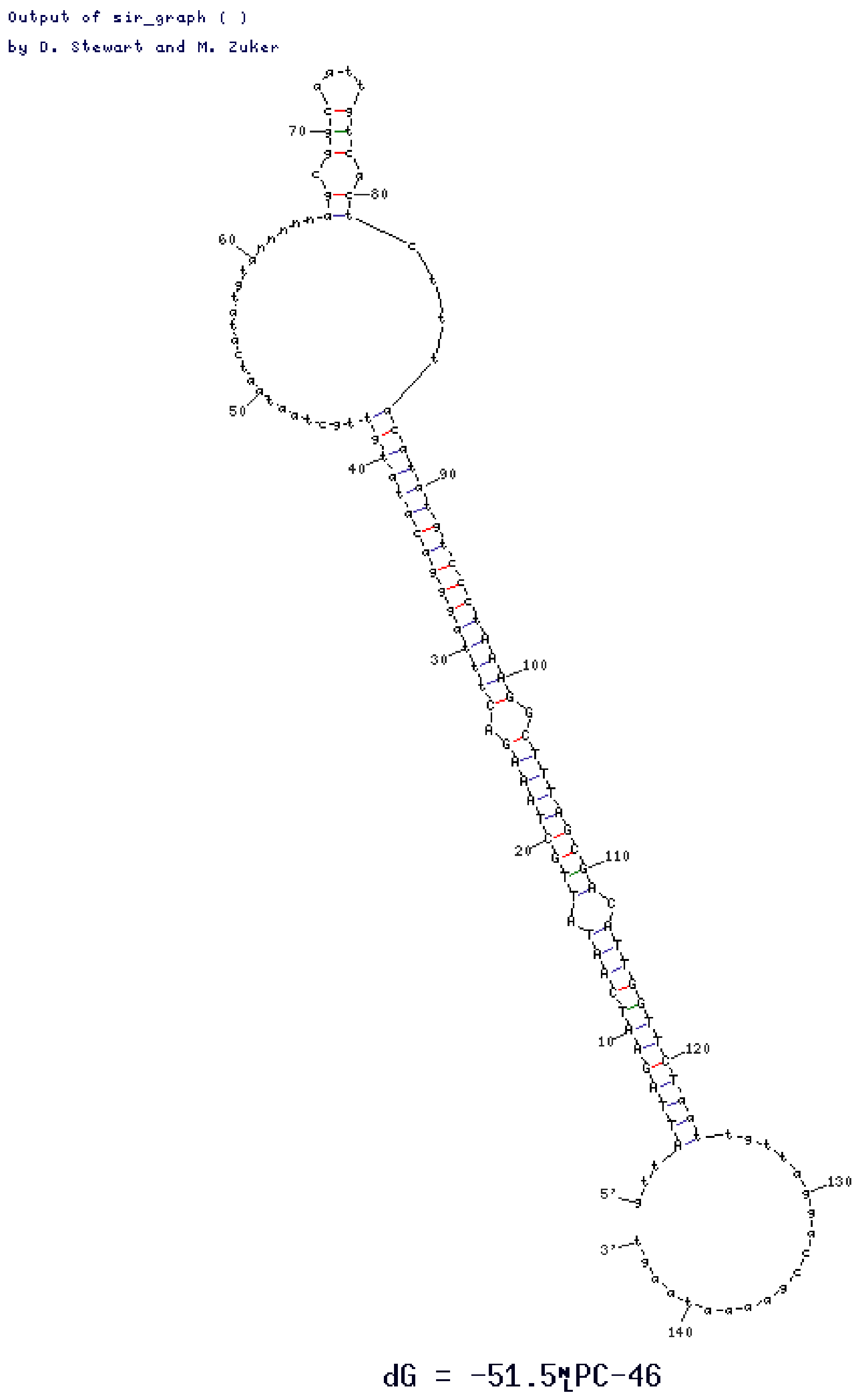

PC-46

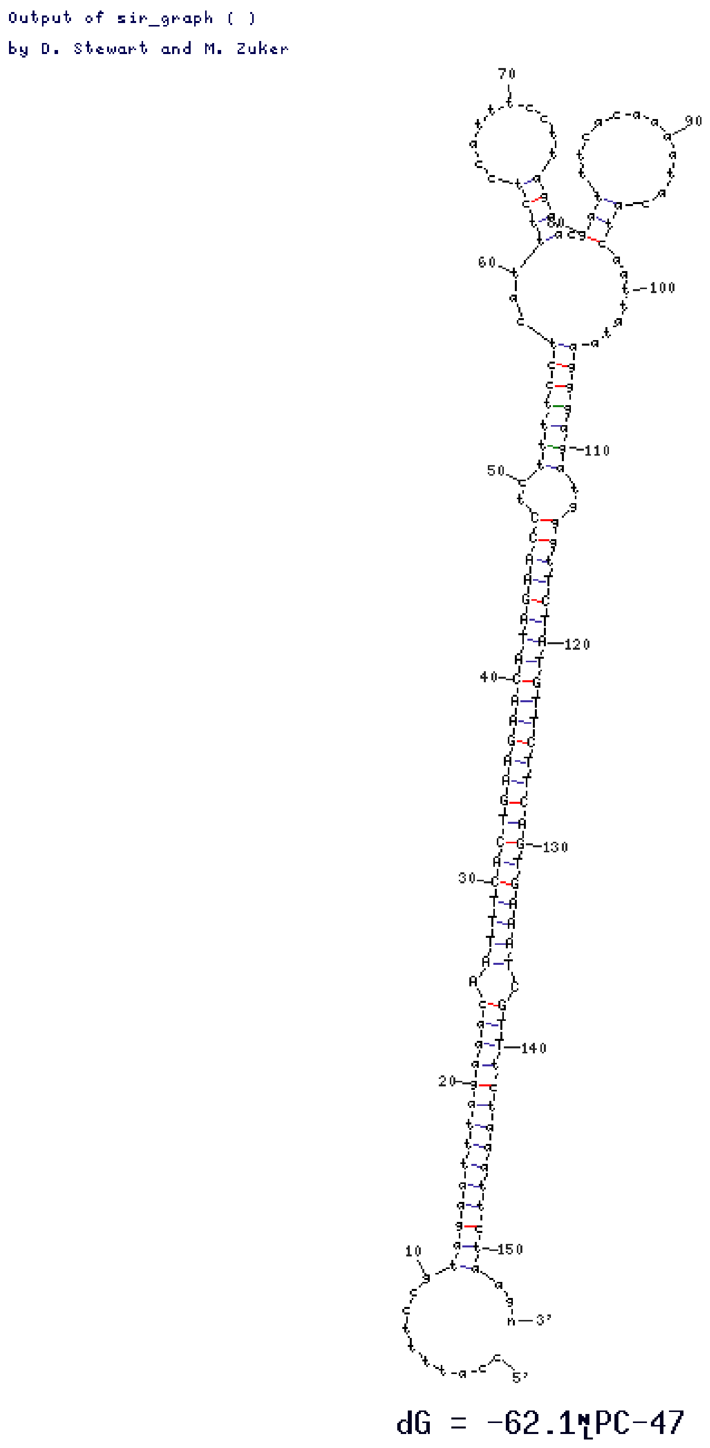

PC-47

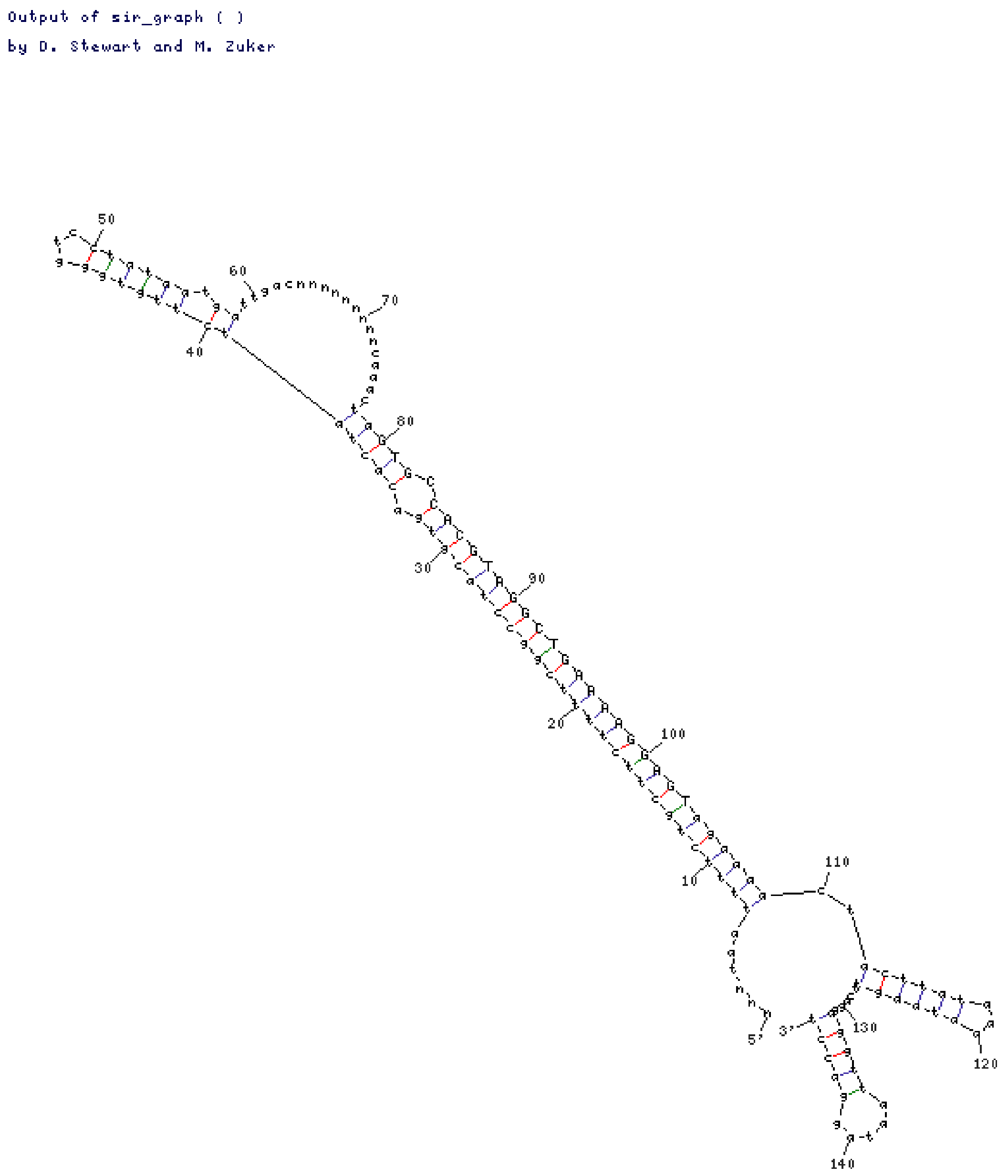

dG = -52.7 [illegible]PC-48

PC-48

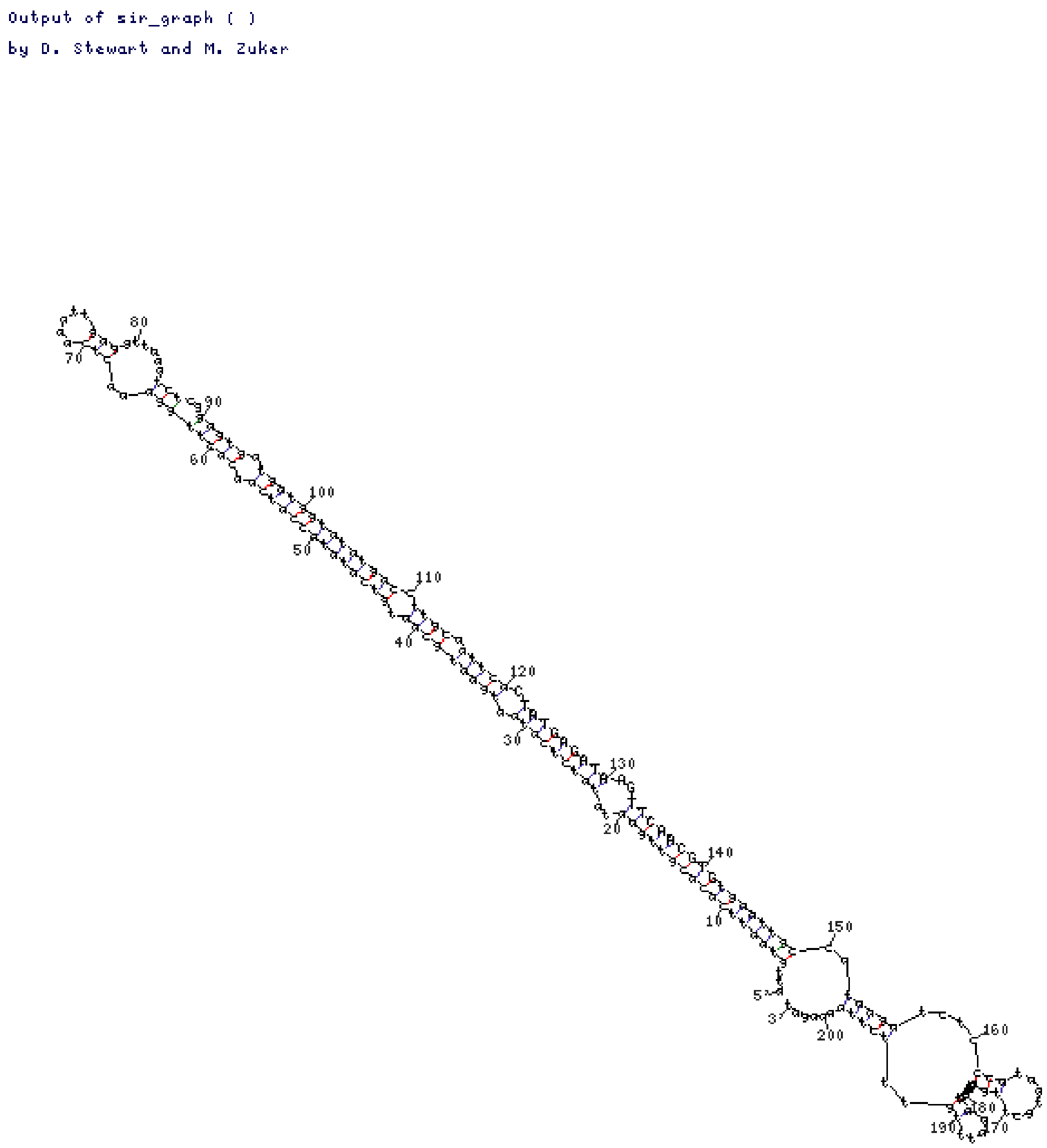

dG = -81.1 PC-49

PC-49

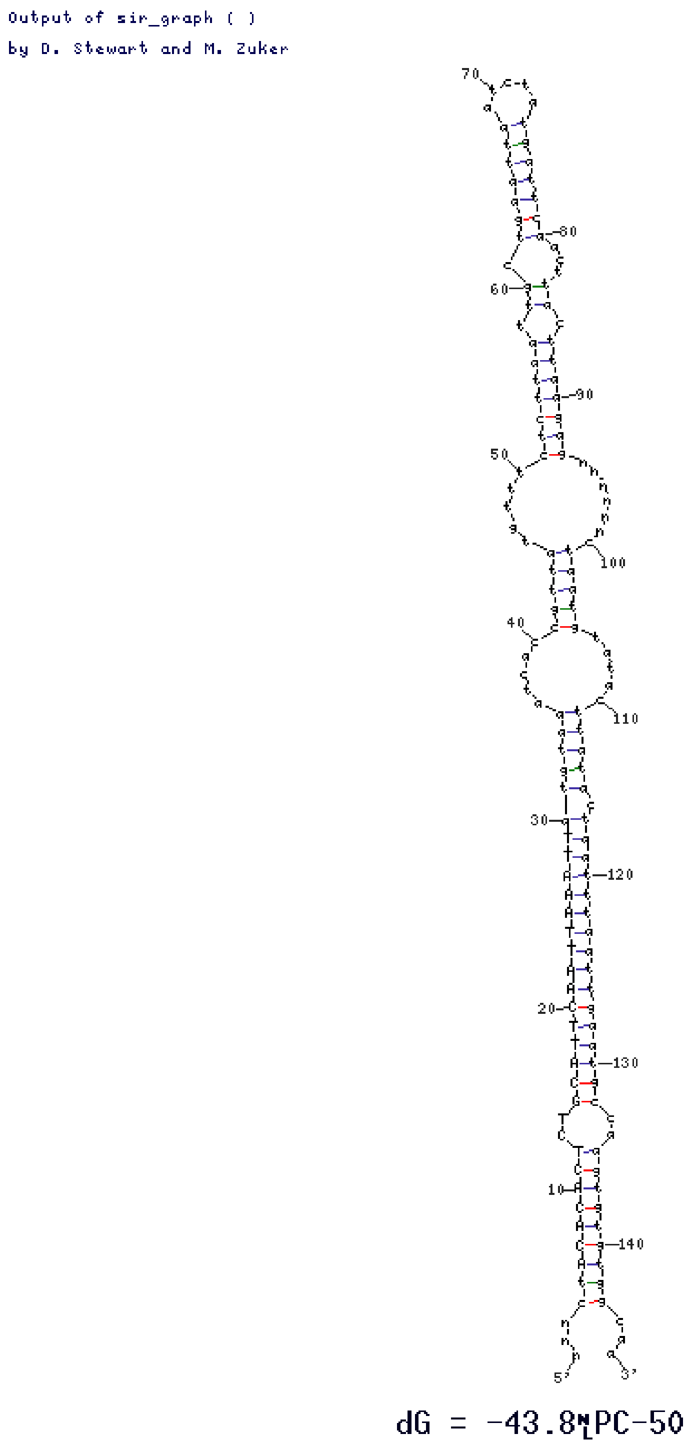
Output of sir_graph ( )
by D. Stewart and M. Zuker
70
80
60
90
50
100
40
110
30
120
20
130
10
140
5'
3'
dG = -43.8
PC-50

PC-50

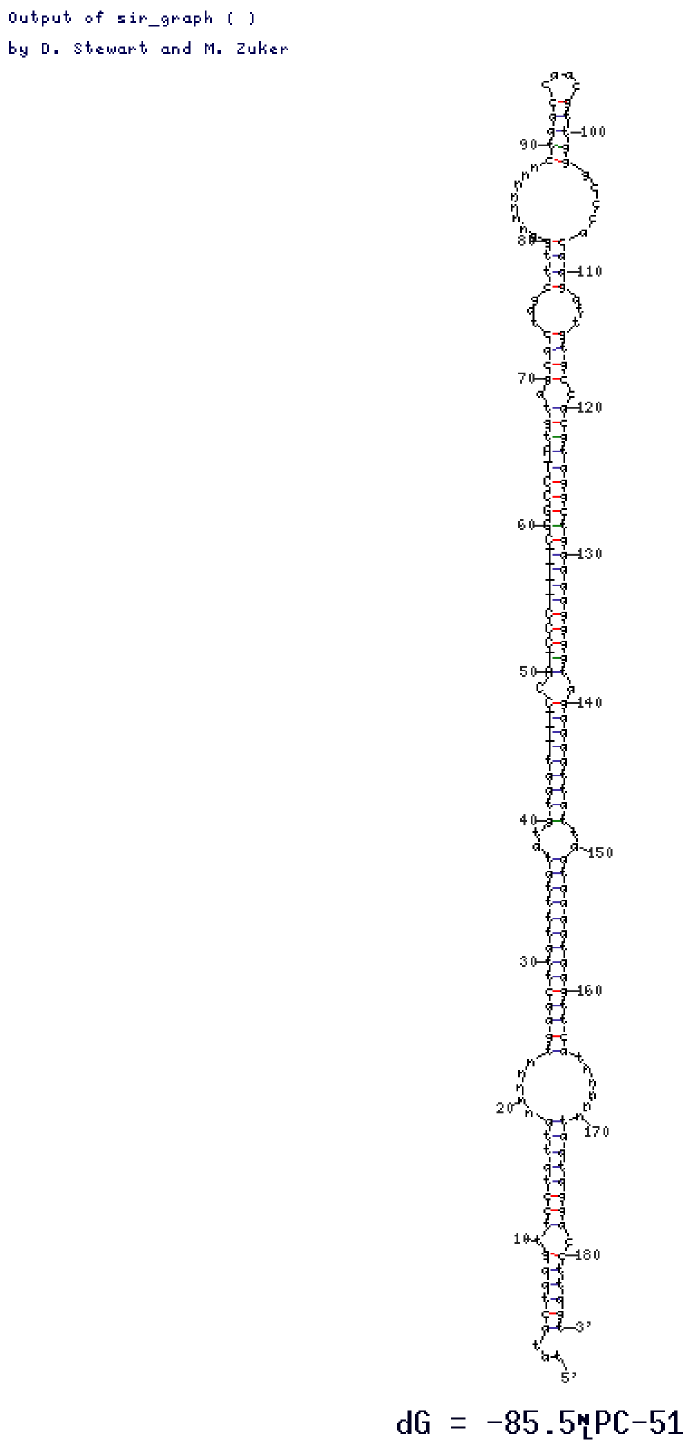

PC-51

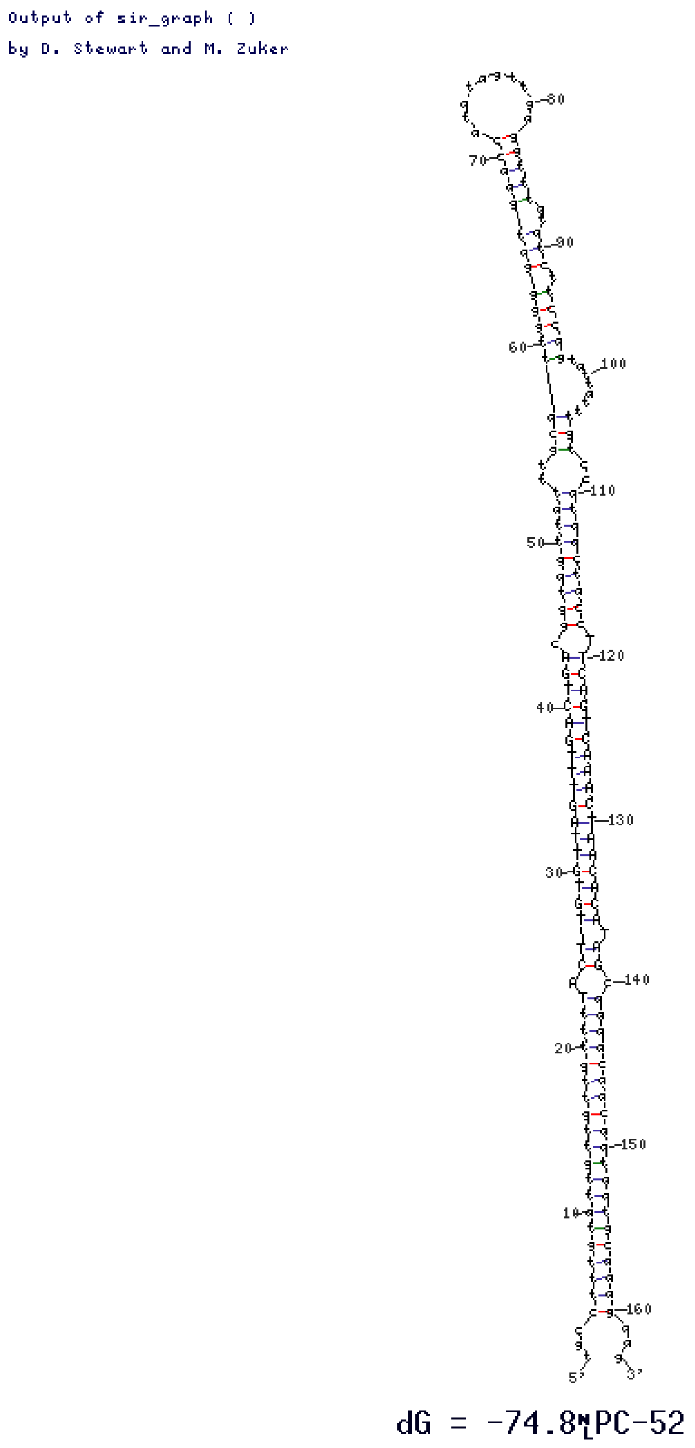

PC-52

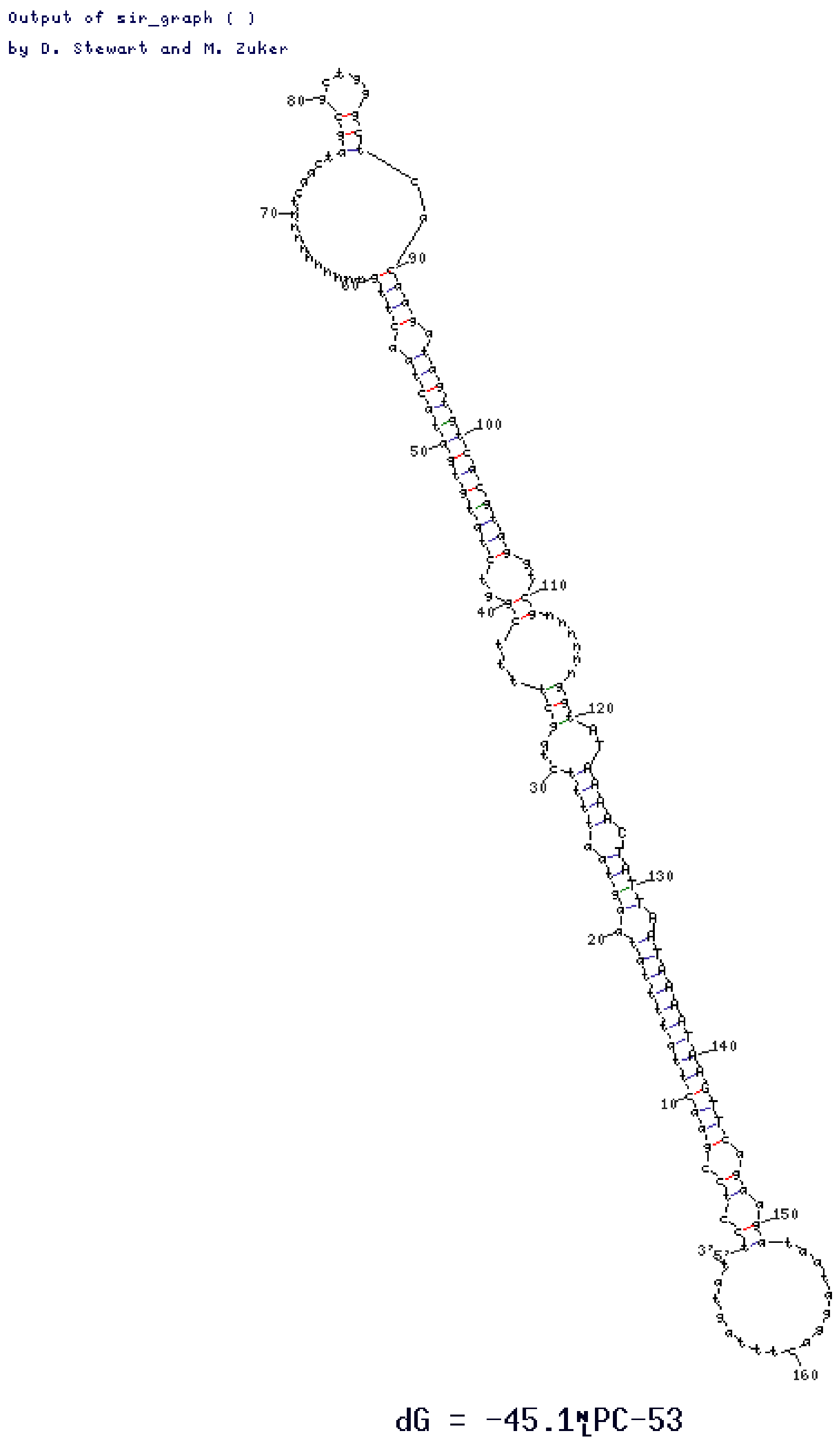
Output of sir_graph ( )
by D. Stewart and M. Zuker
80
70
90
100
50
110
40
120
30
130
20
140
10
150
3'
5'
160
dG = -45.1 PC-53

PC-53

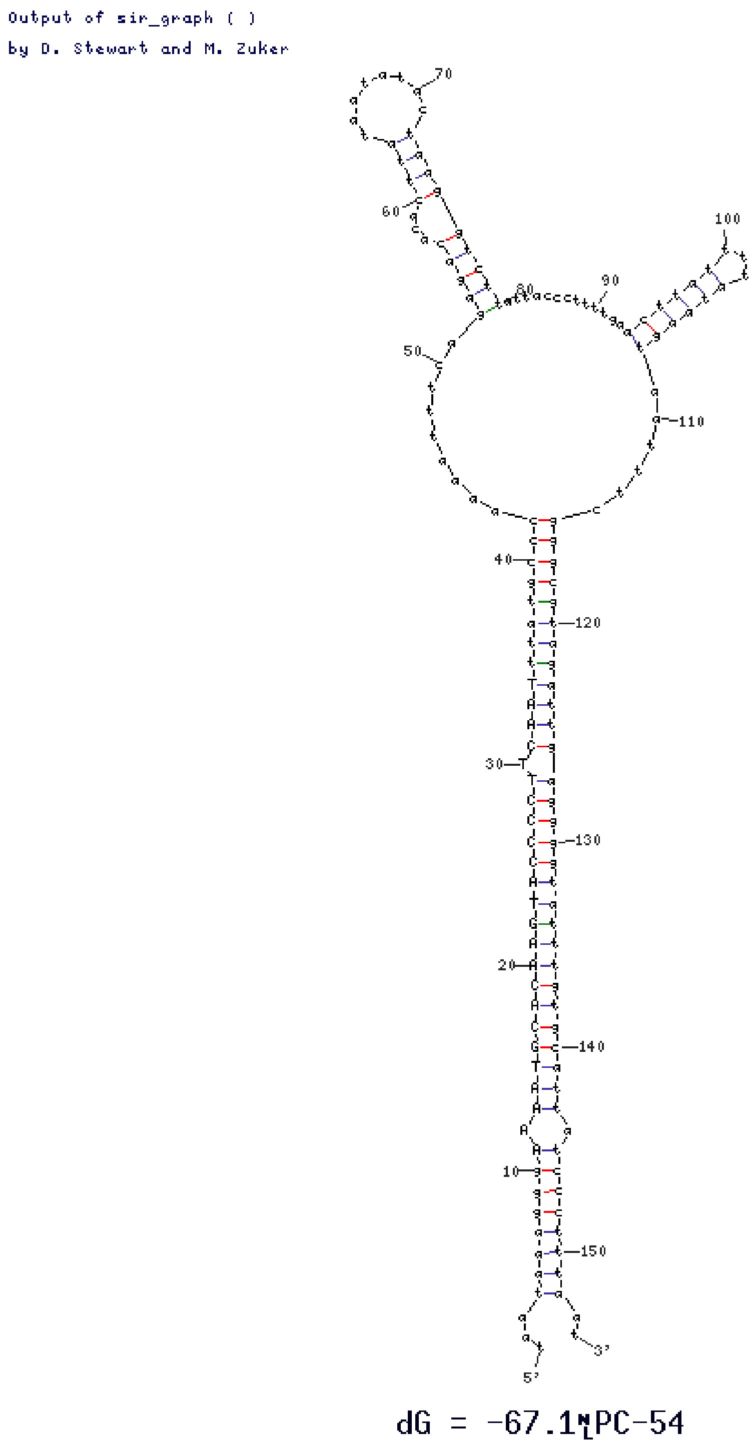

PC-54

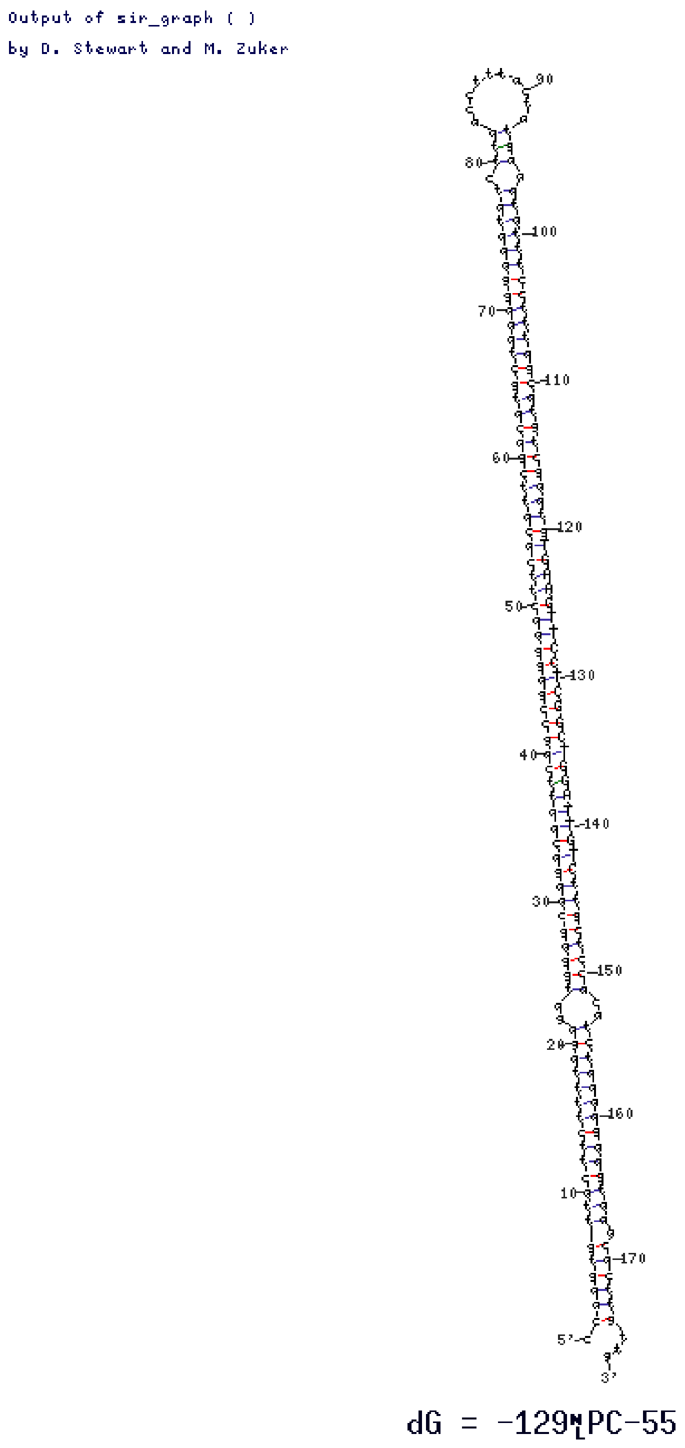
Output of sir_graph ( )
by D. Stewart and M. Zuker
90
80
100
70
110
60
120
50
130
40
140
30
150
20
160
10
170
5'
3'
PC-55

PC-55

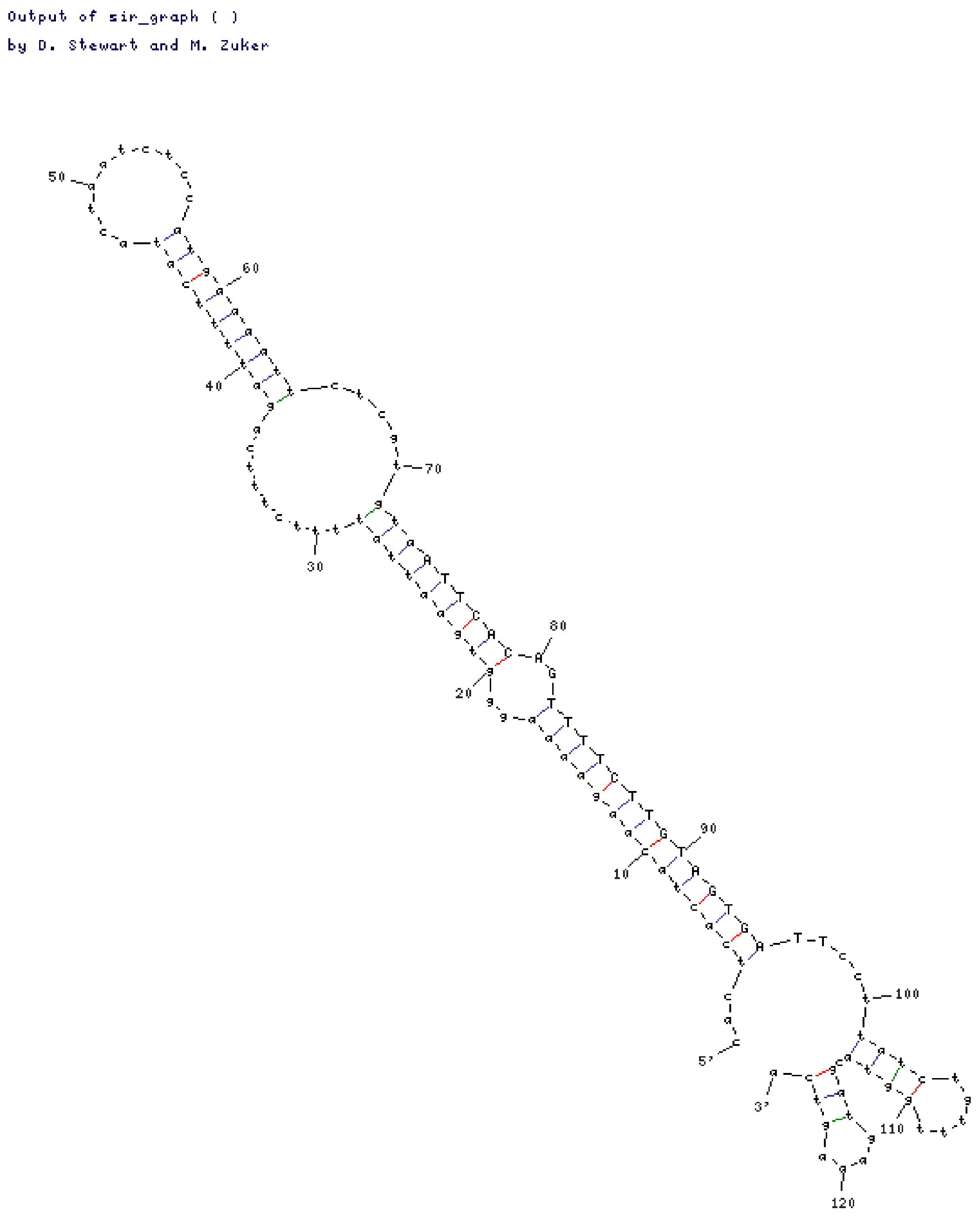

dG = -37.2[illegible]PC-56

PC-56

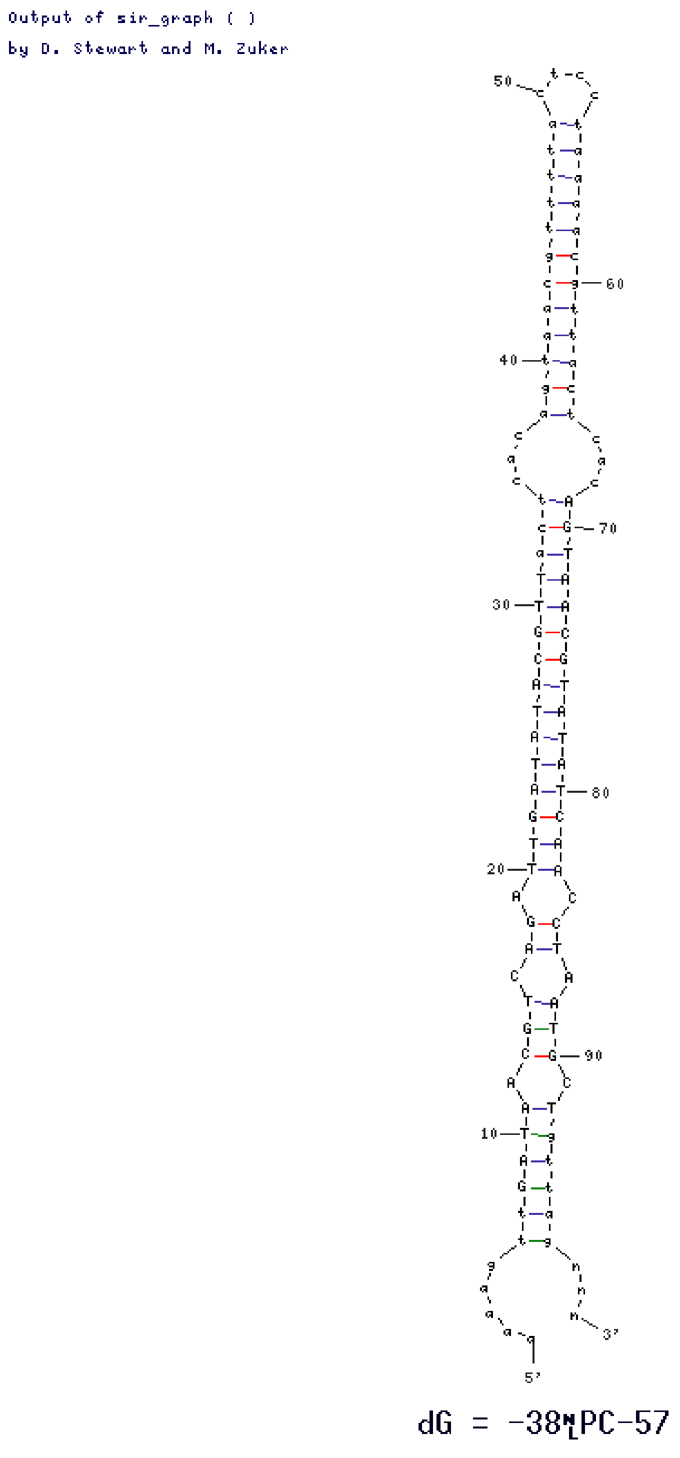

PC-57

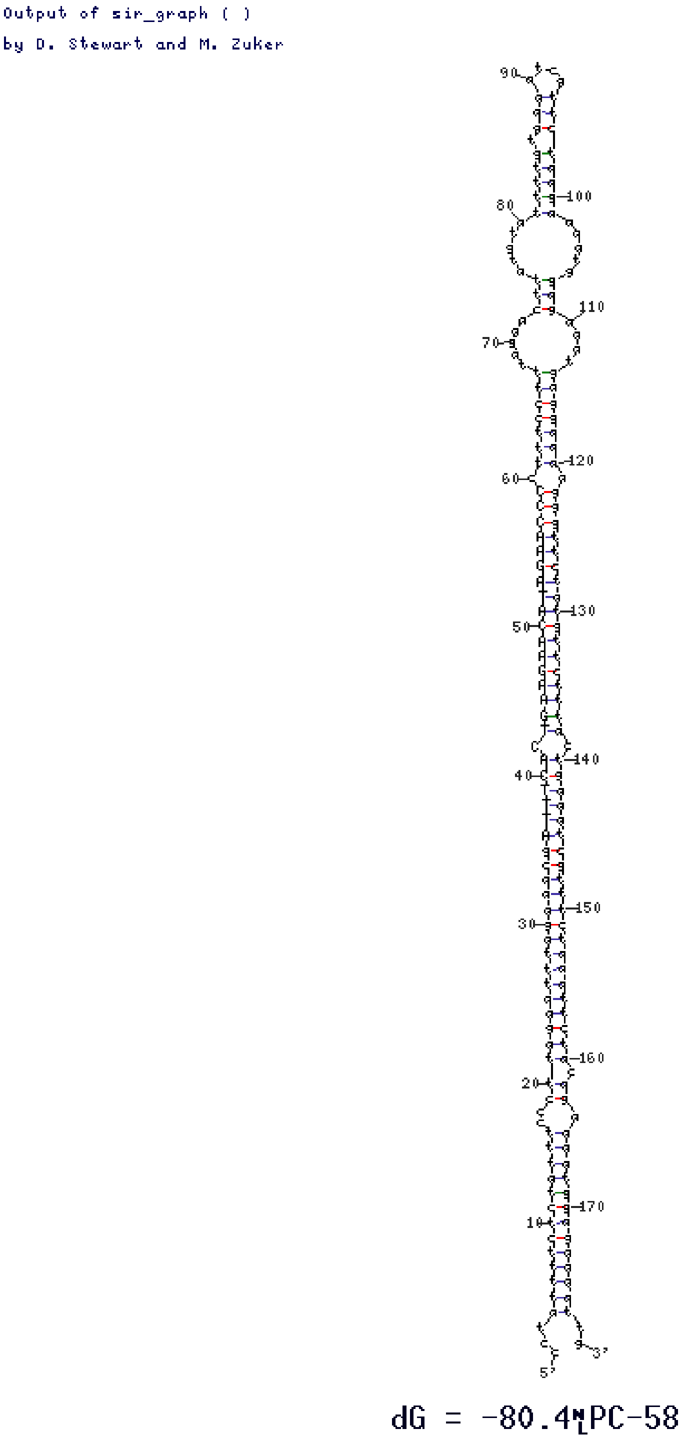

PC-58

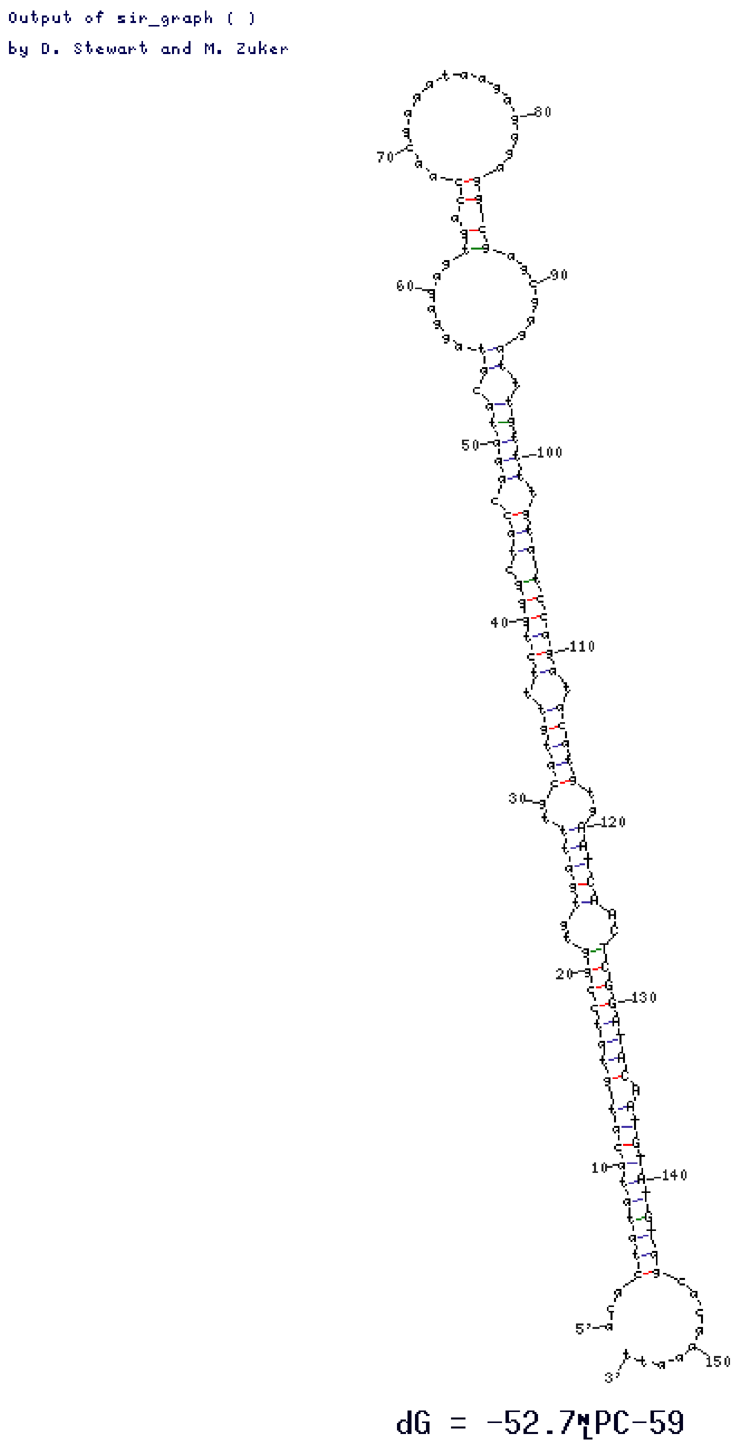

PC-59

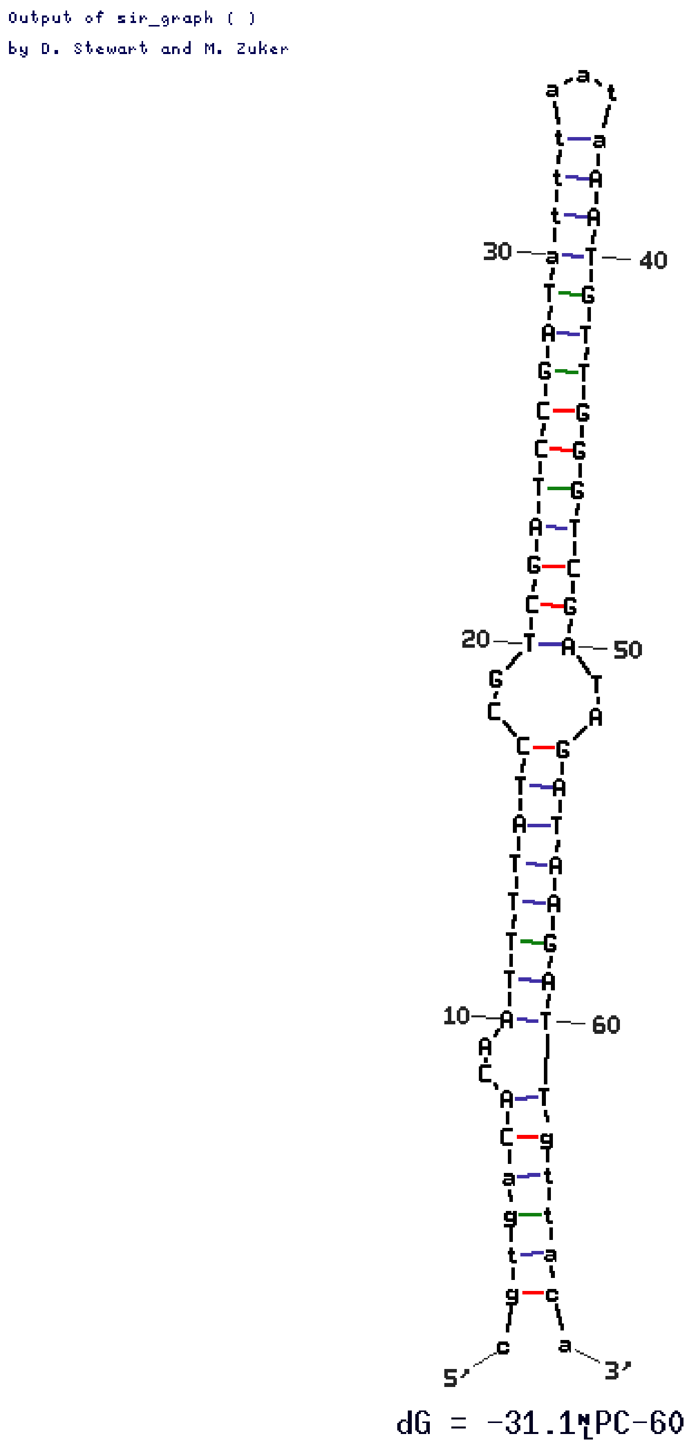
Output of sir_graph ( )
by D. Stewart and M. Zuker
30
40
20
50
10
60
5′
3′
dG = -31.1
PC-60

PC-60

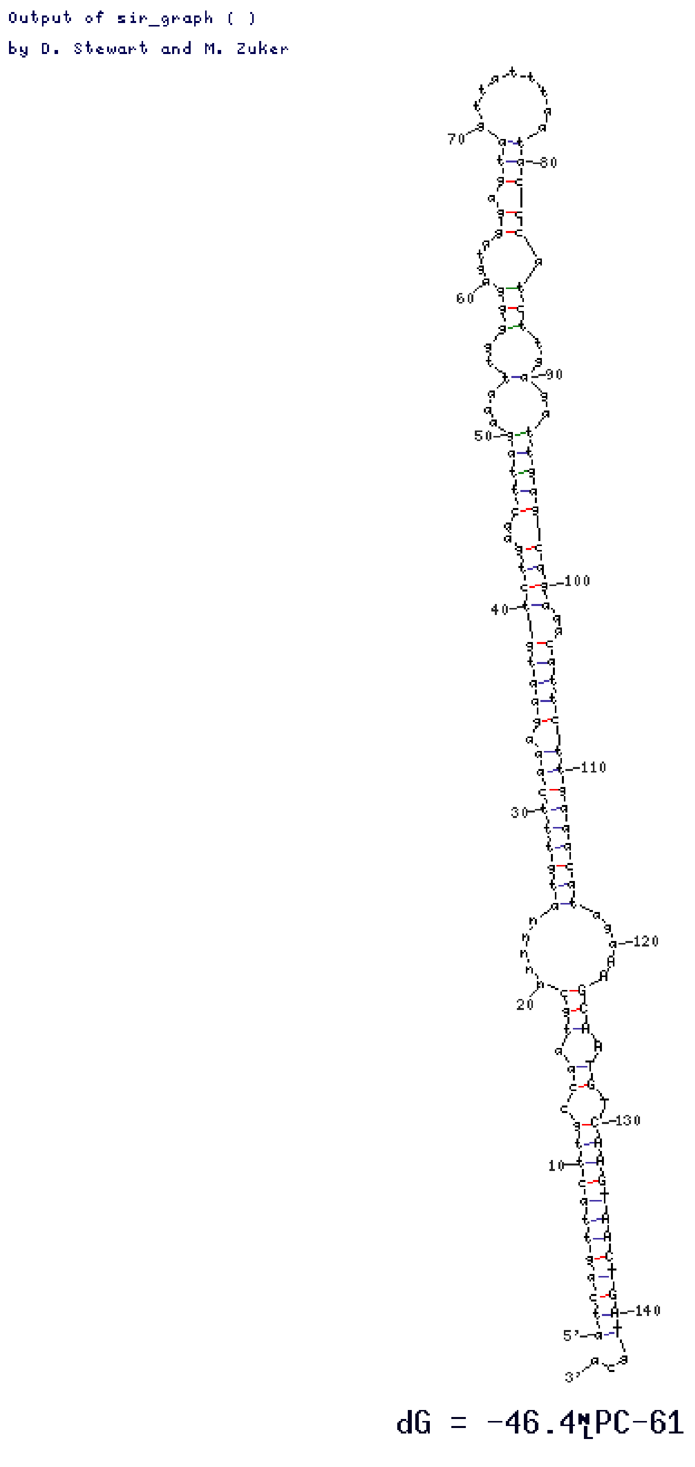

PC-61

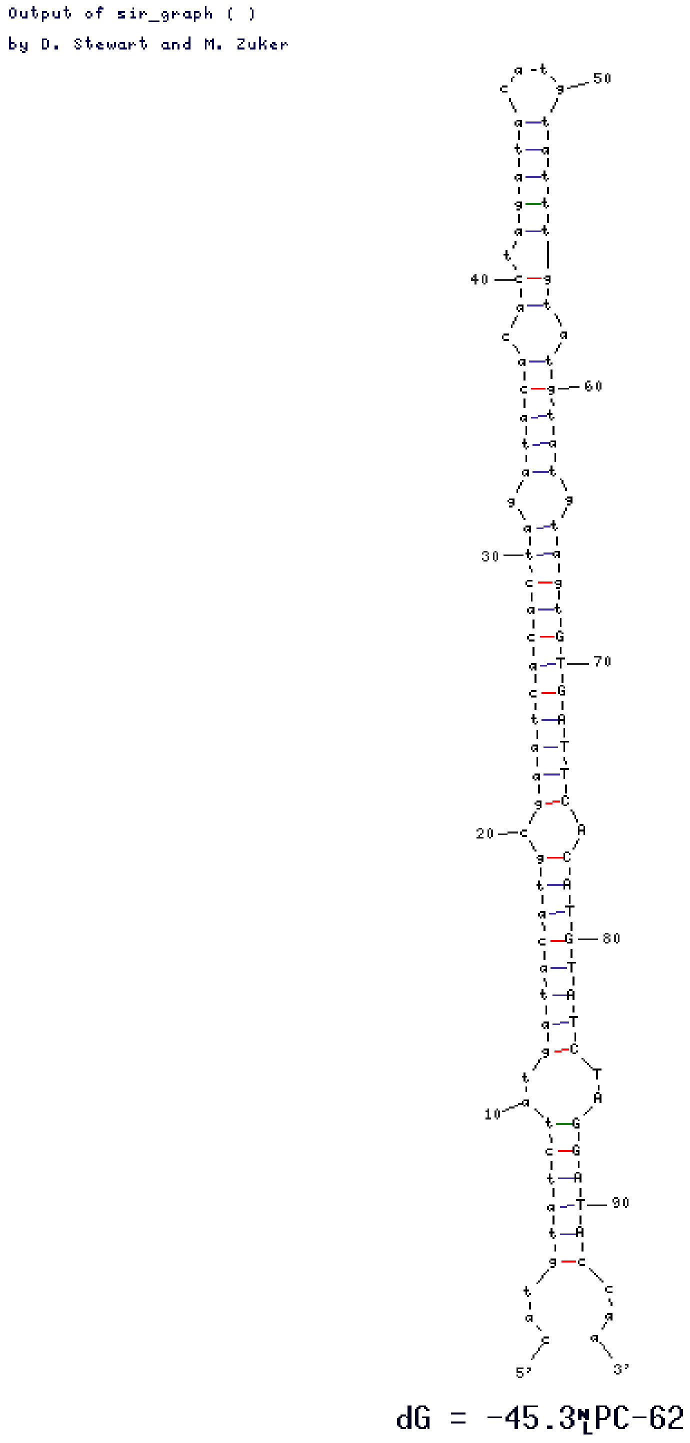

Output of sir_graph ( )
by D. Stewart and M. Zuker
50
40
60
30
70
20
80
10
90
5'
3'
dG = -45.3

PC-62

Output of sir_graph ( )
by D. Stewart and M. Zuker

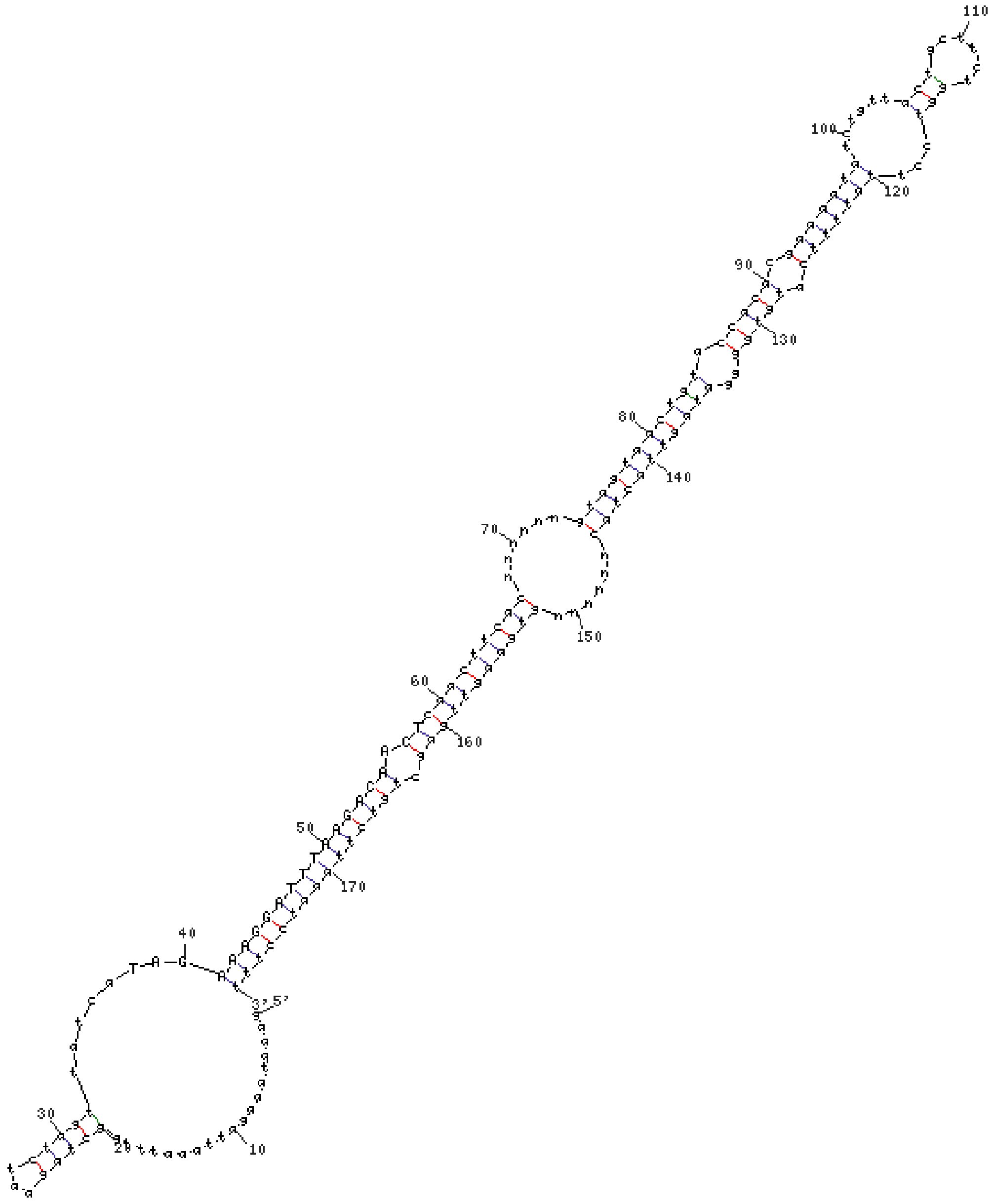

dG = -68.5 PC-63

PC-63

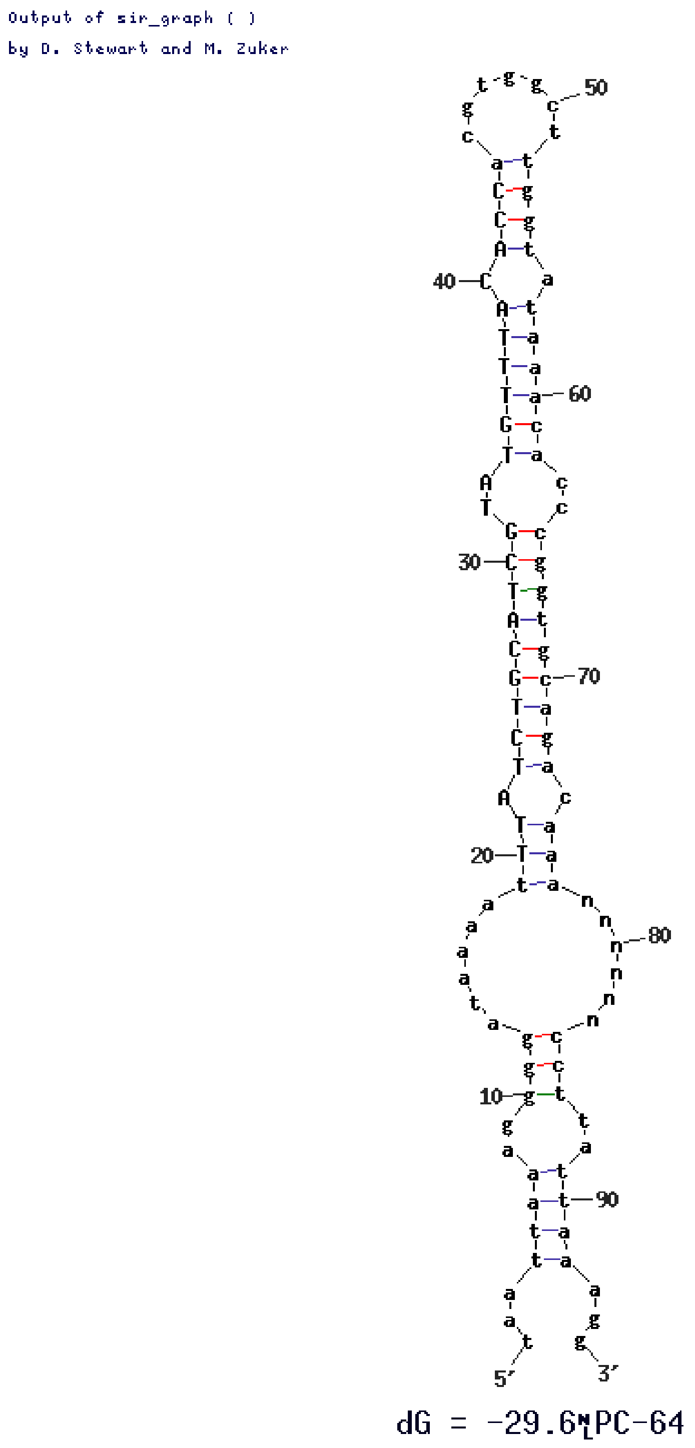

PC-64

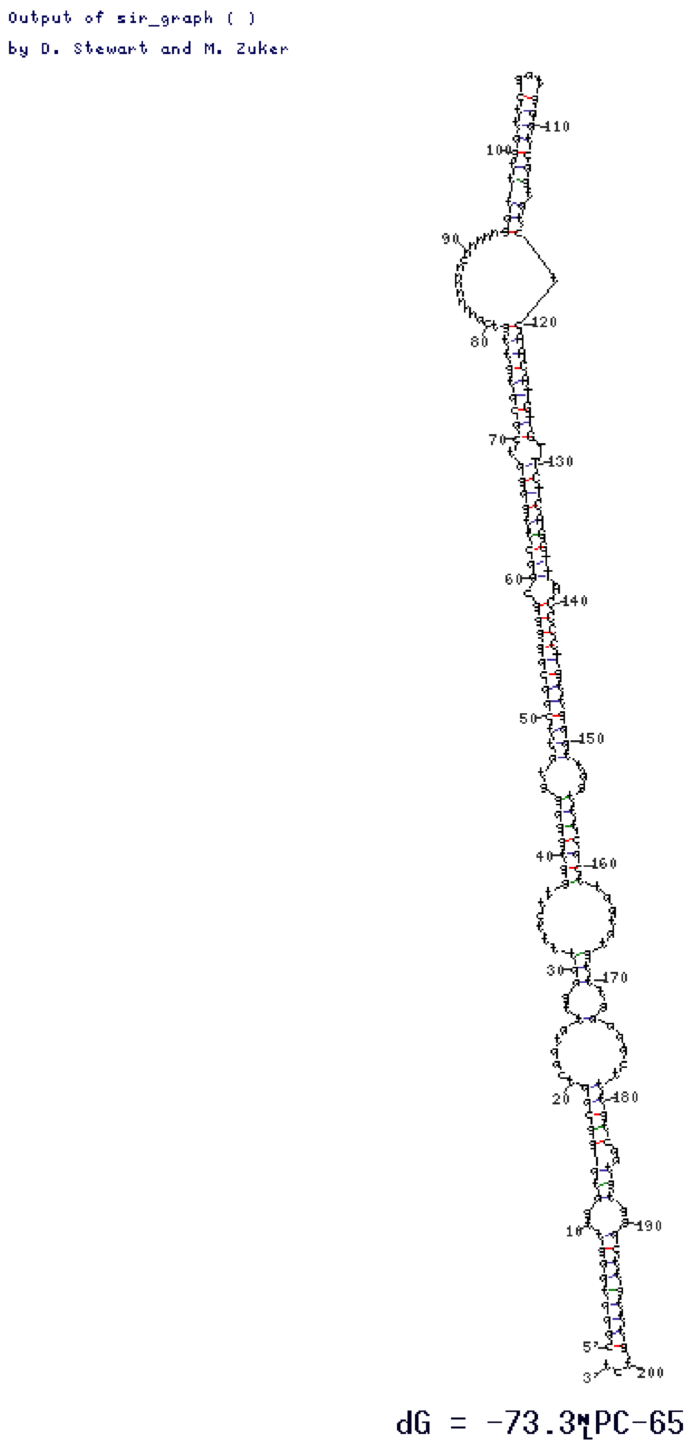

PC-65

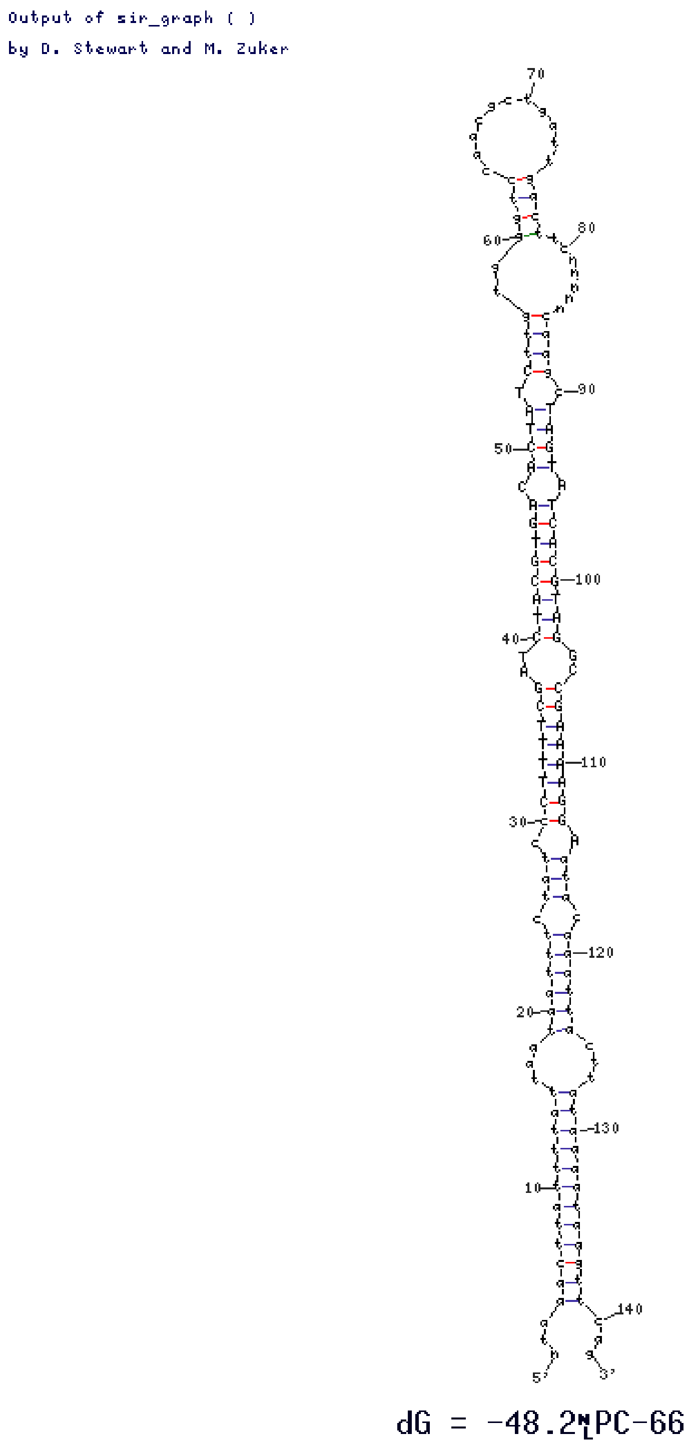

PC-66

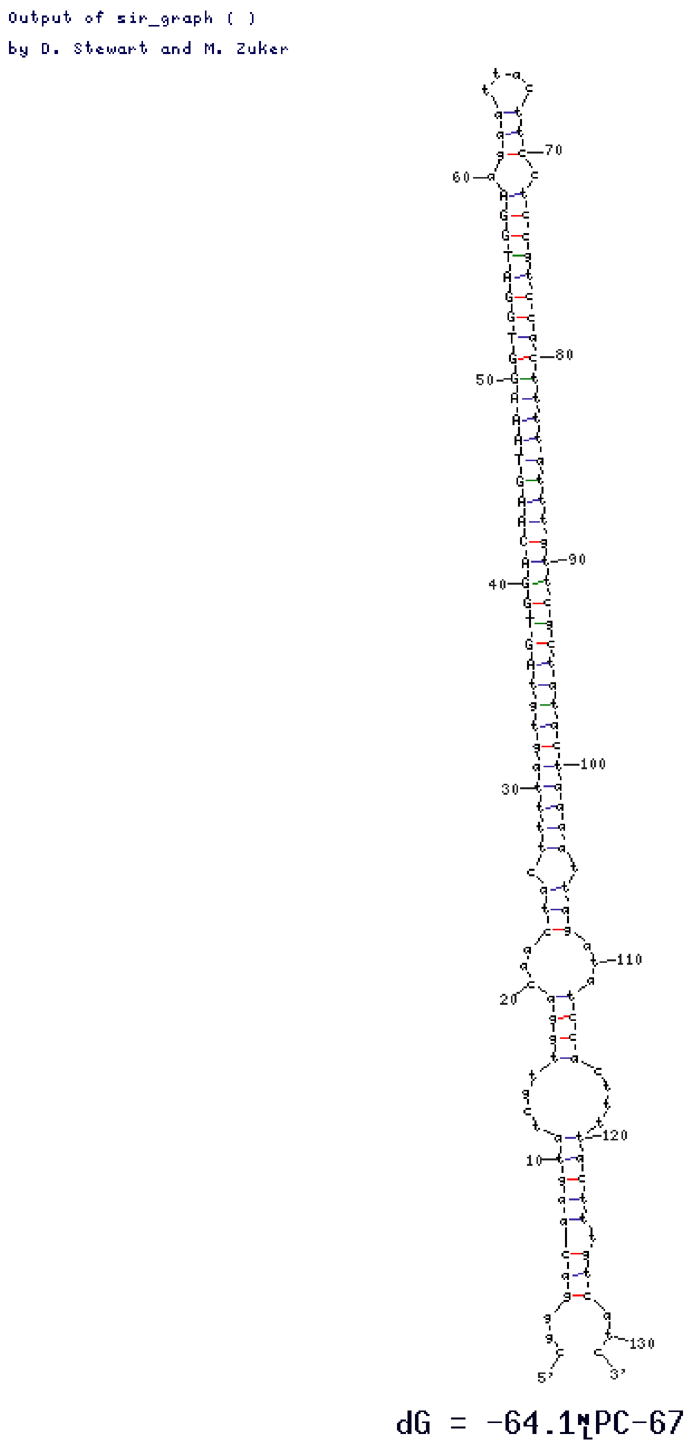

PC-67

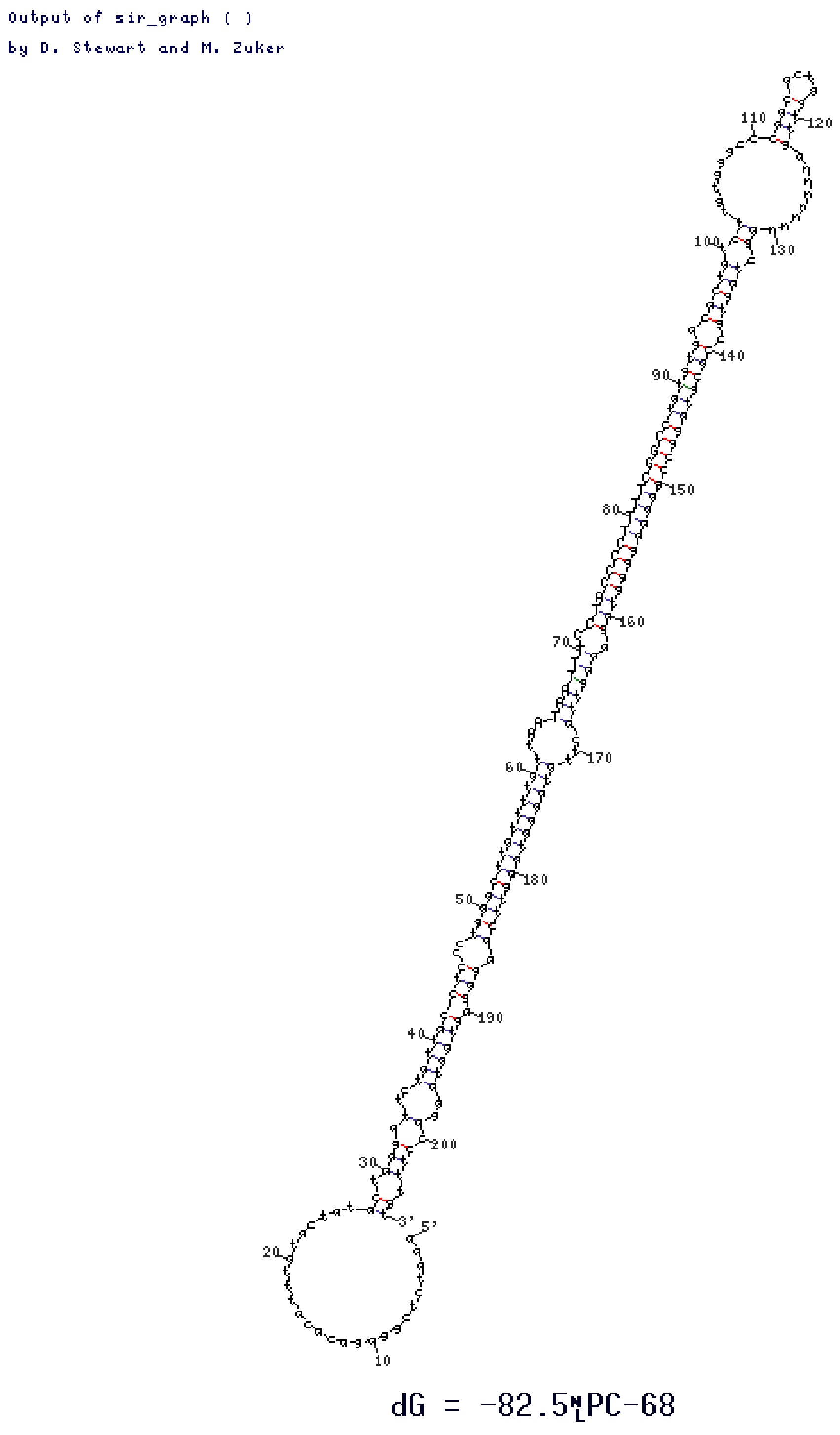

PC-68

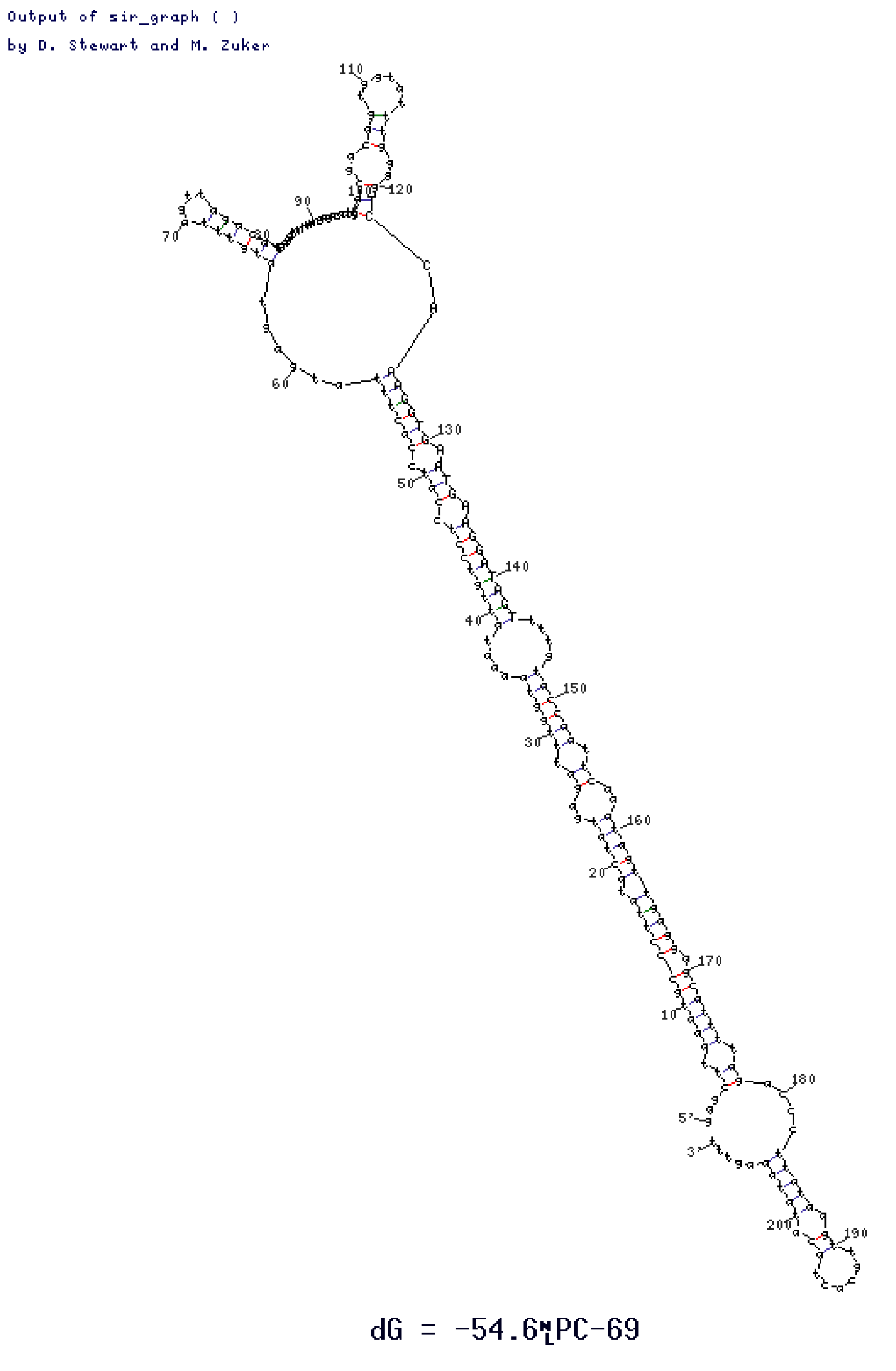

PC-69

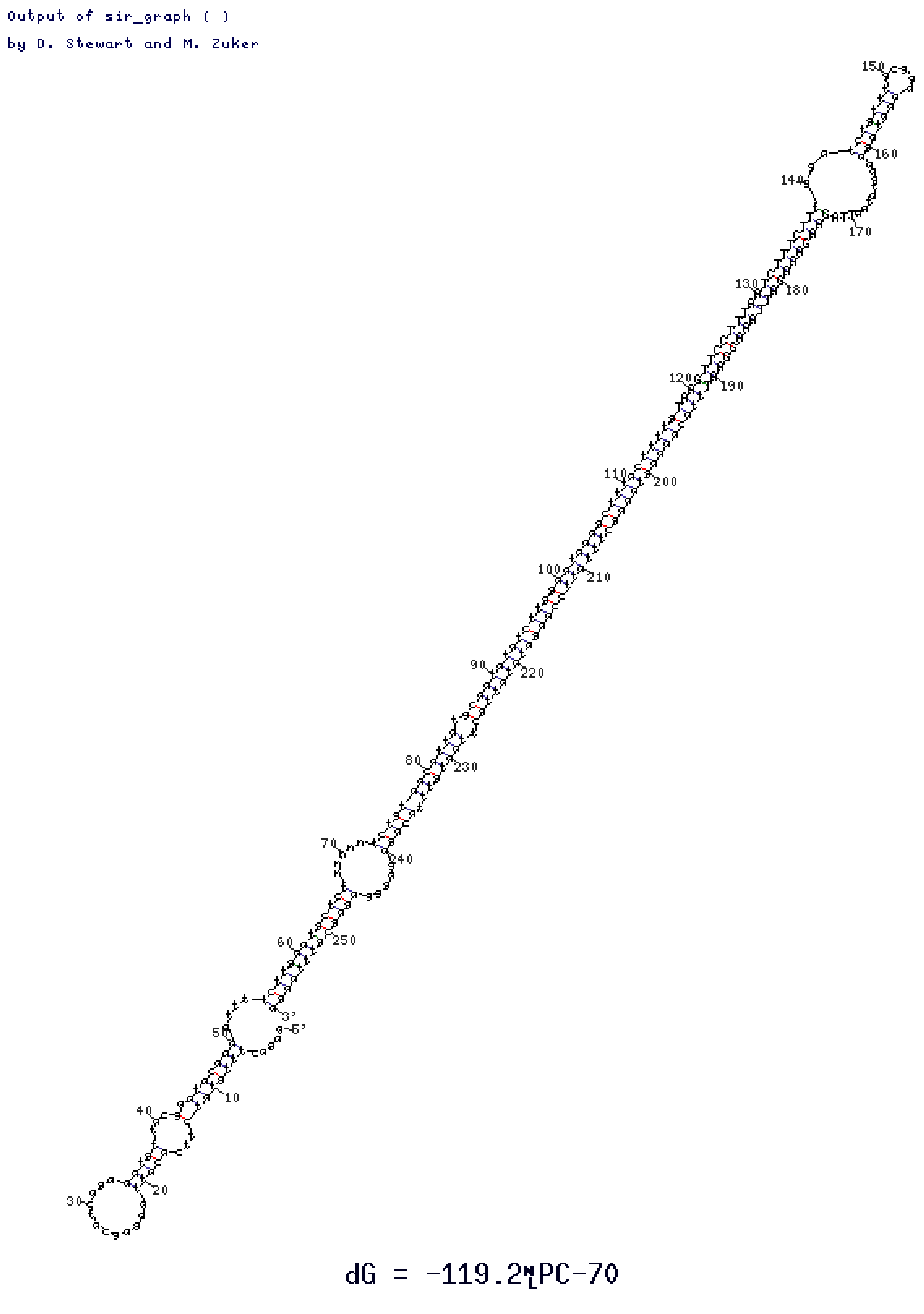

PC-70

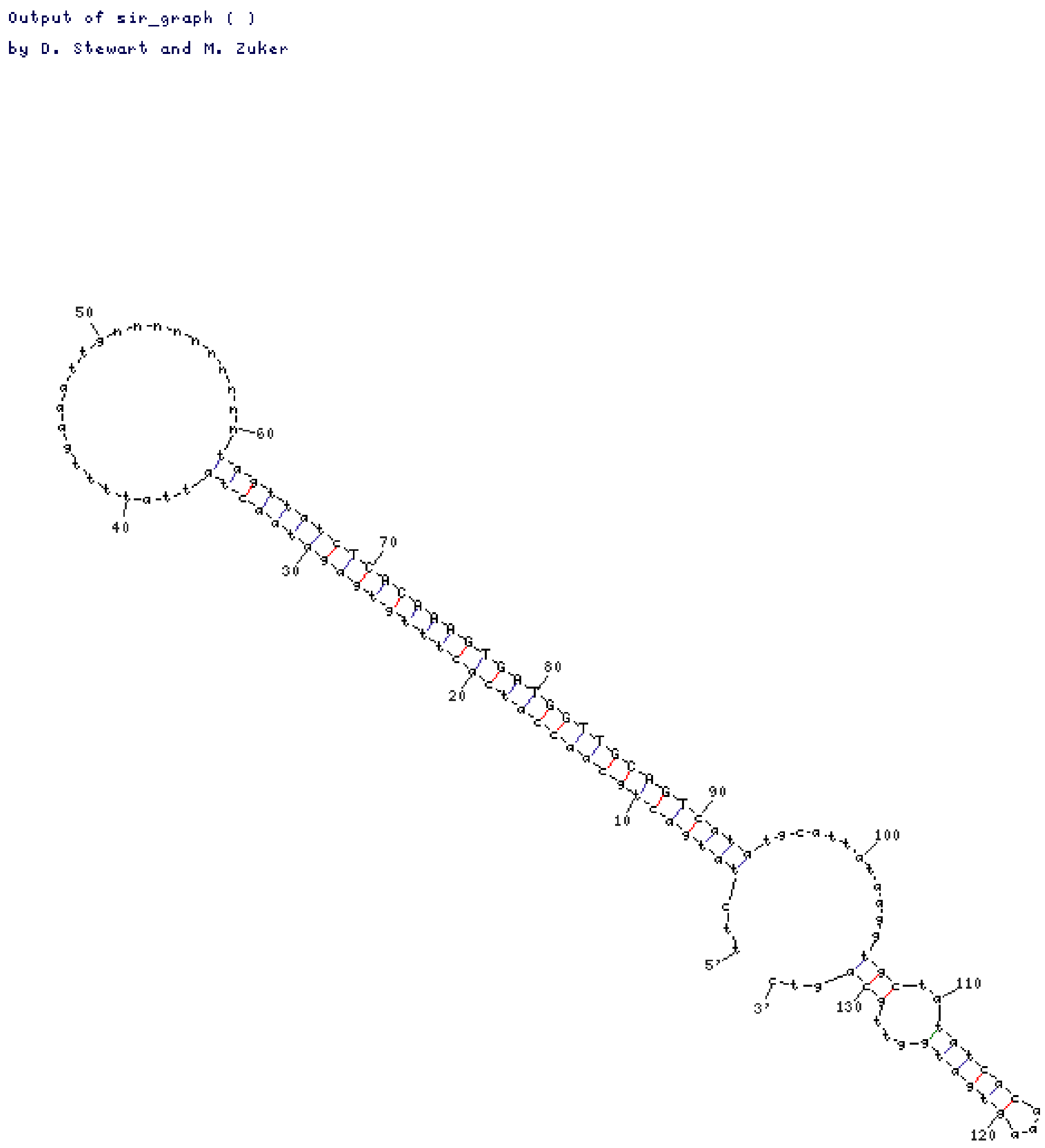

dG = -61.8 PC-71

PC-71

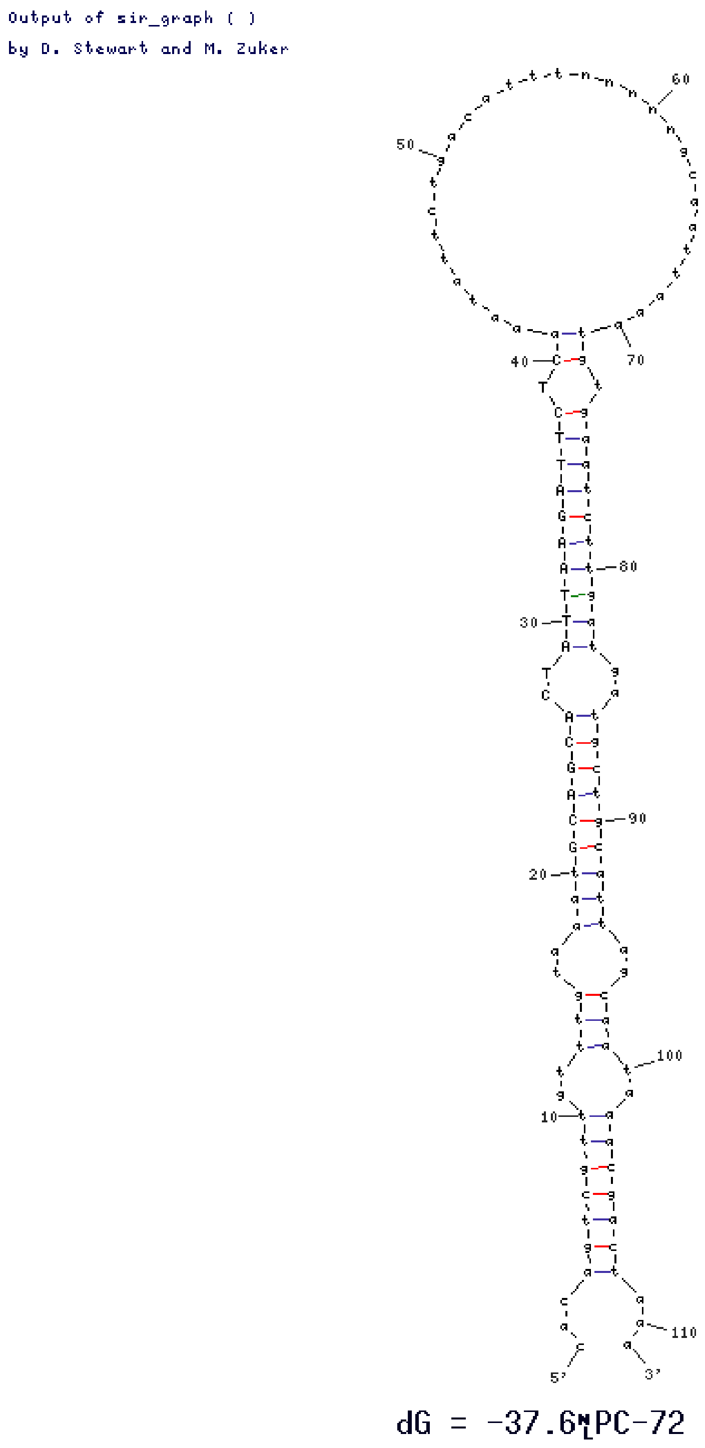
Output of sir_graph ( )
by D. Stewart and M. Zuker
50
60
40
70
80
30
90
20
100
10
110
5'
3'
dG = -37.6 PC-72

PC-72

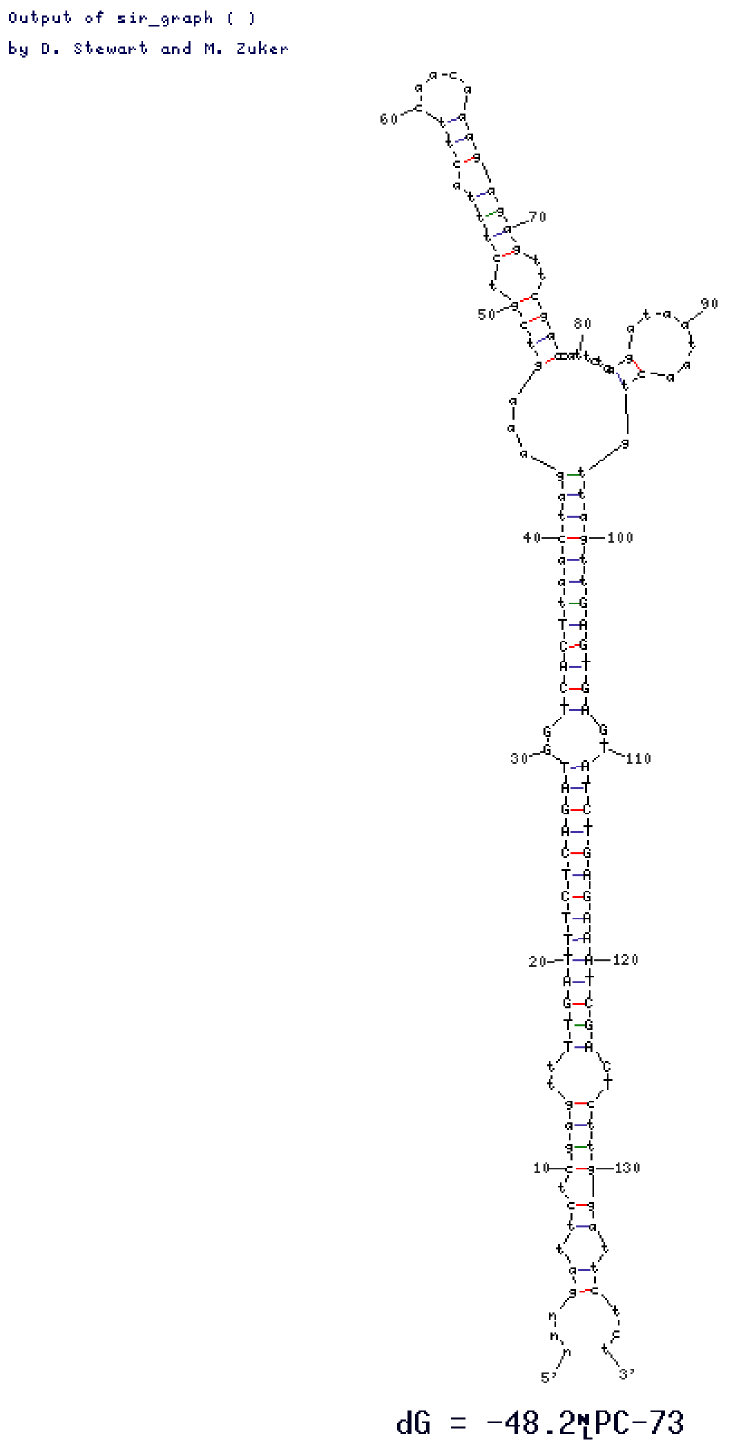

PC-73

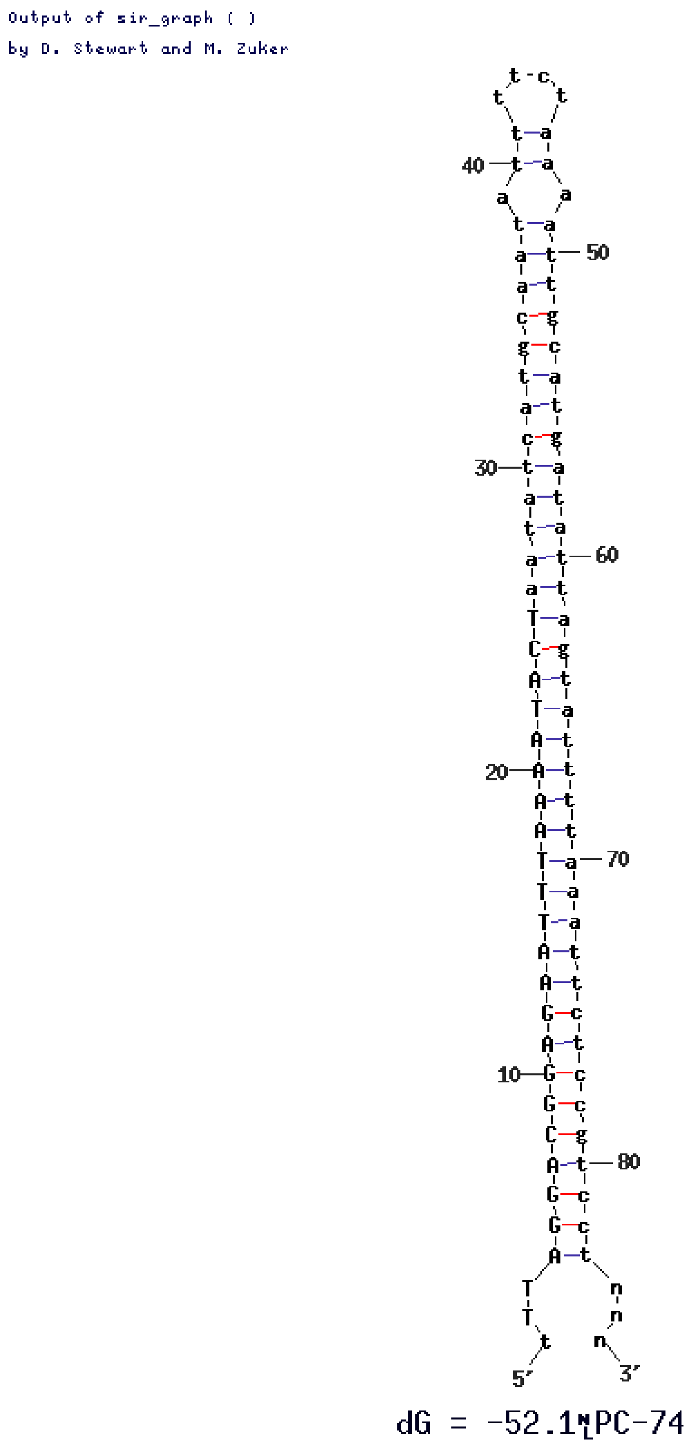
Output of sir_graph ( )
by D. Stewart and M. Zuker
40
50
30
60
20
70
10
80
5′
3′
dG = -52.1 PC-74

PC-74

Output of sir_graph ( )
by D. Stewart and M. Zuker

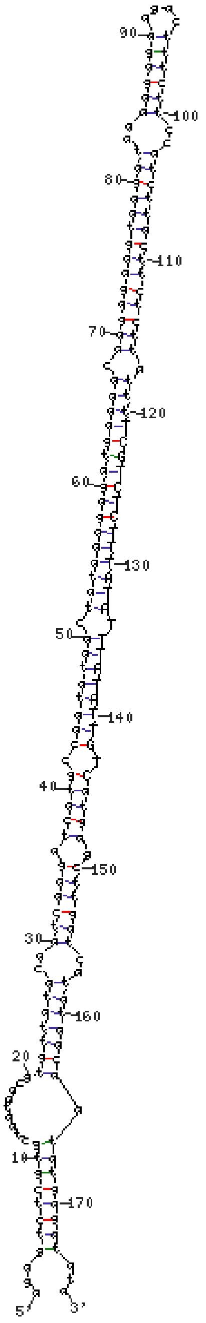

dG = -77.8 PC-75

PC-75

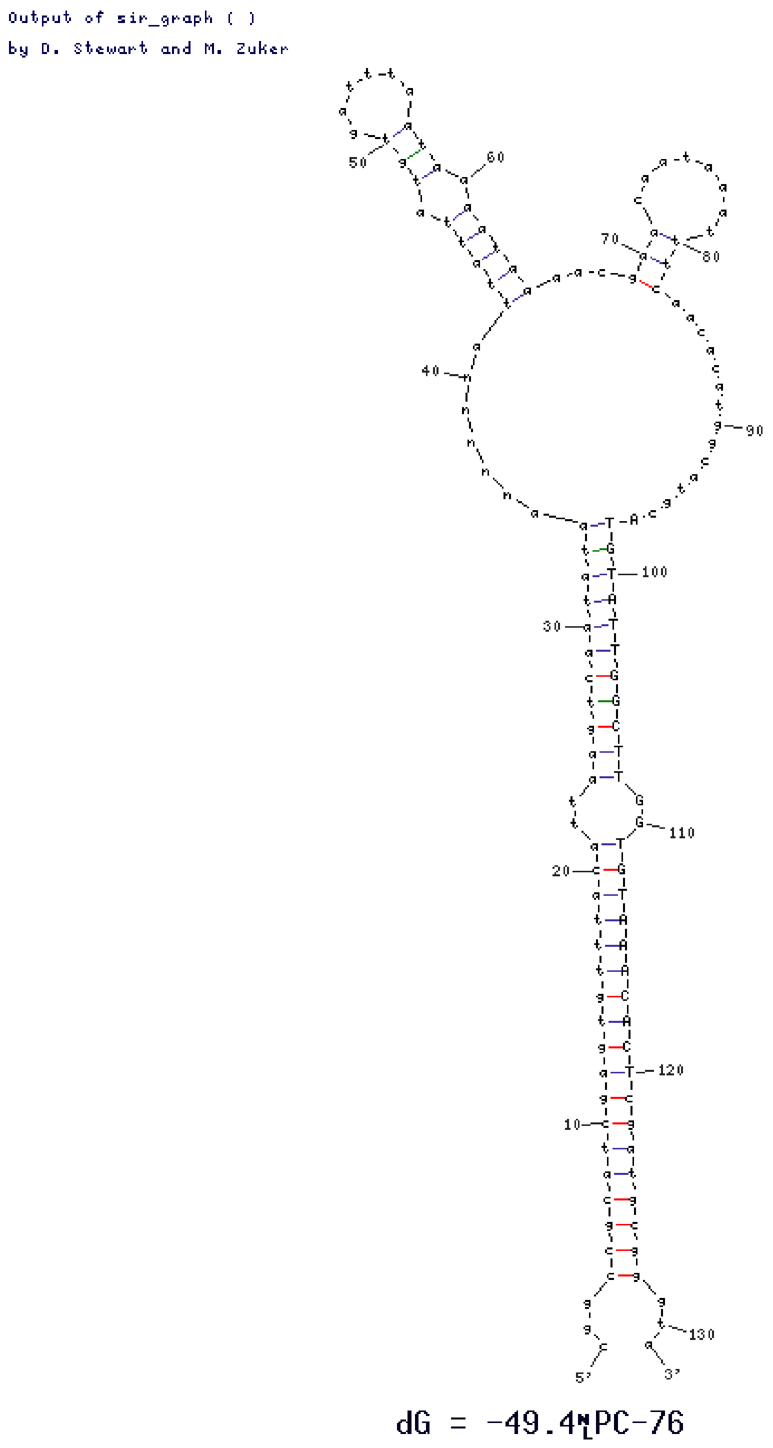

PC-76

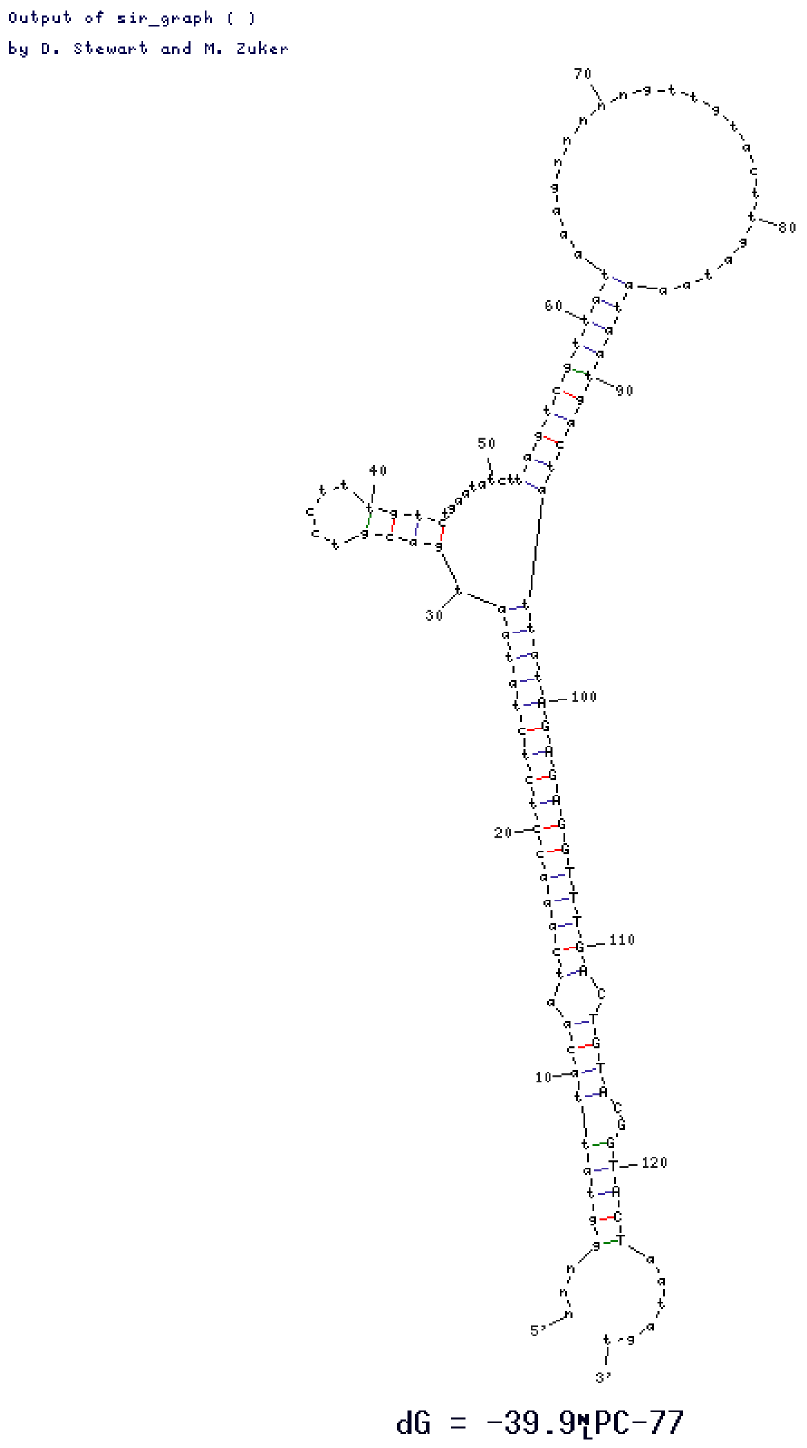

PC-77

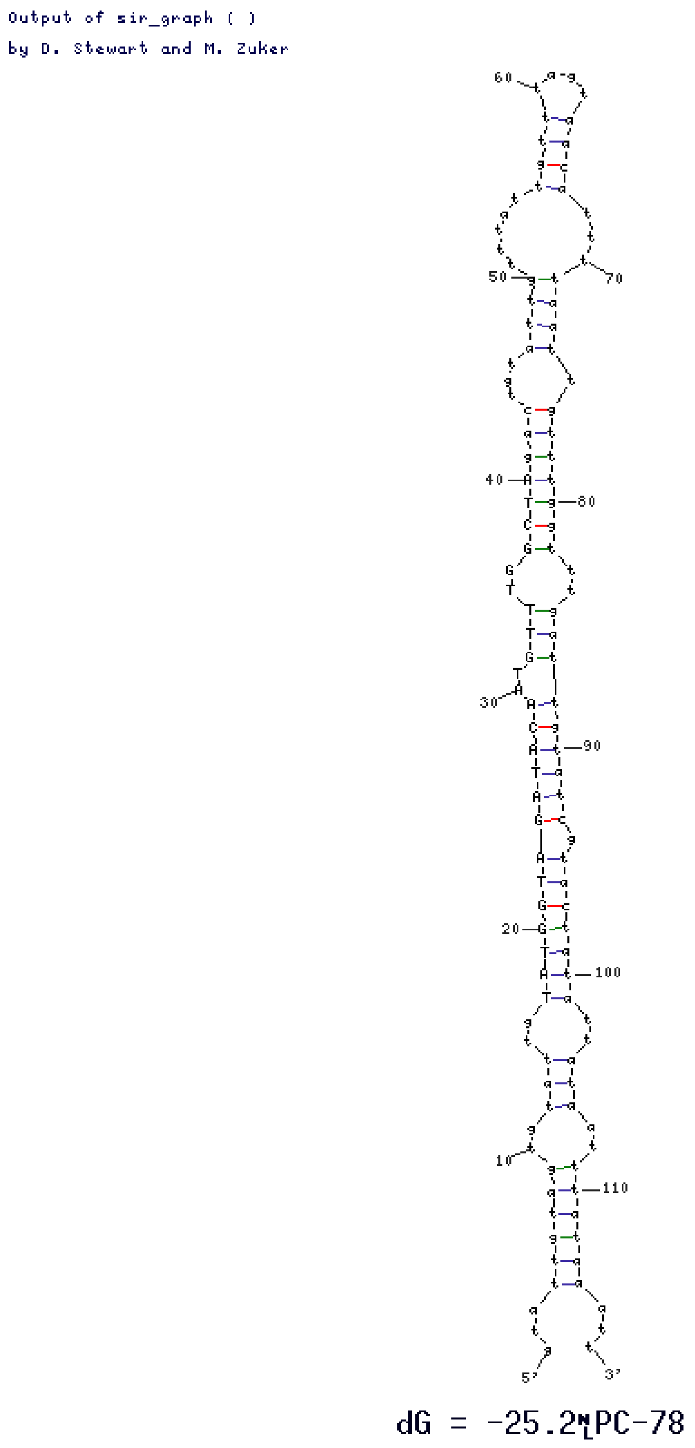
Output of sir_graph ( )
by D. Stewart and M. Zuker
60
50
70
40
80
30
90
20
100
10
110
5'
3'
dG = -25.2
PC-78

PC-78

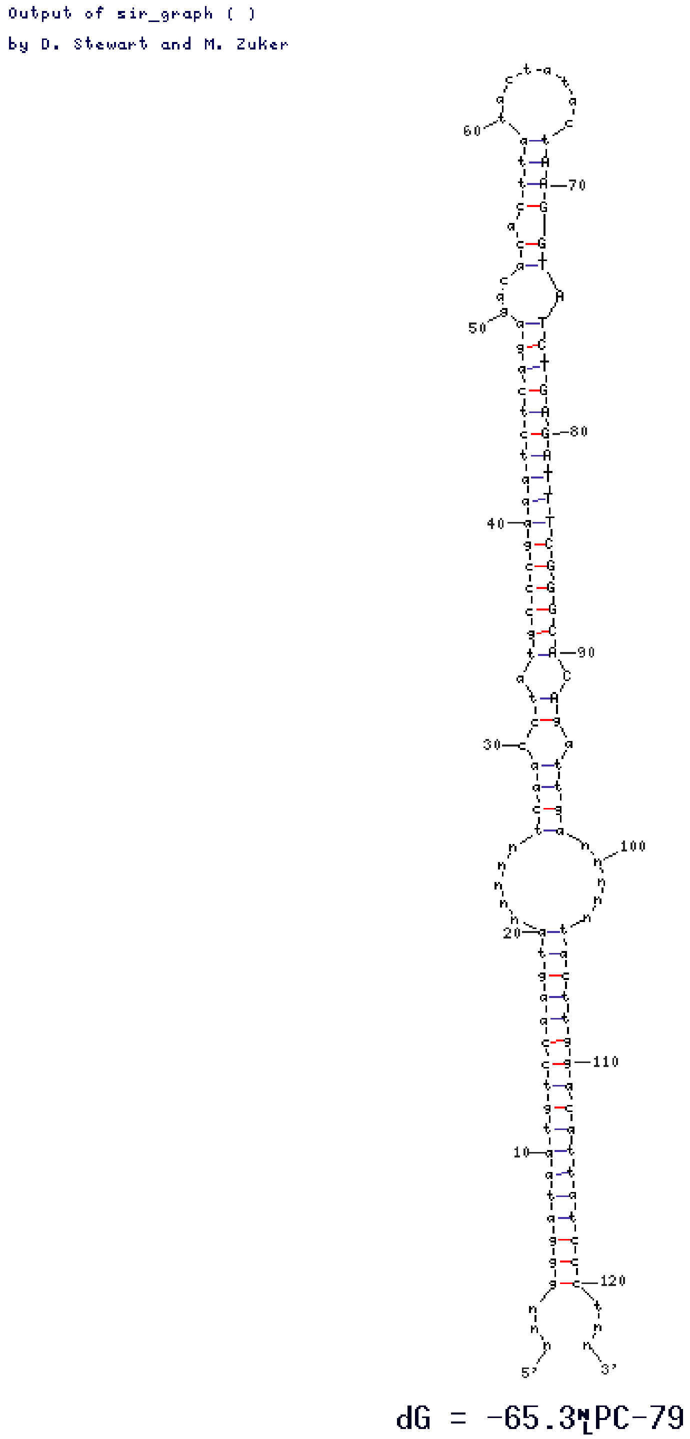

PC-79

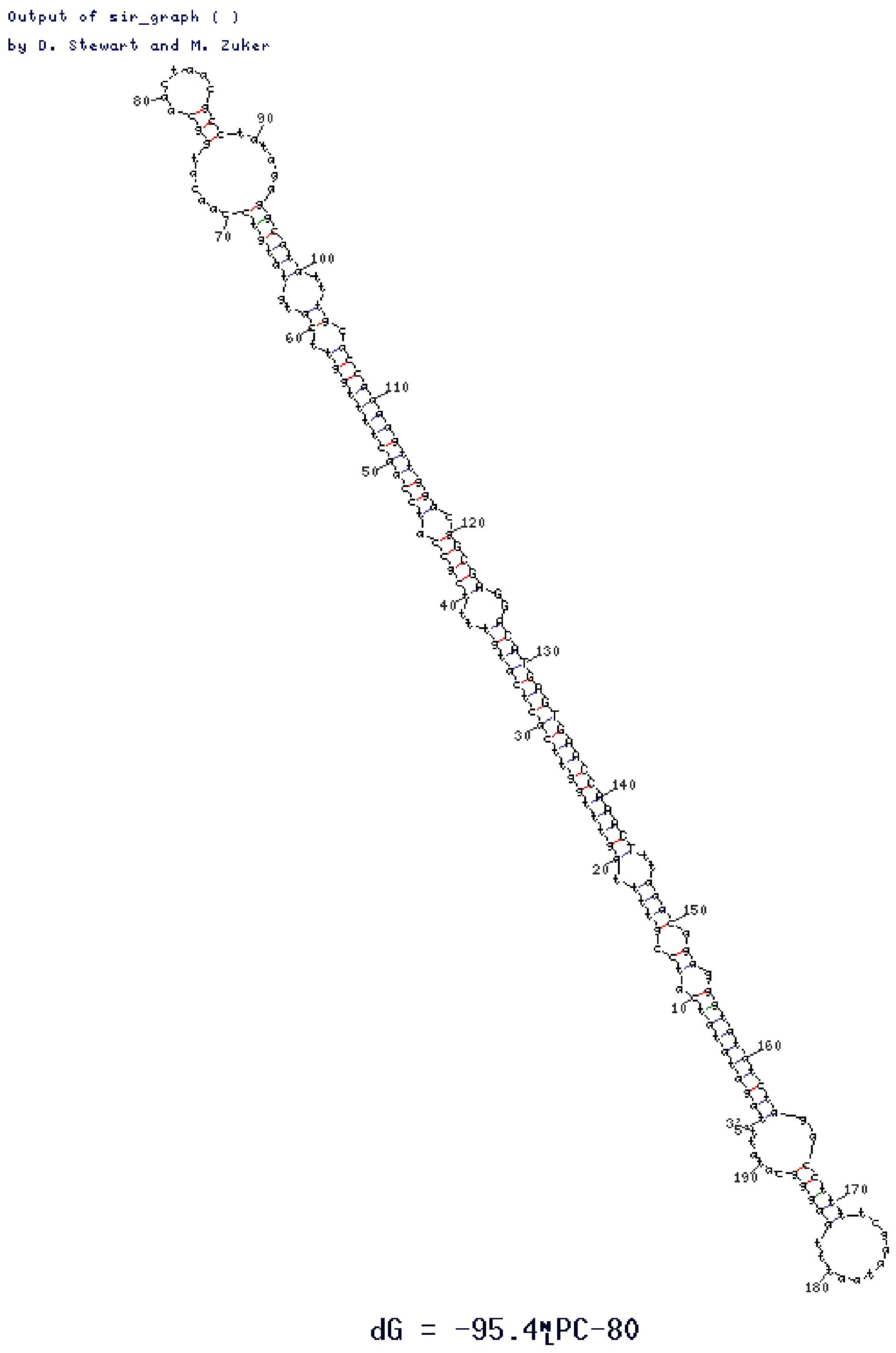

PC-80

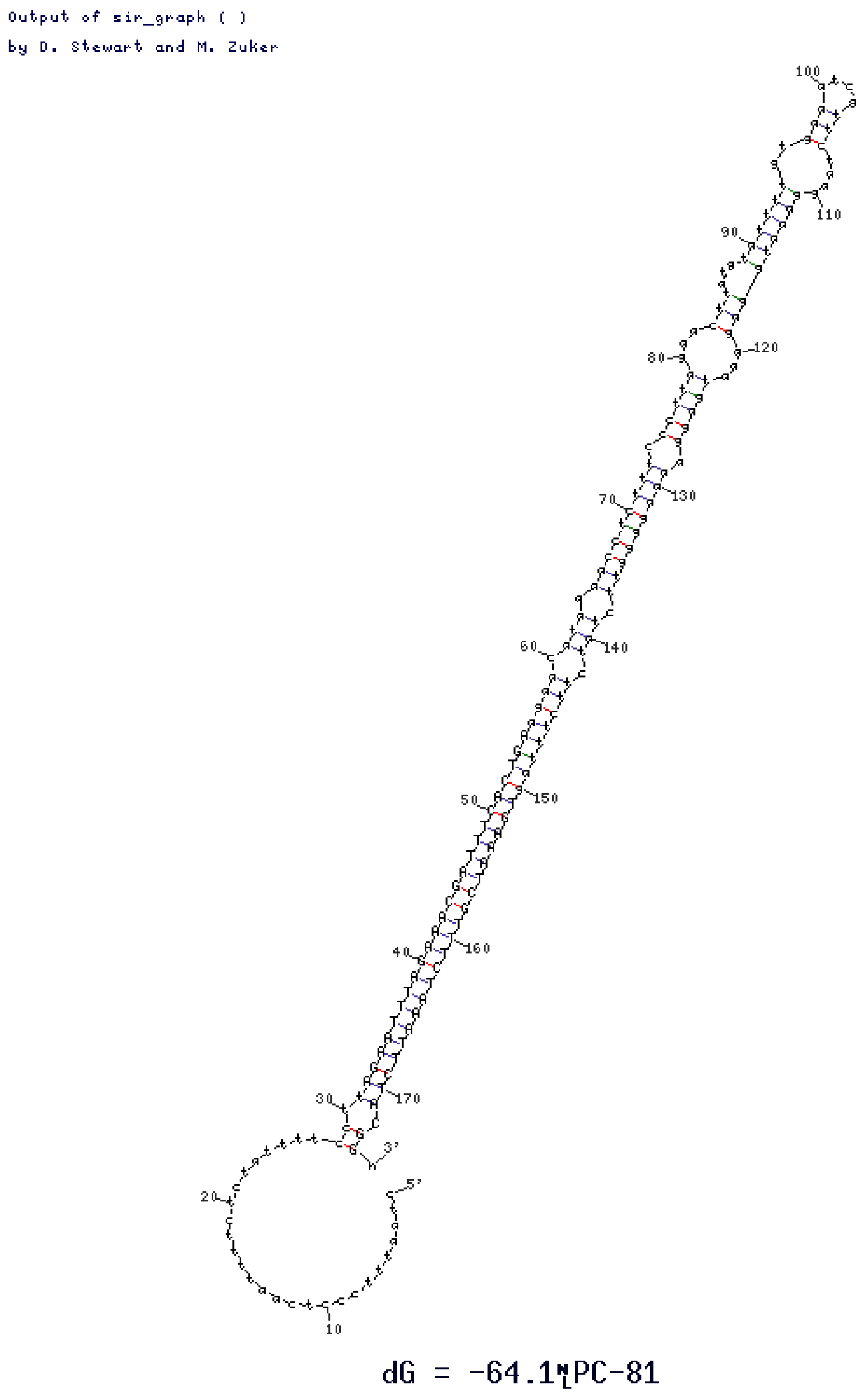
Output of sir_graph ( )
by D. Stewart and M. Zuker
100
110
90
120
80
130
70
140
60
150
50
160
40
170
30
3'
5'
20
10
dG = -64.1
PC-81

PC-81

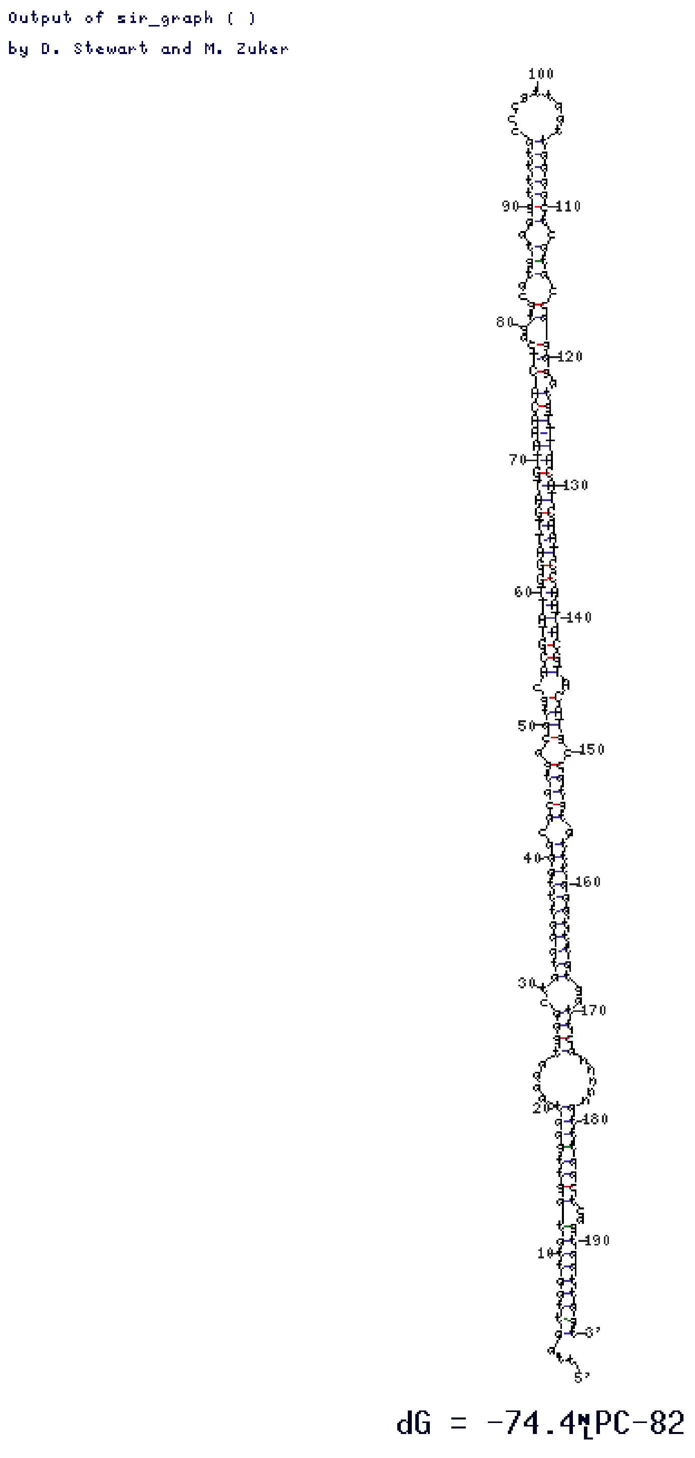

PC-82

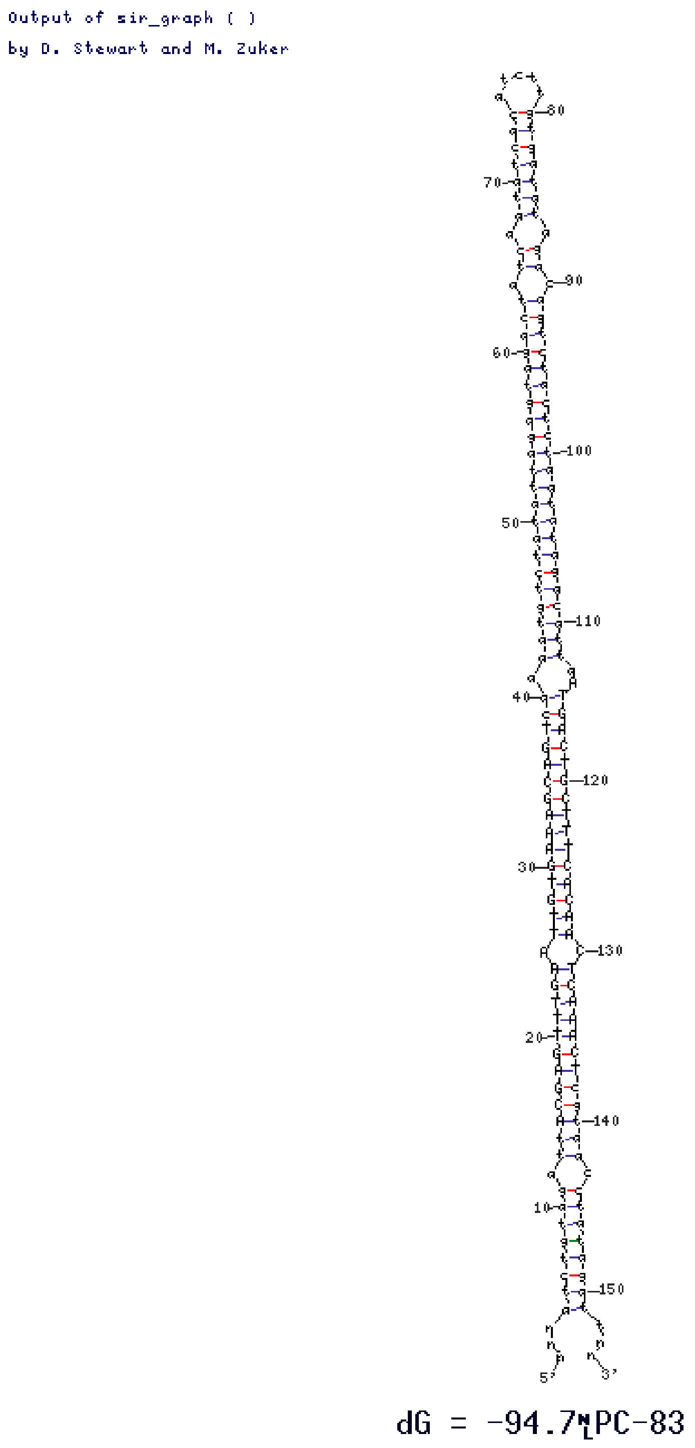

PC-83

Output of sir_graph ( )
by D. Stewart and M. Zuker

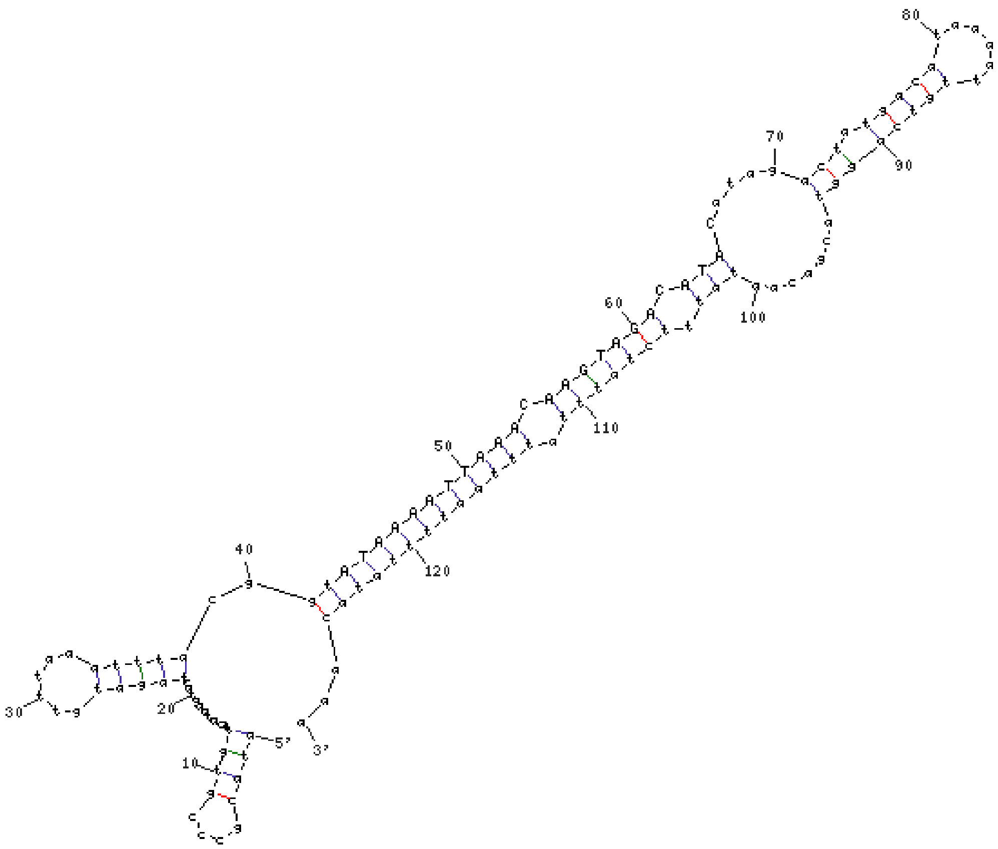

dG = -25.8 PC-84

PC-84

Output of sir_graph ( )
by D. Stewart and M. Zuker

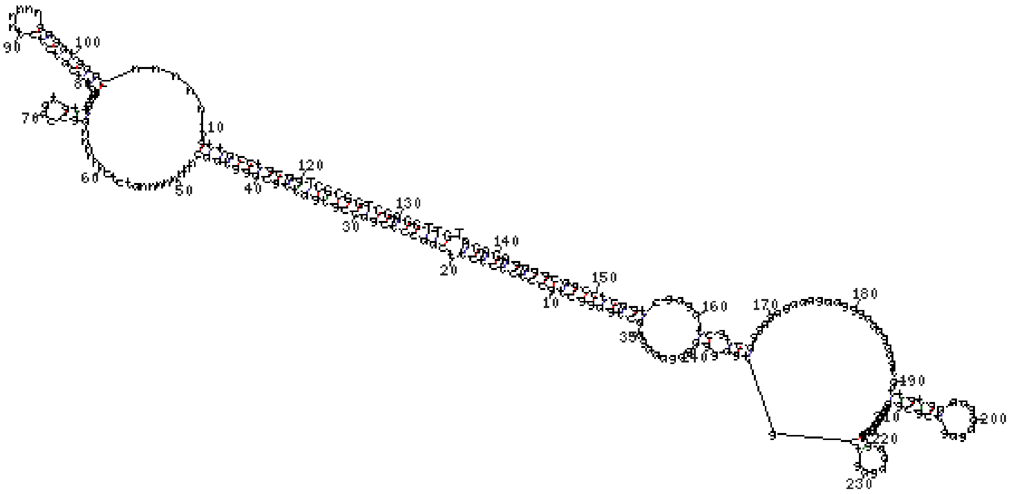

dG = -108.1 PC-85

PC-85

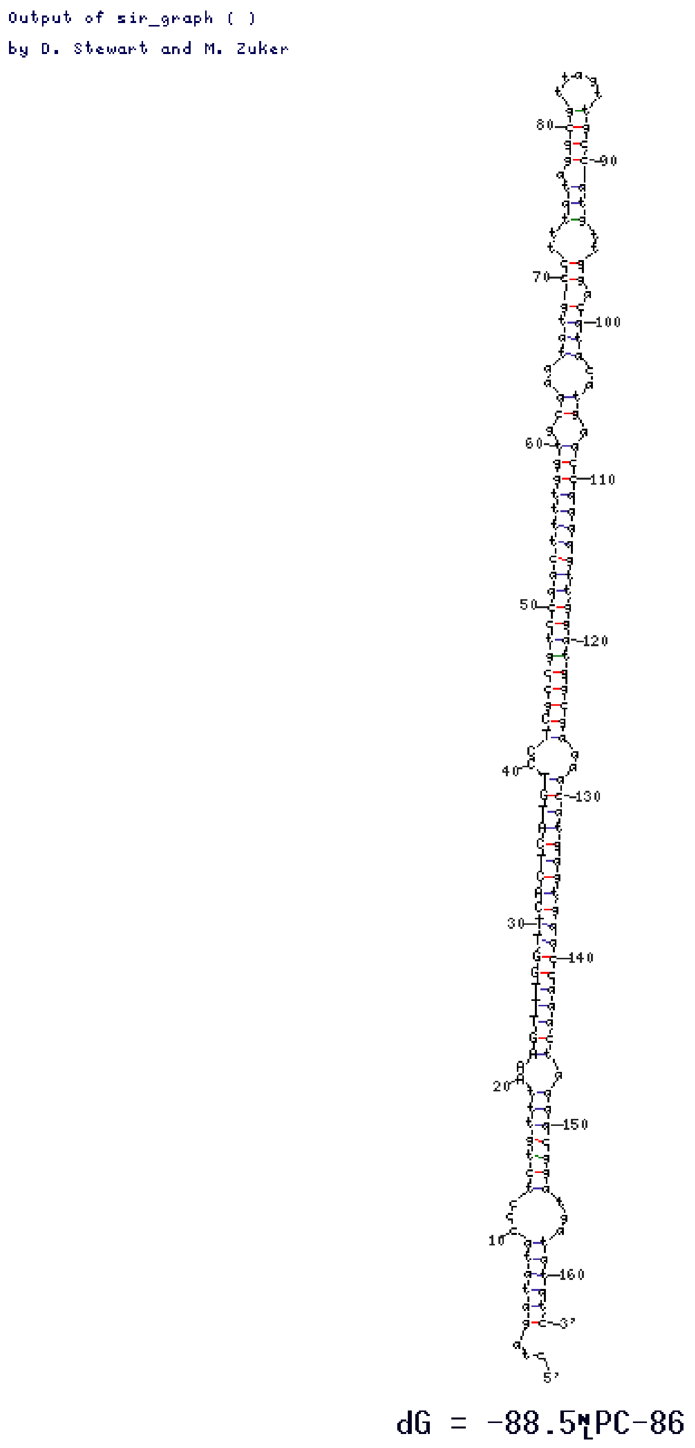
Output of sir_graph ( )
by D. Stewart and M. Zuker
80
90
70
100
60
110
50
120
40
130
30
140
20
150
10
160
3'
5'
dG = -88.5 PC-86

PC-86

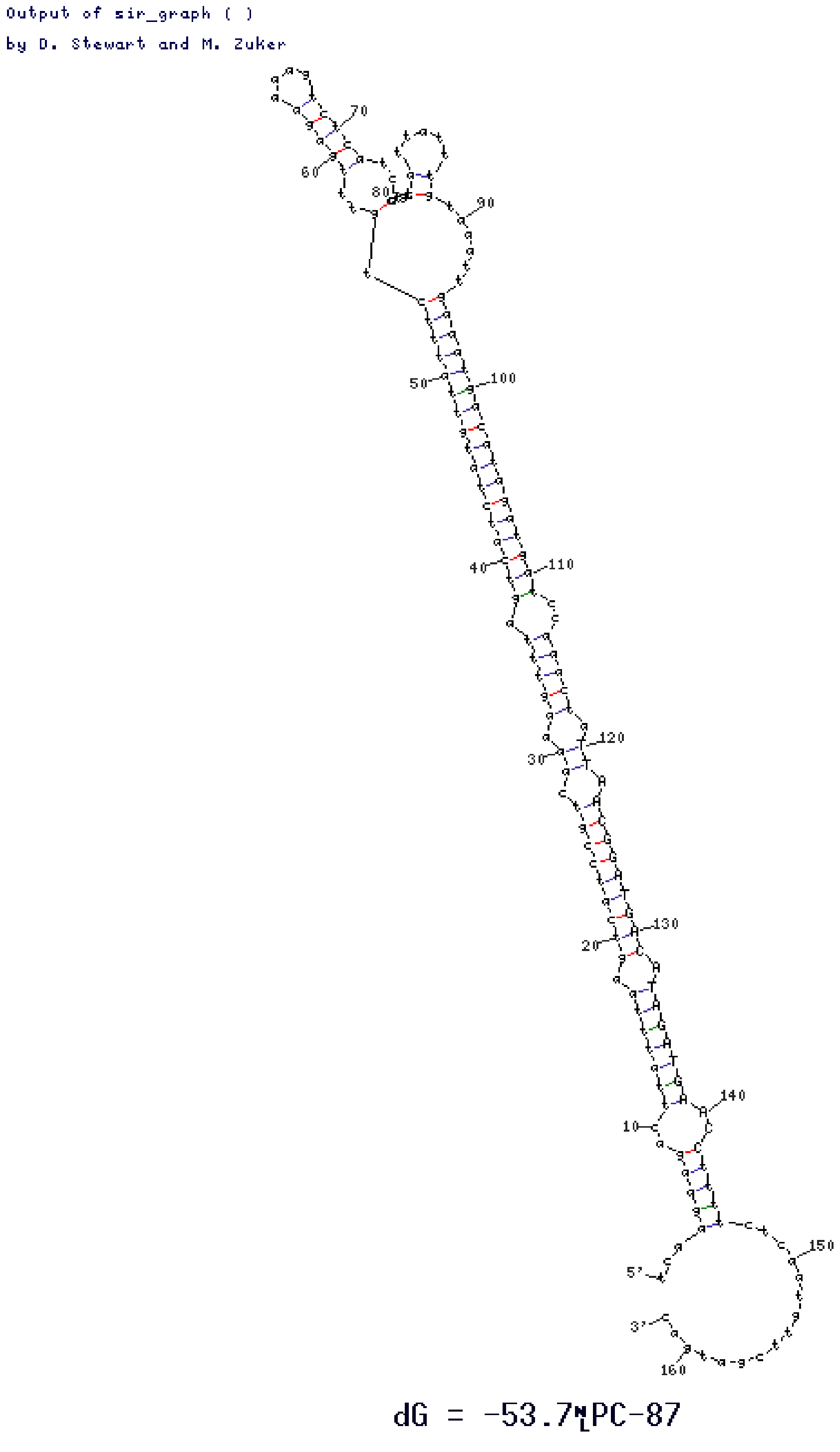
Output of sir_graph ( )
by D. Stewart and M. Zuker
70
60
80
90
50
100
40
110
30
120
20
130
10
140
150
5'
3'
160
dG = -53.7
PC-87

PC-87

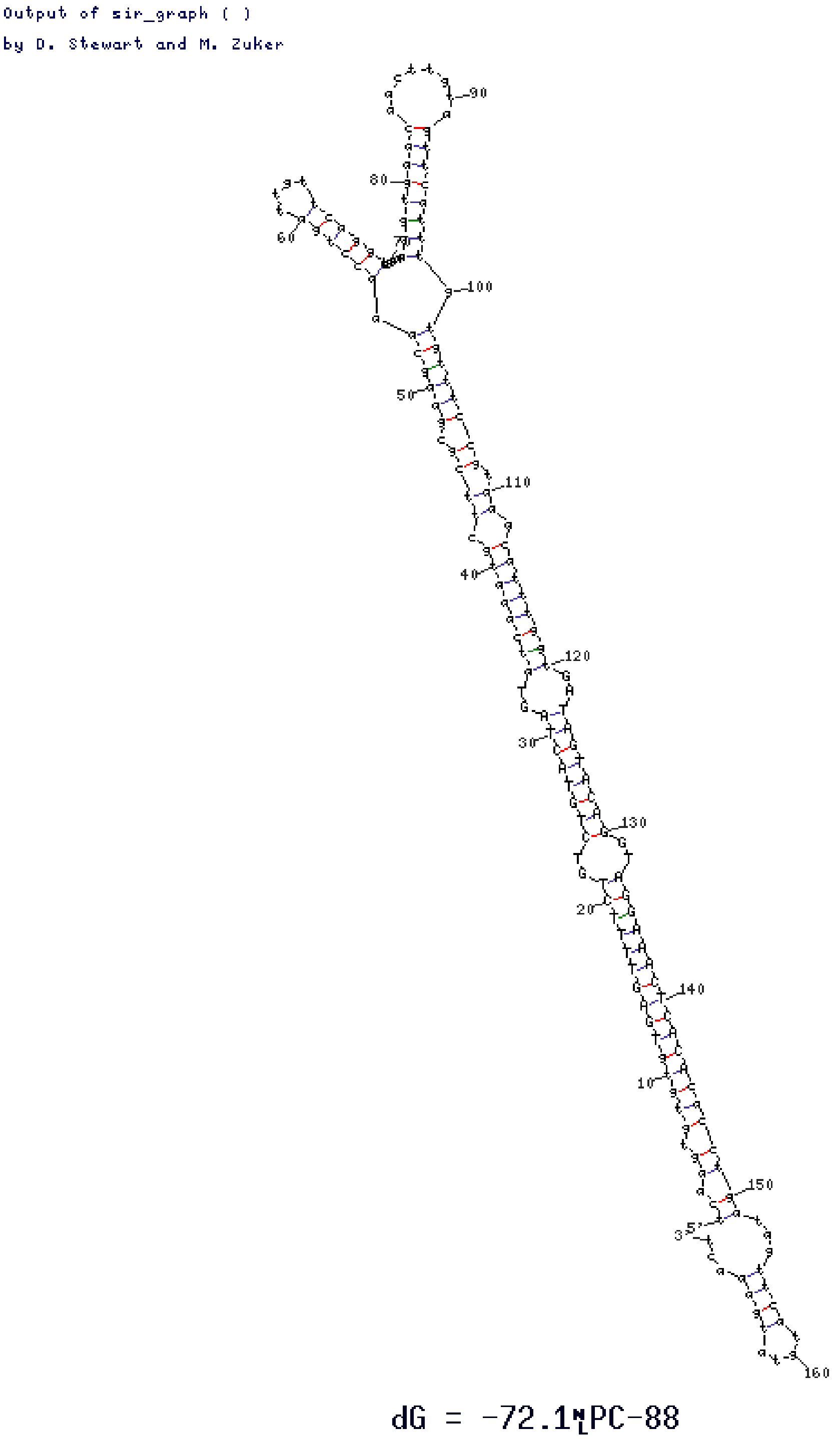

PC-88

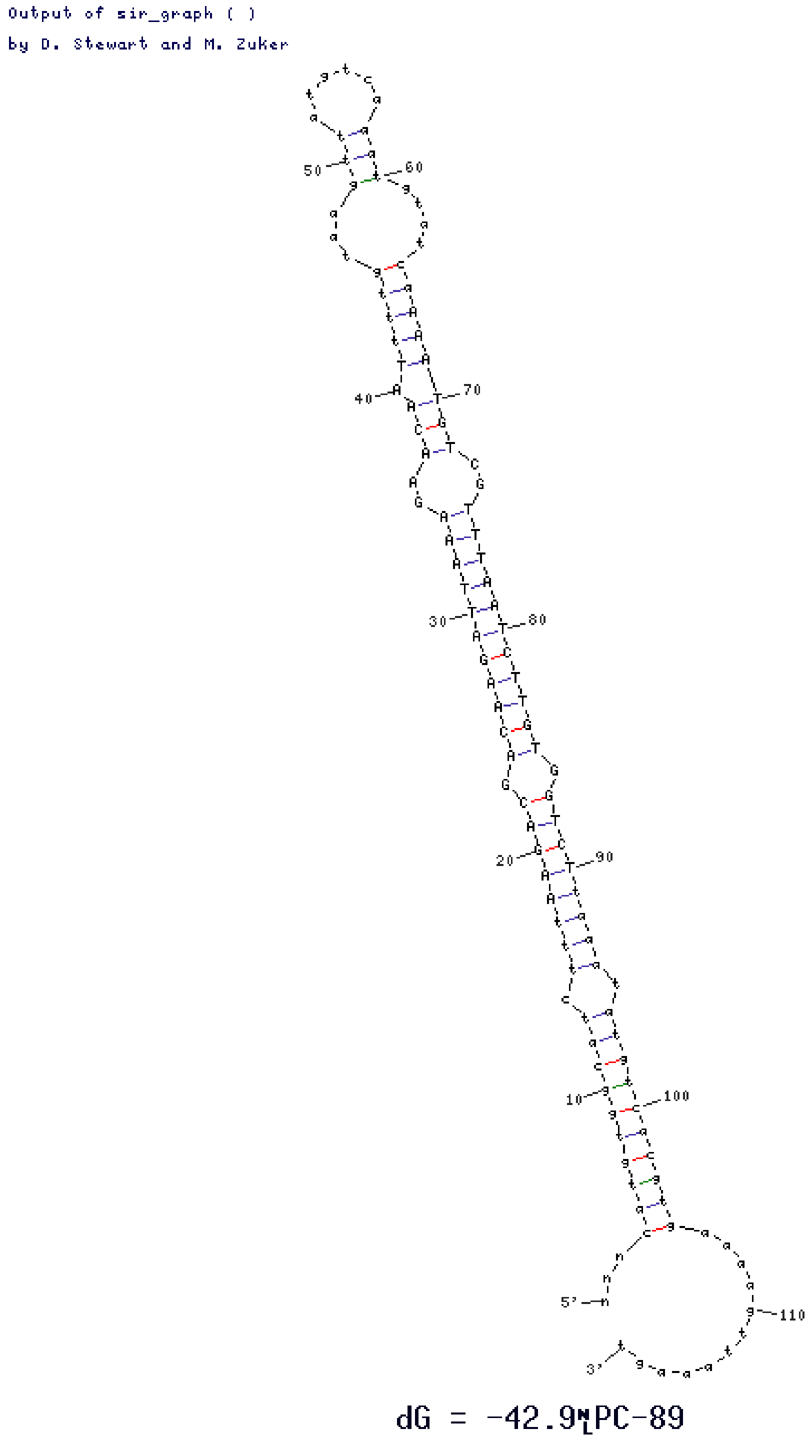

PC-89

Output of sir_graph ( )
by D. Stewart and M. Zuker

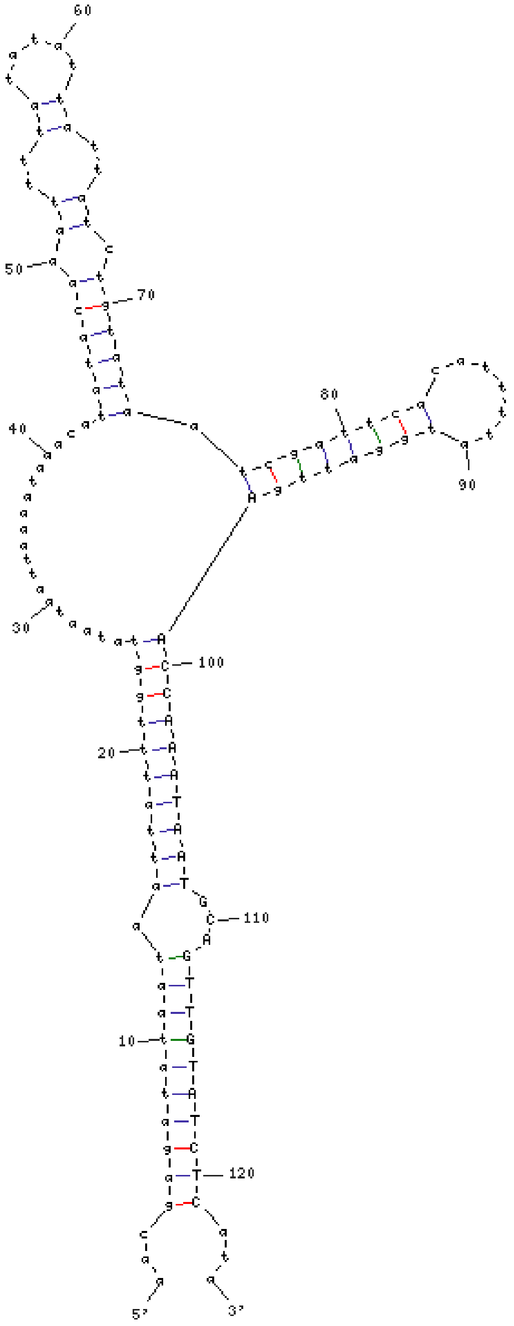

dG = -29.5 PC-90

PC-90

Output of sir_graph ( )
by D. Stewart and M. Zuker

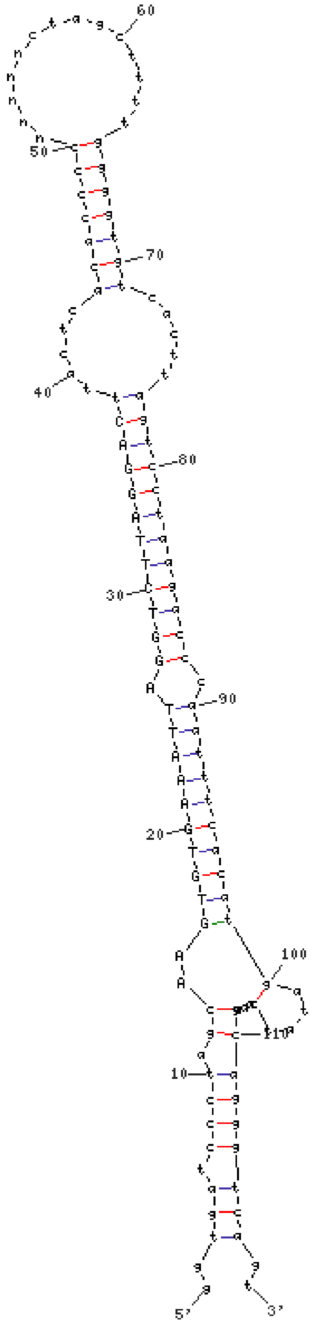

dG = -52.2 PC-91

PC-91

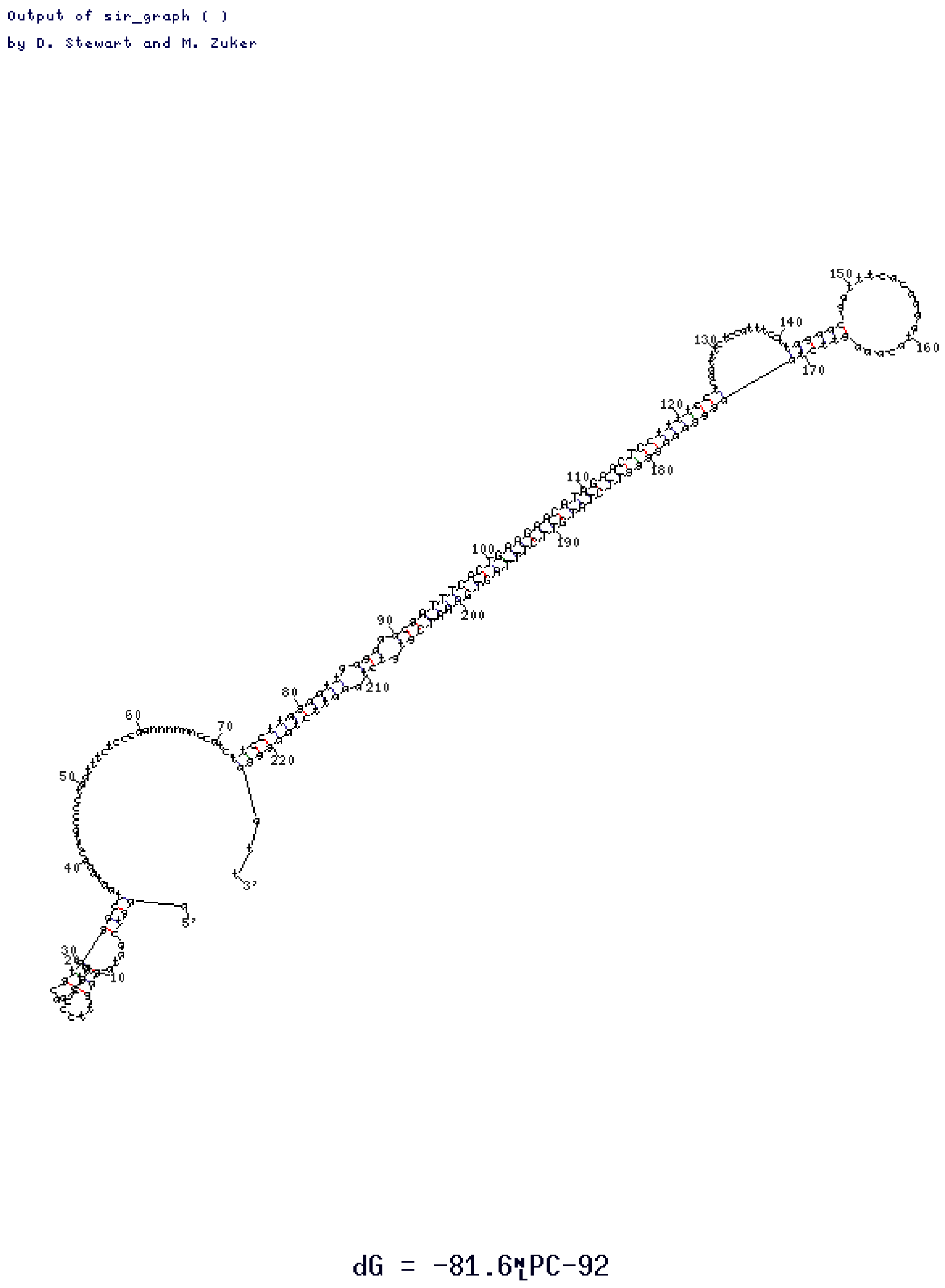

PC-92

Supplementary Figure S1. Folding structures of novel miRNAs in tomato LA2093 at a control temperature (26/18°C, NT), moderately elevated temperature (33/33°C, MET) and acutely elevated temperature (40/40°C, AET)

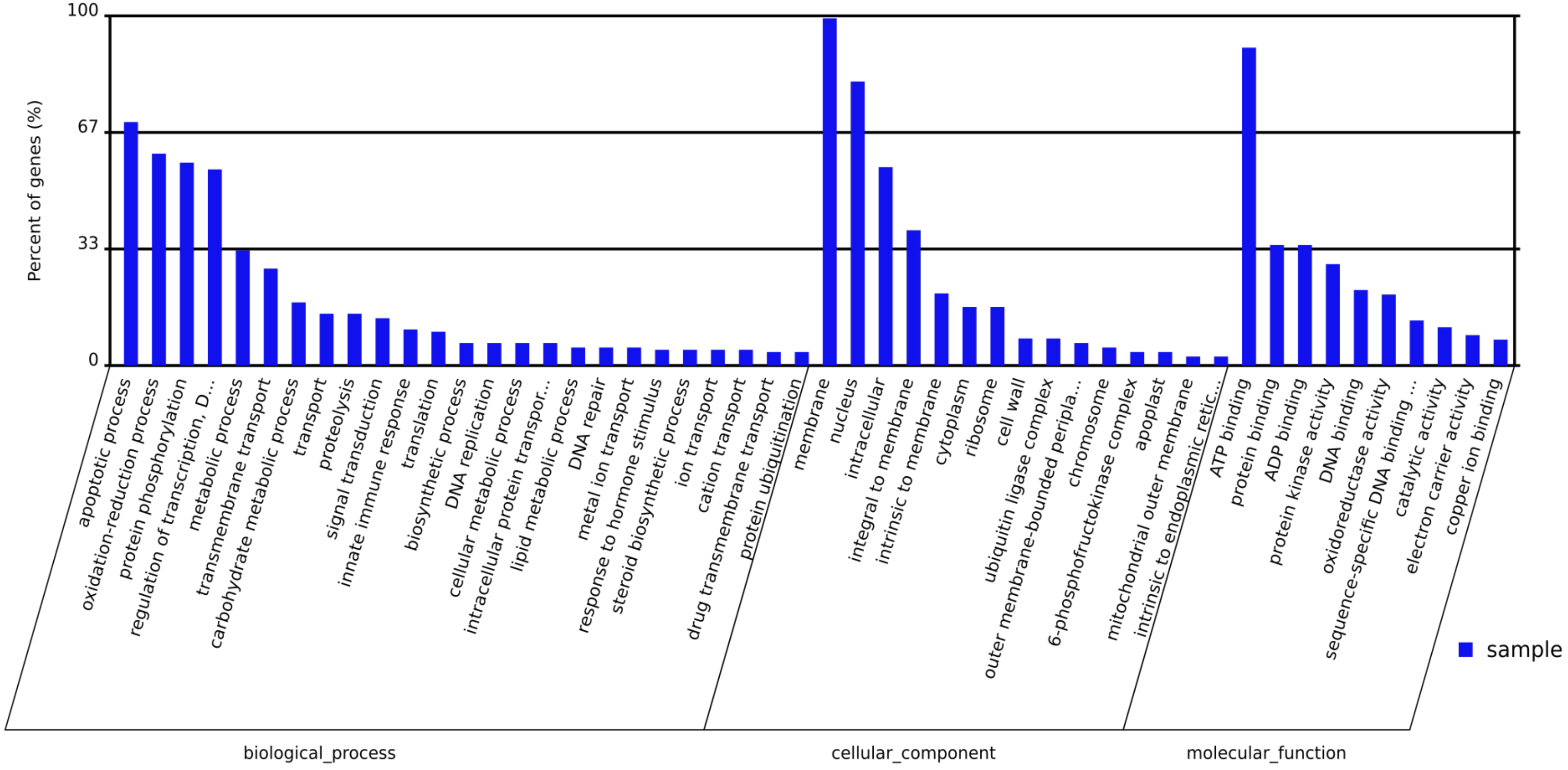

Supplementary Figure S2. GO functional annotation of target genes cleaved by conserved miRNAs in tomato LA2093

‘regulation of transcription, D…’ = ‘regulation of transcription, DNA-dependent’; ‘intracellular protein transport…’ = ‘intracellular protein transport’; ‘outer membrane-bounded peripla…’ = ‘outer membrane-bounded periplasmic space’; ‘intrinsic to endoplasmic retic…’ = ‘intrinsic to endoplasmic reticulum membrane’; ‘sequence-specific DNA binding…’ = ‘sequence-specific DNA binding transcription factor activity’.

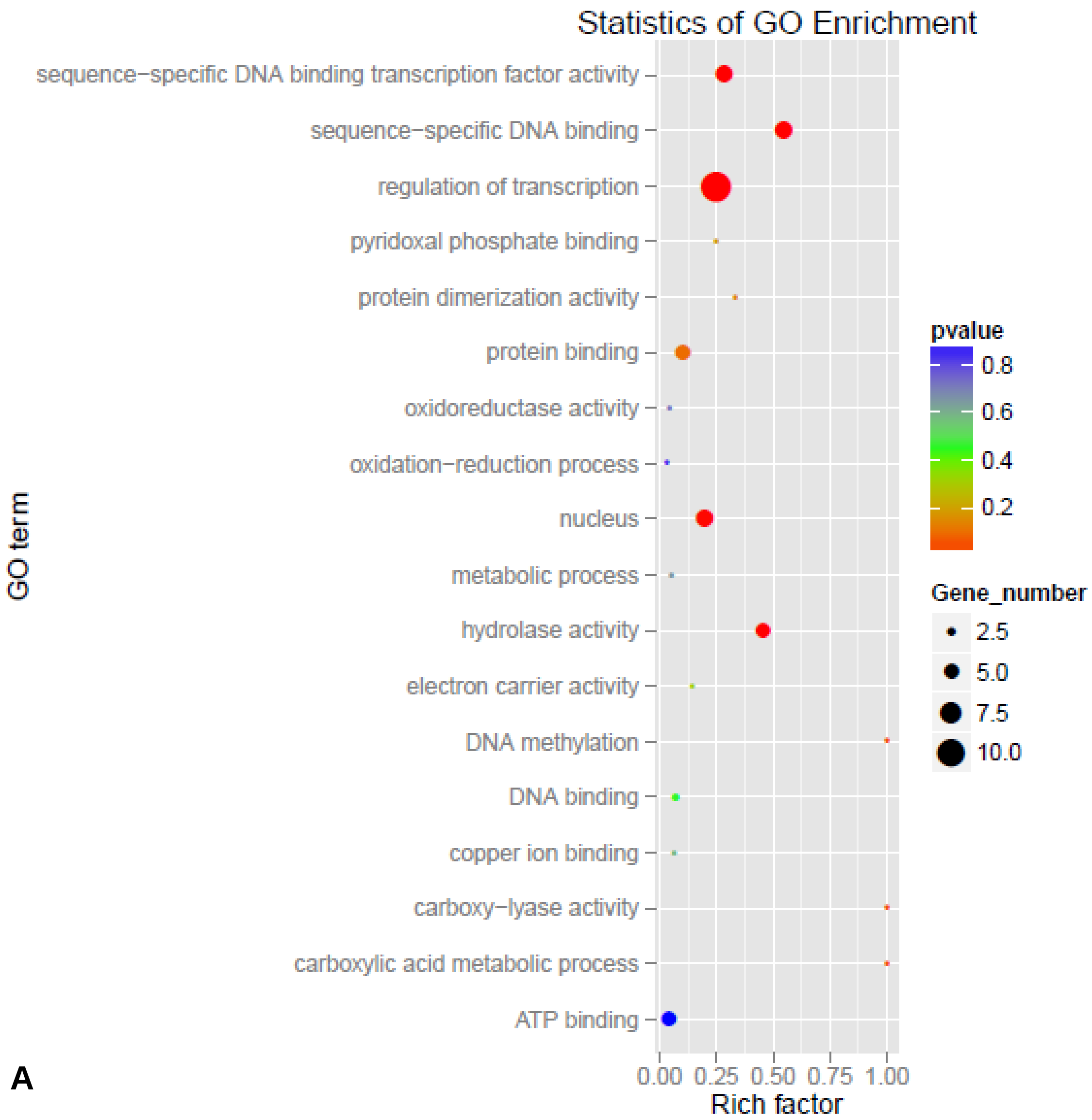

Statistics of GO Enrichment
sequence−specific DNA binding transcription factor activity
sequence−specific DNA binding
regulation of transcription
pyridoxal phosphate binding
protein dimerization activity
protein binding
oxidoreductase activity
oxidation−reduction process
nucleus
metabolic process
hydrolase activity
electron carrier activity
DNA methylation
DNA binding
copper ion binding
carboxy−lyase activity
carboxylic acid metabolic process
ATP binding
GO term
0.00 0.25 0.50 0.75 1.00
Rich factor
pvalue
0.8
0.6
0.4
0.2
Gene_number
2.5
5.0
7.5
10.0

A

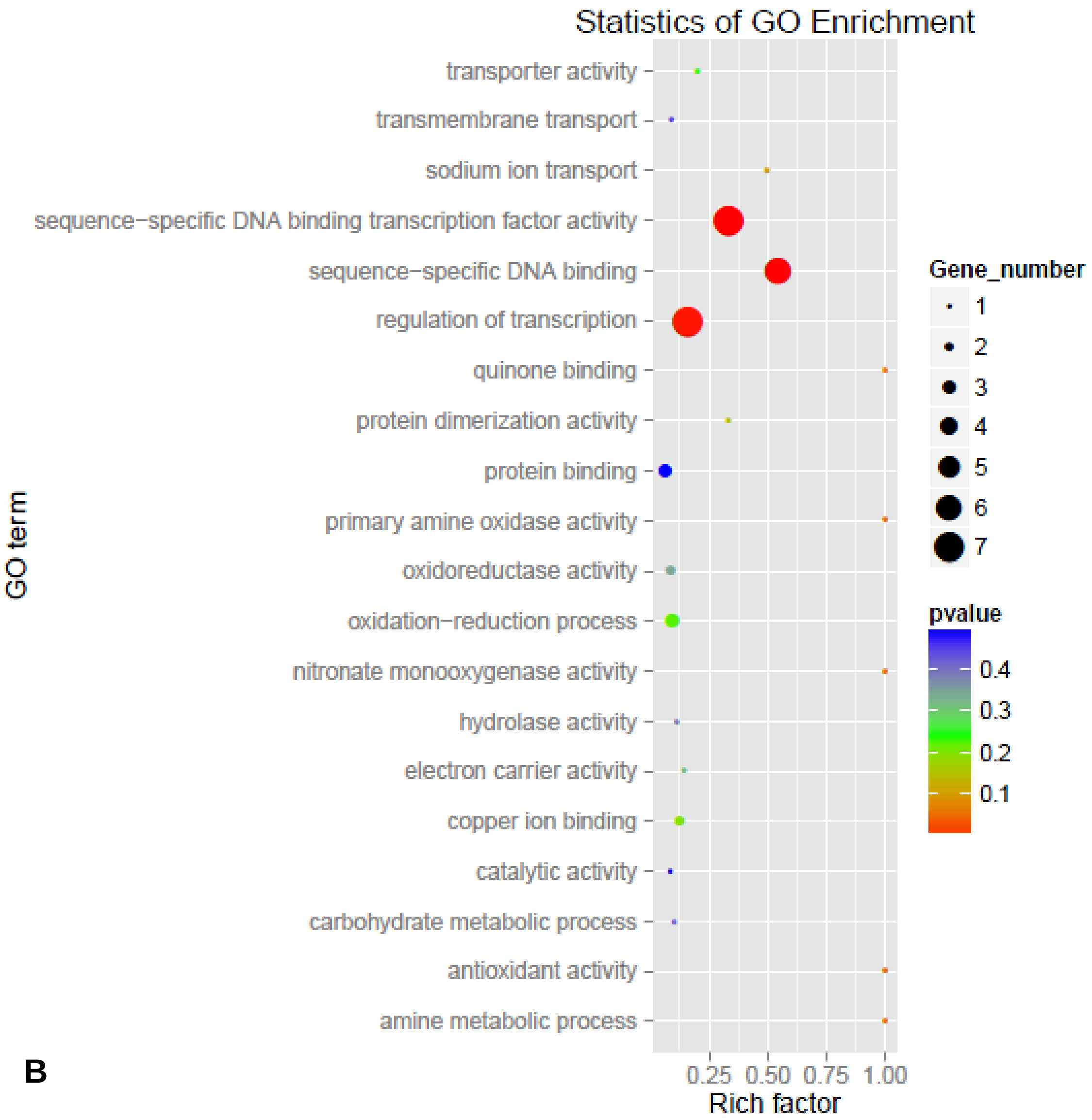

Supplementary Figure S3. GO enrichment analysis of the target genes that predicted by Targetfinder and detected by degrdome sequencing with different expression levels cleaved by miRNAs with different expression levels in tomato LA2093. **(A)** MET vs NT, **(B)** AET vs NT. The x-axis indicated rich factor; the y-axis indicated GO term. The size of the circle indicated the number of genes with the functions; the different colours of the circle indicated different significance levels.

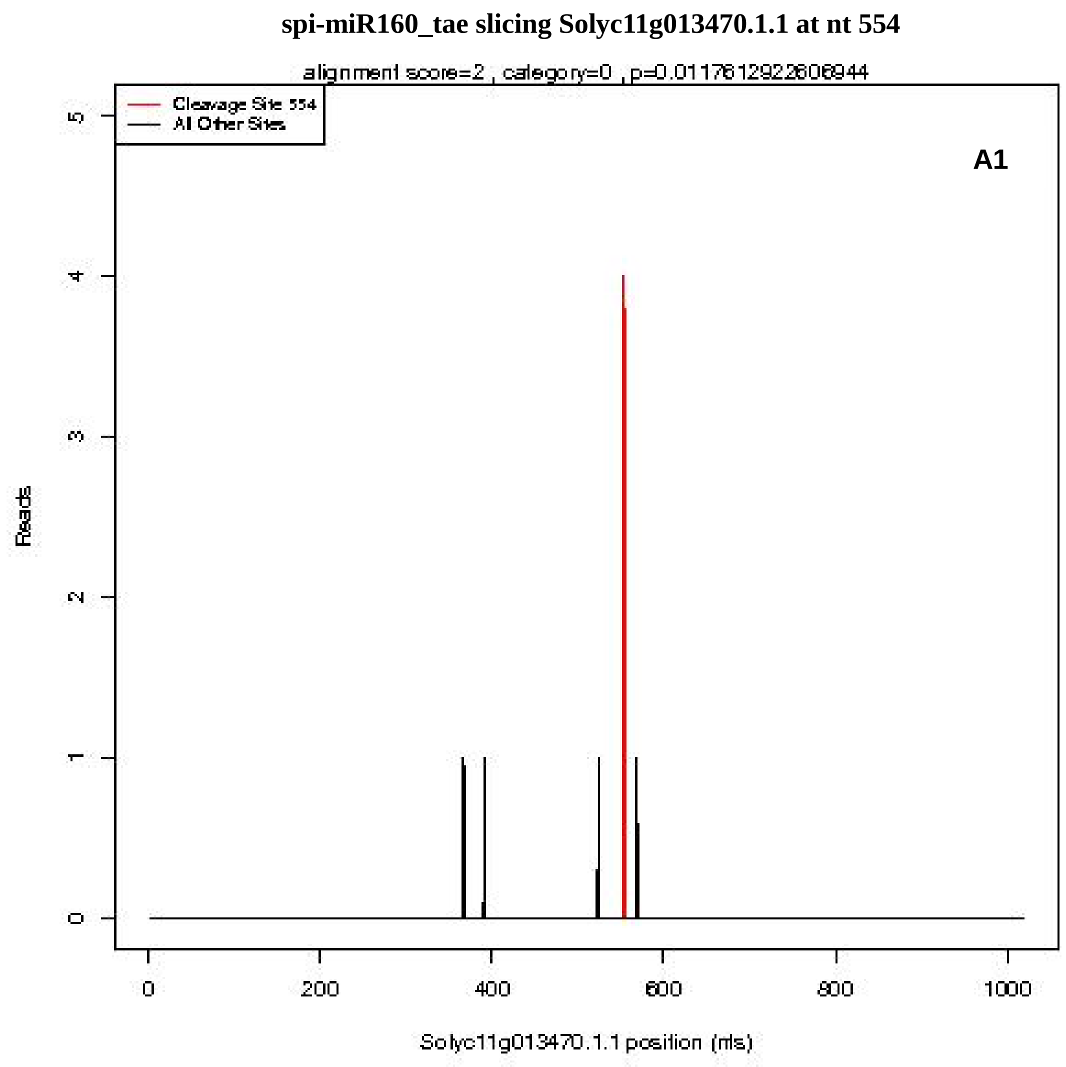
spi-miR160_tae slicing Solyc11g013470.1.1 at nt 554
alignment score=2 , category=0 , p=0.0117612922606944
Cleavage Site 554
All Other Sites
A1
Reads
0
1
2
3
4
5
0
200
400
600
800
1000
Solyc11g013470.1.1 position (nts)

spi-miR166a slicing Solyc02g069830.2.1 at nt 629

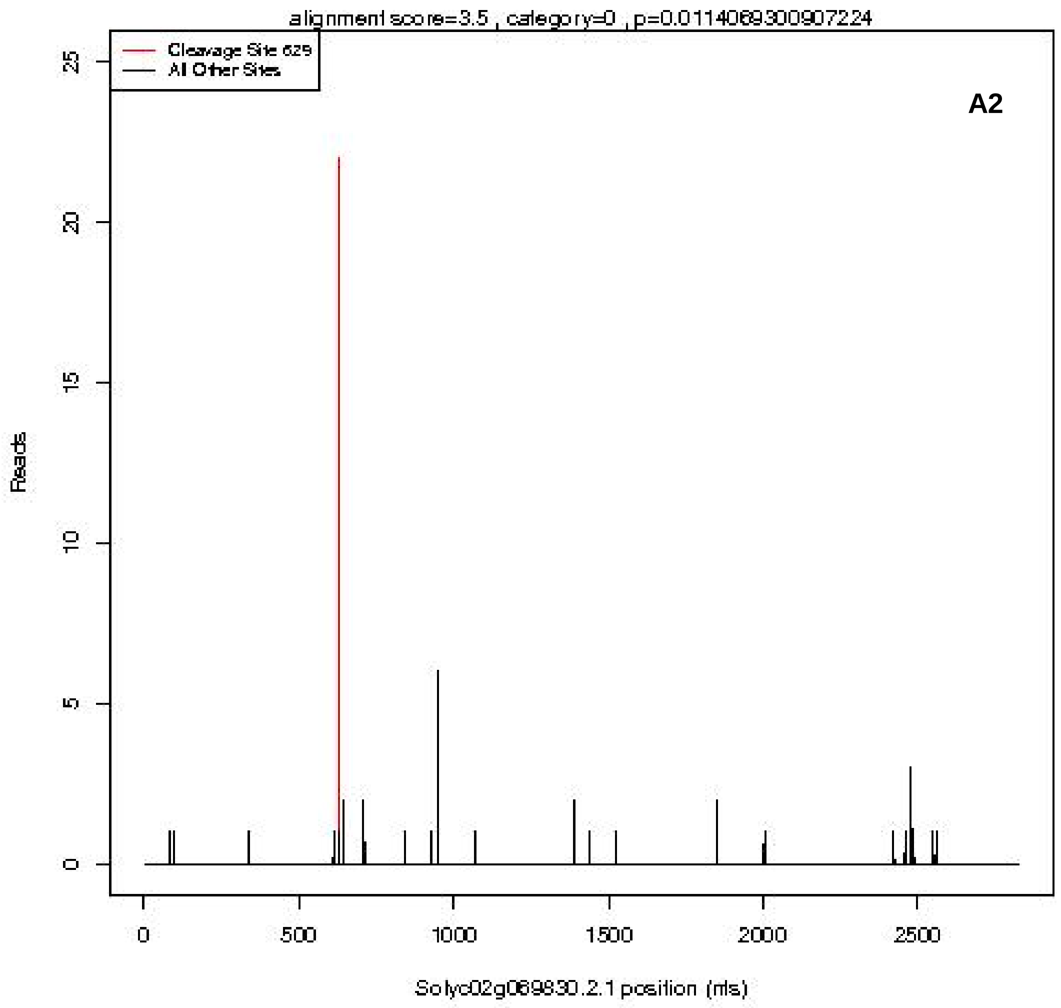

spi-miR166g_osa slicing Solyc02g069830.2.1 at nt 629

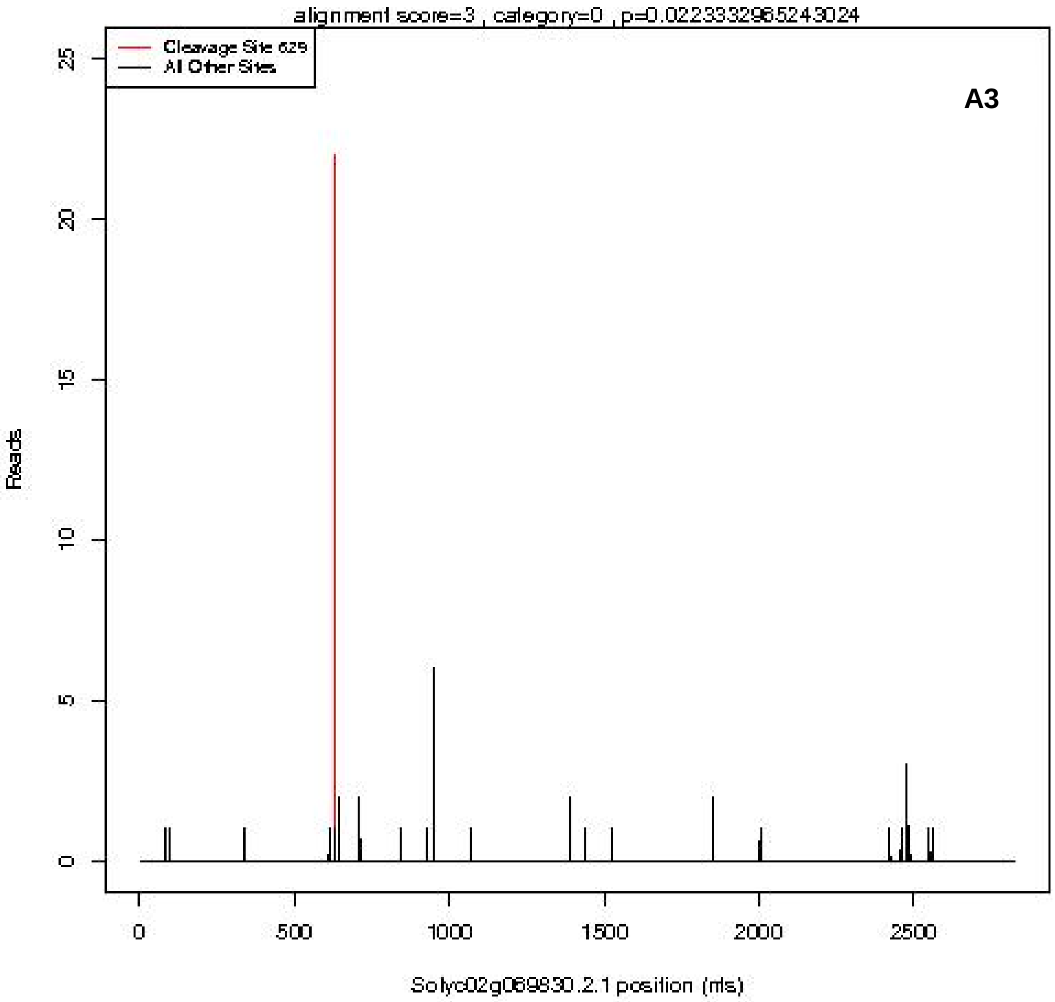

spi-miR160h_ptc slicing Solyc11g013470.1.1 at nt 554

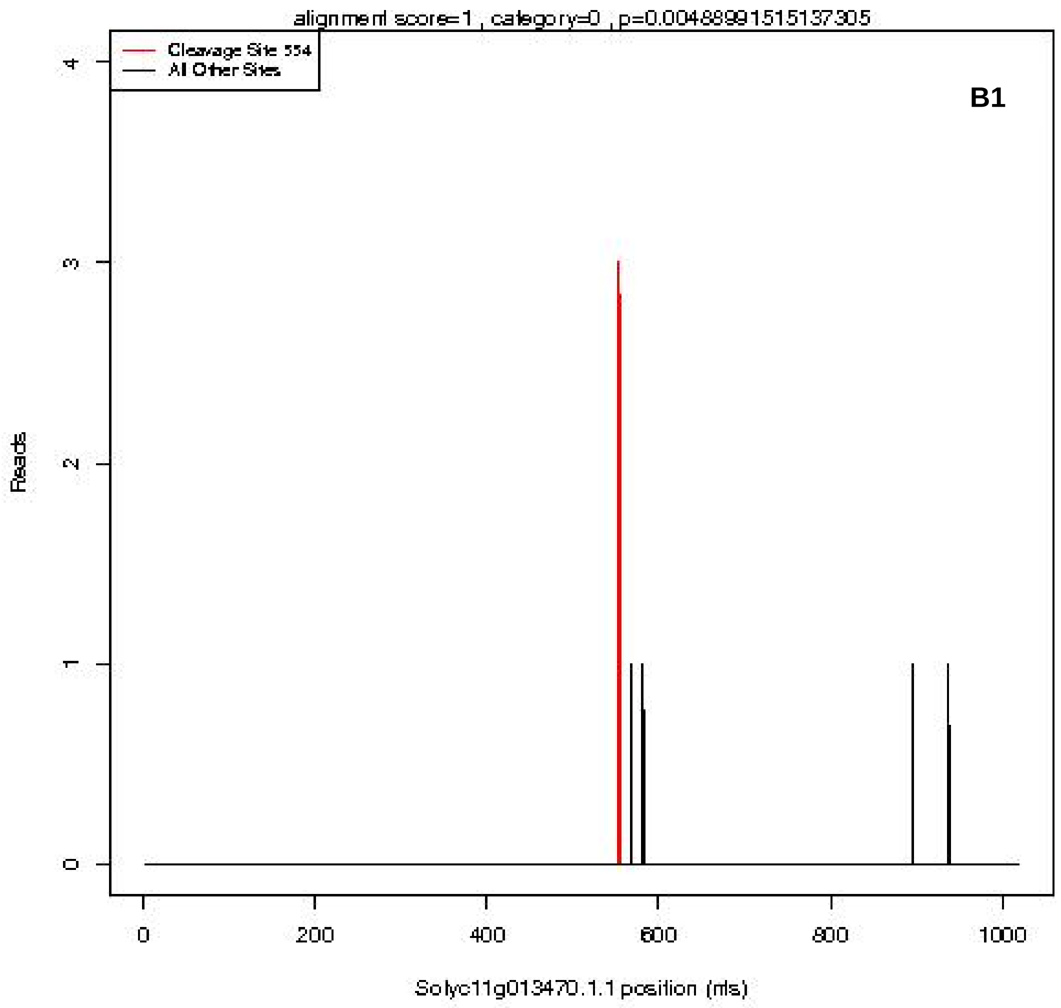

spi-miR166a slicing Solyc02g069830.2.1 at nt 629

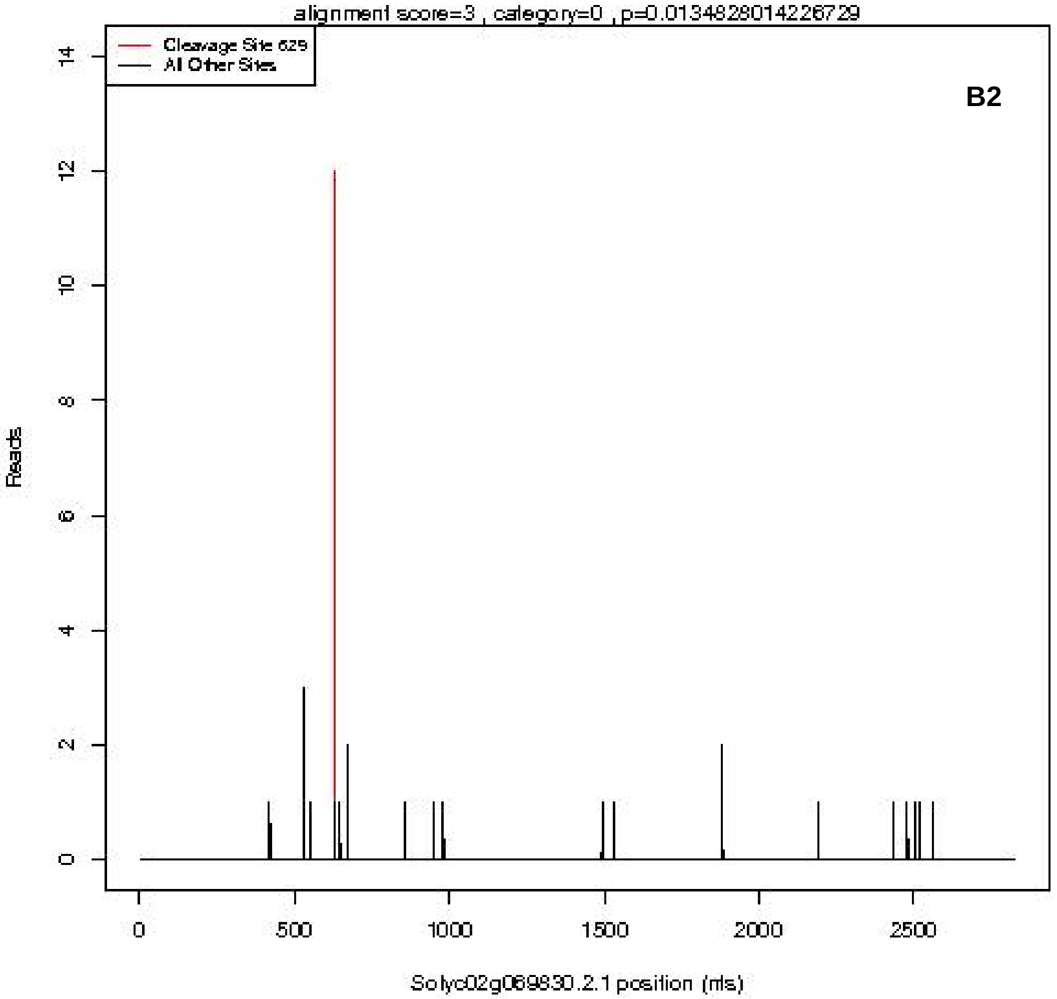

spi-miR396a-5p slicing Solyc12g096070.1.1 at nt 332

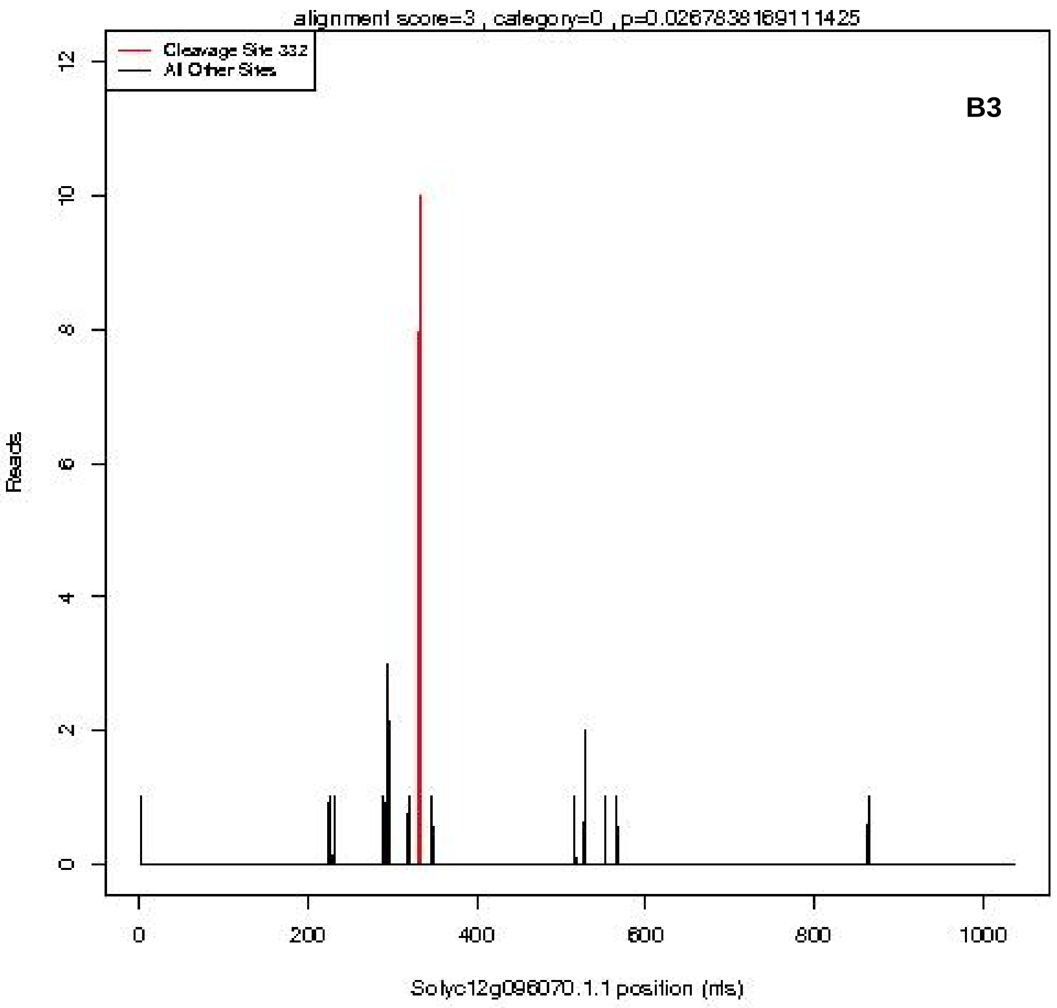

spi-miR160_tae slicing Solyc11g013470.1.1 at nt 554

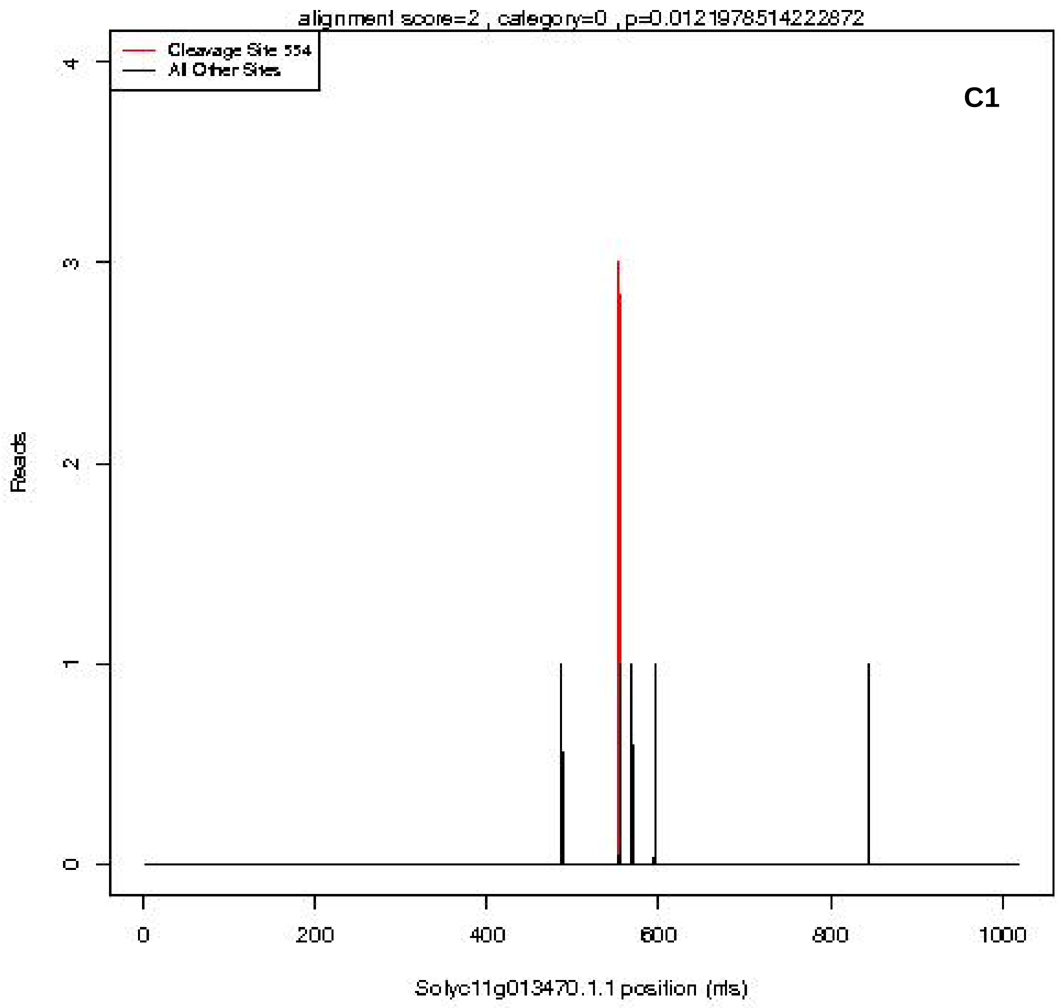

spi-miR166a slicing Solyc02g069830.2.1 at nt 629

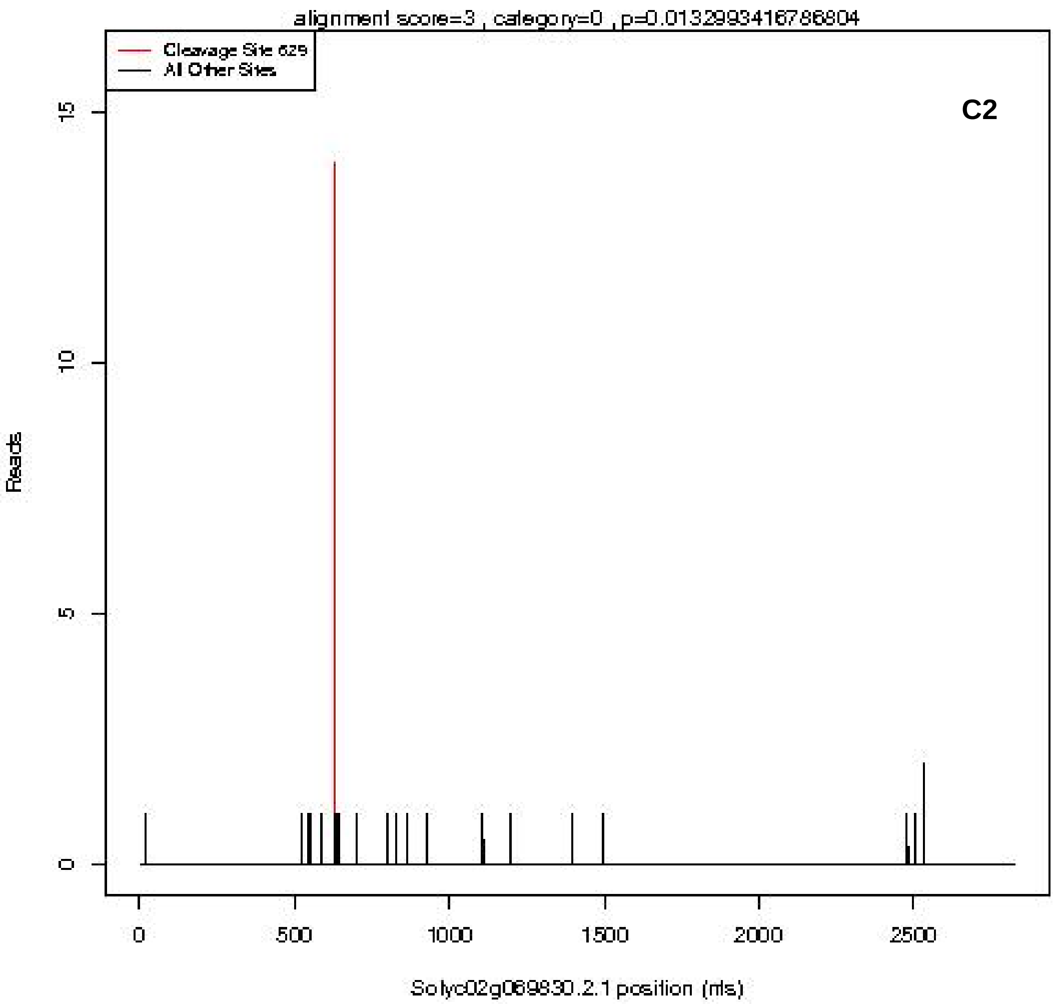

spi-miR396a-5p slicing Solyc12g096070.1.1 at nt 332

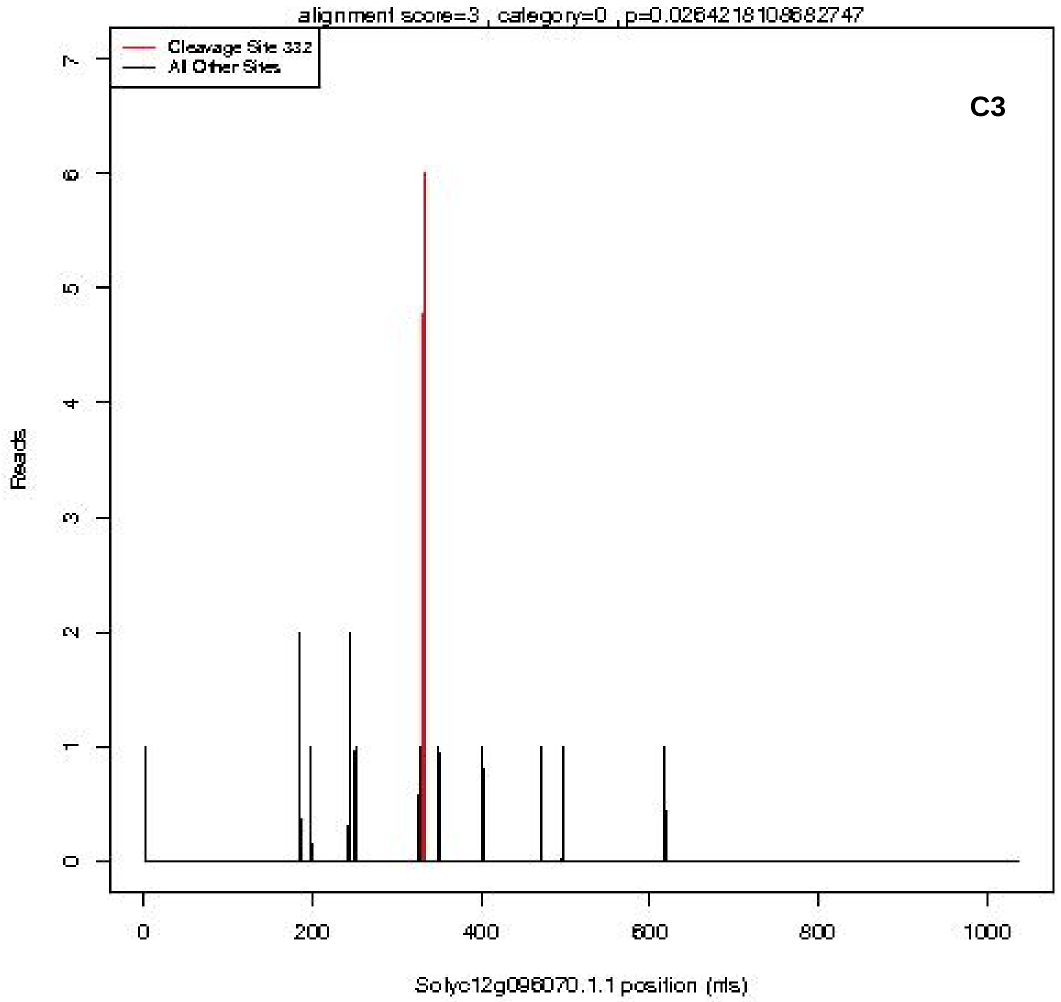

Supplementary Figure S4. Target plots of some mRNAs from category 0 in tomato LA2093 at **(A)** a control temperature (26/18°C, NT), **(B)** moderately elevated temperature (33/33°C, MET) and **(C)** acutely elevated temperature (40/40°C, AET)

Supplementary Table S7. GO functional annotation of the target genes cleaved by novel miRNAs in tomato LA2093

Transcript_ID	Annoation	miRNA	GO term	GO function	GO functionos
Solyc12g014350.1.1	Leucine-rich repeat transmembrane protein kinase	PC-52-5p	GO:0006468	biological process	protein phosphorylation
Solyc01g105370.2.1	Aldolase-type TIM barrel family protein	PC-44-3p	GO:0055114	biological process	oxidation-reduction process
Solyc07g051850.2.1	aspartic proteinase A1	PC-46-5p	GO:0006508	biological process	proteolysis
Solyc07g051850.2.1	aspartic proteinase A1	PC-46-5p	GO:0006629	biological process	lipid metabolic process
Solyc02g091190.2.1	ıdenosyl-L-methionine-dependent methyltransferases superfamily prot	PC-69-3p	GO:0008152	biological process	metabolic process
Solyc12g014350.1.1	Leucine-rich repeat transmembrane protein kinase	PC-52-5p	GO:0004672	molecular function	protein kinase activity
Solyc12g014350.1.1	Leucine-rich repeat transmembrane protein kinase	PC-52-5p	GO:0005515	molecular function	protein binding
Solyc12g014350.1.1	Leucine-rich repeat transmembrane protein kinase	PC-52-5p	GO:0005524	molecular function	ATP binding
Solyc01g105370.2.1	Aldolase-type TIM barrel family protein	PC-44-3p	GO:0016491	molecular function	oxidoreductase activity
Solyc01g105370.2.1	Aldolase-type TIM barrel family protein	PC-44-3p	GO:0018580	molecular function	nitronate monooxygenase activity
Solyc07g051850.2.1	aspartic proteinase A1	PC-46-5p	GO:0004190	molecular function	spartic-type endopeptidase activity
Solyc02g091190.2.1	ıdenosyl-L-methionine-dependent methyltransferases superfamily prot	PC-69-3p	GO:0008168	molecular function	methyltransferase activity

Supplementary Table S8. KEGG pathway analysis of the target genes cleaved by miRNAs in tomato LA2093

Transcript ID	Annoation	miRNA	KEGG ID	KEGG name
Solyc04g008740.2.1	Pyruvate kinase family protein	spi-miR2275c-5p_ata	ko00010	Glycolysis / Gluconeogenesis
Solyc01g073740.2.1	Citrate synthase family protein	spi-miR396b-p5_osa	ko00020	Citrate cycle (TCA cycle)
Solyc03g115820.2.1	D-ribulose-5-phosphate-3-epimerase	spi-miR3630-3p_vvi	ko00030	Pentose phosphate pathway
Solyc03g115820.2.1	D-ribulose-5-phosphate-3-epimerase	spi-miR3630-3p_vvi	ko00040	Pentose and glucuronate interconversions
Solyc04g008740.2.1	Pyruvate kinase family protein	spi-miR2275c-5p_ata	ko00230	Purine metabolism
Solyc03g005260.2.1	ATP sulfurylase 1	spi-miR395a_stu	ko00230	Purine metabolism
Solyc03g005730.2.1	›propyl malate isomerase large subuni	spi-miR384-5p_stu	ko00290	Valine, leucine and isoleucine biosynthesis
Solyc03g005260.2.1	ATP sulfurylase 1	spi-miR395a_stu	ko00450	Selenoamino acid metabolism
Solyc08g063080.2.1	sulfoquinovosyldiacylglycerol 1	spi-miR9470-3p	ko00520	mino sugar and nucleotide sugar metabolism
Solyc09g020190.2.1	non-specific phospholipase C1	spi-miR9470-3p	ko00562	Inositol phosphate metabolism
Solyc09g020190.2.1	non-specific phospholipase C1	spi-miR9470-3p	ko00564	Glycerophospholipid metabolism
Solyc04g008740.2.1	Pyruvate kinase family protein	spi-miR2275c-5p_ata	ko00620	Pyruvate metabolism
Solyc04g008740.2.1	Pyruvate kinase family protein	spi-miR2275c-5p_ata	ko00710	Carbon fixation in photosynthetic organisms
Solyc05g054080.2.1	ubiquitin protein ligase 5	spi-miR9471a-3p	ko04120	Ubiquitin mediated proteolysis
Solyc06g061000.2.1	casein kinase II beta subunit 4	spi-miR319-p5_stu	ko04310	Wnt signaling pathway
Solyc06g061000.2.1	casein kinase II beta subunit 4	spi-miR319-p5_stu	ko04520	Adherens junction
Solyc06g061000.2.1	casein kinase II beta subunit 4	spi-miR319-p5_stu	ko04530	Tight junction

Supplementary Table S9. Specific qRT-PCR primers for the miRNAs identified in tomato LA2093

Number	miRNAs	Stem-loop primer	Length of reverse transcribed fragments	qRT-PCR primer	Sequence (5'to 3') for qRT-PCR	Length of amplified fragment
1	spi-miR159	GTCGTATTCTGTGCTGGGTGGAACTGTCTCGCACTGAATACGACGAGCTC	50	F	CGGACGAGTTTGGATTGA	60
				R	TATTCTGTGCTGGGTGGA	
2	spi-miR168a-5p	GTCGTATTCTGTGCTGGGTGGAACTGTCTCGCACTGAATACGACGTCCCG	50	F	CGGACGAGTCGCTTGGTG	62
				R	CGTATTCTGTGCTGGGTGGA	
3	spi-miR171d	GTCGTATTCTGTGCTGGGTGGAACTGTCTCGCACTGAATACGACCGTGAT	50	F	AAGGACGAGTTGAGCCGC	60
				R	GTATTCTGTGCTGGGTGG	
4	spi-miR319a	GTCGTATCCAGTGCAGGGTCCGAGGTATTCGCACTGGATACGACAAGGAG	50	F	ACGGCGTTGGACTGAAGG	60
				R	CGCAGGGTCCGAGGTATT	
5	spi-miR408b-3p_stu	GCGTGGTCCACACCACCTGAGCCGCCACGACCACGCAGCCAGGG	44	F	CAACAATGCACTGCCTCTTCC	57
				R	TCCACACCACCTGAGCCG	
6	PC-49-3p	GTCGTATCCAGTGCAGGGTCCGAGGTATTCGCACTGGATACGACCACGTT	50	F	CCGCCGCTATGAGATAAGTTC	63
				R	CAGTGCAGGGTCCGAGGTA	
7	PC-65-3p	GTCGTATCCAGTGCAGGGTCCGAGGTATTCGCACTGGATACGACAGGGGT	50	F	CGGTCAACATGTGTTCTCAGGT	65
				R	CAGTGCAGGGTCCGAGGTAT	
Reference	U6snRNA	GTGCAGGGTCCGAGGTTTTGGACCATTTCTCGAT	/	F	GGAACGATACAGAGAAGATTAGCA	/
				R	GTGCAGGGTCCGAGGT	

Supplementary Table S10. Specific qRT-PCR primers for the target genes cleaved by miRNAs in tomato LA2093

Number	Target genes	Primer	Sequence (5'to 3')	Length of amplified fragment	miRNAs
1	Solyc12g014120.1.1	F	TTACCACCACCATCAACAAT	152	spi-miR159
		R	ACAGACGACCAACTTAACTC		
2	Solyc11g013470.1.1	F	CGCCAATCCCACAGTTTC	171	spi-miR160a
		R	GGGCTACTGAGTCCAATCGT		
3	Solyc06g072300.2.1	F	TCTTGATGATGATGATGGA	169	spi-miR168a-5p
		R	CTCACGCAGAACAATATC		
4	Solyc11g013150.1.1	F	CAGGTGACTCCAATAACAATC	189	spi-miR171d
		R	AATACGCAGCCAATCTCT		
5	Solyc02g070550.2.1	F	TCAAGCGAAGAAGAAGAAGAAGGA	162	spi-miR8029_stu
		R	GAAGCGGTCTGAGAAGGTATAGG		
6	Solyc02g091190.2.1	F	CAGCGTGAGATTGACATT	182	PC-69-3p
		R	AAGAACTTGACATTGGTGAA		
Reference	Actin	F	GAAATAGCATAAGATGGCAGACG	159	/
		R	ATACCCACCATCACACCAGTAT		
